# Identifying major impact factors affecting the continuance intention of mHealth: a systematic review and multi-subgroup meta-analysis

**DOI:** 10.1038/s41746-022-00692-9

**Published:** 2022-09-15

**Authors:** Tong Wang, Wei Wang, Jun Liang, Mingfu Nuo, Qinglian Wen, Wei Wei, Hongbin Han, Jianbo Lei

**Affiliations:** 1 Department of Medical Informatics, School of Public Health, Jilin University, Changchun, Jilin Province China; 2 IT Center, Second Affiliated Hospital, School of Medicine, Zhejiang University, Hangzhou, Zhejiang Province China; 3 School of Public Health, Zhejiang University, Hangzhou, Zhejiang Province China; 4 Key Laboratory of Cancer Prevention and Intervention, China National Ministry of Education, School of Medicine, Zhejiang University, Hangzhou, Zhejiang Province China; 5 Institute of Medical Technology, Health Science Center, Peking University, Beijing, China; 6 Department of Oncology, The Affiliated Hospital of Southwest Medical University, Luzhou, Sichuan Province China; 7 Department of Gastroenterology, Wangjing hospital, Beijing, China; 8 Key Laboratory of Traditional Chinese Medicine Treatment of Functional Gastrointestinal Diseases, Chinese Academy of Medical Sciences, Beijing, China; 9 Center for Medical Informatics, Health Science Center, Peking University, Beijing, China; 10 School of Medical Informatics and Engineering, Southwest Medical University, Luzhou, Sichuan Province China

**Keywords:** Health services, Human behaviour

## Abstract

The mobile health (mHealth) industry is an enormous global market; however, the dropout or continuance of mHealth is a major challenge that is affecting its positive outcomes. To date, the results of studies on the impact factors have been inconsistent. Consequently, research on the pooled effects of impact factors on the continuance intention of mHealth is limited. Therefore, this study aims to systematically analyze quantitative studies on the continuance intention of mHealth and explore the pooled effect of each direct and indirect impact factor. Until October 2021, eight literature databases were searched. Fifty-eight peer-reviewed studies on the impact factors and effects on continuance intention of mHealth were included. Out of the 19 direct impact factors of continuance intention, 15 are significant, with attitude (β = 0.450; 95% CI: 0.135, 0.683), satisfaction (β = 0.406; 95% CI: 0.292, 0.509), health empowerment (β = 0.359; 95% CI: 0.204, 0.497), perceived usefulness (β = 0.343; 95% CI: 0.280, 0.403), and perceived quality of health life (β = 0.315, 95% CI: 0.211, 0.412) having the largest pooled effect coefficients on continuance intention. There is high heterogeneity between the studies; thus, we conducted a subgroup analysis to explore the moderating effect of different characteristics on the impact effects. The geographic region, user type, mHealth type, user age, and publication year significantly moderate influential relationships, such as trust and continuance intention. Thus, mHealth developers should develop personalized continuous use promotion strategies based on user characteristics.

## Introduction

The mobile health (mHealth) industry is an enormous global market worth more than 50 billion USD, as the main means for health management is booming, with a wide range of products and a growing userbase; however, it also faces new challenges. Mobile health (mHealth) is defined as “the medical and public health practice supported by mobile devices, such as mobile phones, patient monitoring devices, personal digital assistants (PDAs), and other wireless devices” by the World Health Organization (WHO).^1^ It can improve the capacity and efficiency of medical services, monitor patients’ daily activities and physical indicators,^2^ improve personal health status by influencing personal health behaviors and avoiding health risks,^3^ and reduce rehospitalization and hospitalization costs by 63% and disease prevention costs by 33%.^4^ Considering these advantages, mHealth technologies and services represent the future of the health industry. The global mHealth market is valued at 51.635 billion USD in 2020 and is expected to grow at a compound annual rate of 25%, reaching 225.765 billion USD in 2026.^5^ Currently, more than 300,000 mHealth apps are available in the market, with approximately 40% of the smartphone users using mHealth apps.^6^ The coronavirus disease pandemic has resulted in the massive use of mHealth, with a survey showing that the downloads of mHealth apps were increased by 25% during the pandemic.^7^ Although mHealth has been developed rapidly and its use is widely accepted, this type of technology faces a major challenge, i.e., whether users will drop out or continue using it.^8^

Continuous use is of great significance to mobile health, as the initial short-term use of mHealth services is unlikely to lead to the achievement of long-term health management goals and improve the user health status. Moreover, short-term use is not conducive to the survival of mHealth services and technology in the market. In this context, Bhattacherjee pointed out that the long-term success of information technology or information systems does not depend on the initial adoption but on continuance.^9^ In a clinical trial setting, most health management services based on mHealth interventions should be used for more than 3 weeks to show effective results.^10^ However, a large real-world observational study found that only 2.58% of the 189,770 users who downloaded a mHealth app were active users for at least one week^11^; Similarly, other studies have reported that up to 80% of mHealth users logged in to the service less than twice after downloading.^12^ Therefore, although the positive outcomes of mHealth have been demonstrated in a clinical trial setting, there is limited evidence of its real-world benefits owing to poor continuance.

Continuance also has a major impact on mHealth companies. A previous study showed that 30% of mHealth apps are uninstalled within a month of download.^6^ Although the mHealth app market is huge, only 7% of mHealth apps have more than 50,000 active users monthly, and 62% of the apps reported fewer than 1000 active users monthly (active users were defined as those who use the app at least once a month).^13^ In 2020, app companies lost an average of $57,000 monthly because of uninstallation.^14^ Therefore, improving the continuance of mHealth services in the real world is a prerequisite for ensuring the effectiveness of health management and promoting the development of this type of technology.

Continuance intention is an important driving force for long-term use, which is defined as user’s willingness and tendency to continue using the mHealth.^15^ Therefore, relevant studies on the factors and mechanisms of continuance intention of mHealth services have gradually been increased. Studies on the various types of mHealth services, such as preventive health care or chronic disease management apps,^16,17^ wearable devices,^18^ and telemedicine, have been conducted.^19^ The amount of relevant literature is growing, with different results from different theories, models, and populations being reported.^20–23^ For example, Paré et al. aimed to explore the intention to continue using wearable devices for health management and found that the perceived ease of use had a significant positive impact on the continuance intention (β = 0.33, *P* < 0.01)^20^. In contrast, Cho and Lee found that its effect was not statistically significant (β = 0.10, *P* = 0.15).^21^ These contradictory findings make it difficult for researchers to deeply understand the mechanisms of mHealth continuance intention. Therefore, it is necessary to comprehensively determine the factors affecting the continuance of mHealth services.

Although, systematic reviews on the continuance of mHealth services or information systems have been published, there has been no quantitative and comprehensive meta-analysis study on the impact factors of the continuance intention to use mHealth. Khalil et al. conducted a systematic review on mHealth app continuance use intentions and comprehensively described the theoretical model used in the study and the distribution of impact factors. However, as it was only a descriptive review, the study could not explain the contradictory phenomena of the influence effects.^6^ A meta-analysis combines the quantitative results of multiple empirical studies to comprehensively assess the overall effect of impact factors and is more rigorous than descriptive systematic reviews and provides in-depth insights.^24^ Frank et al. conducted a meta-analysis of the impact factors of continuance intention of information systems; and identified five optimal influencing factors after integrating the influence effects.^25^ However, the use case scenarios for information systems in the study by Frank et al. are different from those for mHealth services. Similarly, user perceptions are also different. Therefore, the results of Frank et al. cannot fully explain the reasons for the continued use of mHealth services.

To address the above-mentioned limitations, this study focuses on mHealth users, including the general population and patients with diseases. We aim to integrate the effects of direct impact factors on users’ continuance intention, and then use the direct impact factors as intermediary variables to integrate the effects of indirect factors to explore the comprehensive effect of all impact factors. Moreover, we identify the main impact factors of continuance intention by conducting a meta-analysis. On this basis, subgroup analyses are performed to explore the moderating effects of the study design and population on the influence relationship, and to provide a reference for establishing a personalized strategy for the continuous use of mHealth.

## Results

### Literature search and study characteristics

**Fig. 1:**
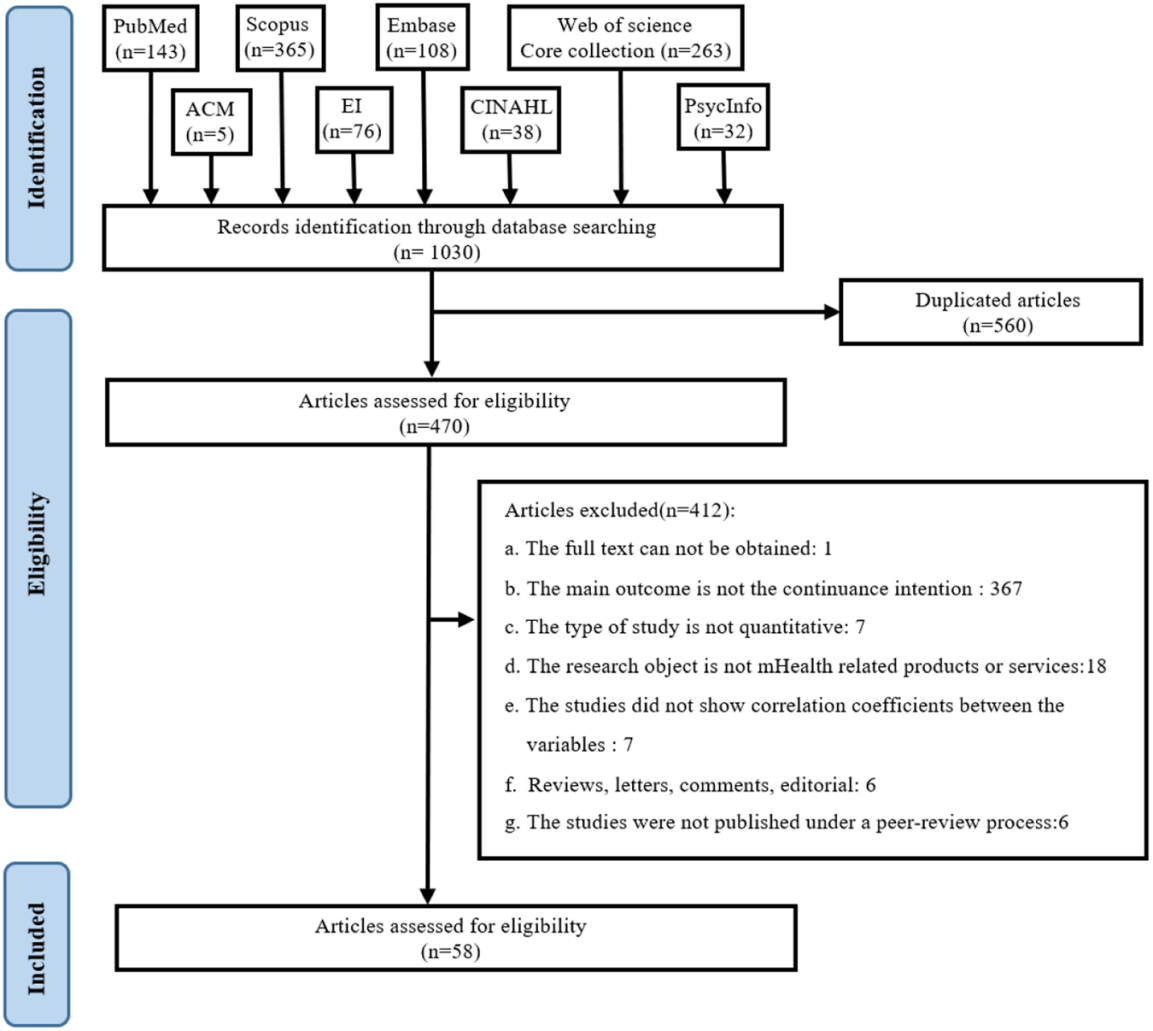
Screening of studies included in the meta-analysis and systematic review. Using the Preferred Reporting Items for Systematic Reviews and Meta-Analyses as a screening process, a total of 58 studies were included in the study.

The study selection process and the results of the literature search are depicted in Fig. 1. Using our search strategy, we identified 1,030 articles in eight databases. After removing duplicate articles, 470 articles remained. After applying further exclusion criteria, a total of 58 cross-sectional studies were included in our analysis, including 19 direct impact factors of continuance intention which have been explored more than three times.

The characteristics of these 58 studies are summarized in Table 1. The three countries which were the most highly active in research were China (25 studies), South Korea (7 studies) and the United States (6 studies). The most frequently used theories and models were the Expectation Confirmation Model of IS Continuance (ECM-ISC, 15 times), Technology Acceptance Model (TAM, 9 times) and the Unified Theory of Acceptance and Use of Technology (UTAUT, 6 times). All studies used self-reported continuance intention and use feeling, which was collected using questionnaires. The study quality scores ranged from 4 to 16, with a mean (standard deviation) score of 12.43 (2.50) (Supplementary Table 1). Kendall’s consistency coefficient was used to evaluate the consistency of researchers’ scores, which was 0.971 indicating a strong consistency.

**Table 1:** Research studies included in meta-analysis.

Study	Country	Sample	Theory	User type	Age characteristics	mHealth type
Park et al.^17^	South Korea	201	Social Cognitive Theory	GP	YI	HPP
Hamari and Koivisto^41^	Finland	200	Self-Determination Theory	GP	EP	HPP
Lee and Choo^39^	The United States	142	Uses and Gratifications theory	GP	YI	HPP
Rho et al.^42^	South Korea	101,81	—	PA	EP	DMRC
Li et al.^43^	China	211	Expectation Confirmation Theory	GP	EP	HPP
Gu et al.^22^	China	494	Expectation Confirmation Model of IS Continuance	PA	EP	DMRC
Choo et al.^44^	China	346	Technology Acceptance Model; Investment Model	GP	EP	HPP
Ahmad et al.^18^	Bangladesh	223	Technology Acceptance Model	PA	OI	WD
Hsiao and Chen^45^	Taiwan, China	201	Expectation Confirmation Model of IS Continuance	HCP	EP	HPP
Kaium et al.^46^	Bangladesh	400	the Unified Theory of Acceptance and Use of Technology; Information System Success Model; Expectation Confirmation Model of IS Continuance	GP	OI	HPP
Akter et al.^47^	Bangladesh	216	the Theory of Reasoned Action; Trustworthiness	PA	EP	DMRC
Meng et al.^48^	China	232	Trustworthiness	GP	OI	HPP
Zhang et al.^49^	China	273	Expectation Confirmation Model of IS Continuance; Elaboration Likelihood Model	GP	YI	HPP
Esmaeilzadeh^50^	The United States	450	Identity theory	GP	EP	WD
Yin et al.^51^	China	328	Social Support Theory	GP	EP	HPP
Esmaeilzadeh^52^	The United States	364	Identity theory	GP	EP	HPP
Cho^53^	South Korea	343	Expectation Confirmation Model of IS Continuance; Technology Acceptance Model	GP	EP	HPP
Wang et al.^15^	China	307	Expectation Confirmation Model of IS Continuance; Self-Determination Theory	GP	EP	HPP
Uei et al.^54^	Taiwan, China	120	the Theory of Reasoned Action	PA	OI	DMRC
Lee and Kim^55^	South Korea	363	—	GP	EP	HPP
Akter et al.^31^	Bangladesh	283	SERVQUAL	GP	EP	DMRC
Sharma and Khadka^56^	The United States	246	Theory of Need to Belong; Theory of Empowerment	PA	EP	DMRC
Devina et al.^57^	Indonesia	146	—	GP	YI	DMRC
Damberg^58^	the United Kingdom	591	the Unified Theory of Acceptance and Use of Technology	GP	EP	HPP
Liu et al.^16^	China	292	Value Co-Creation	PA	EP	DMRC
Akter et al.^59^	Bangladesh	473	Cognition–Affective–Conation	GP	EP	HPP
Soni et al.^60^	India	263	Self-Determination Theory	GP	YI	HPP
Song et al.^61^	China	129	Information System Success Model; Expectation Confirmation Model of IS Continuance	PA	EP	HPP
Huang and Ren^62^	China	449	Technology Acceptance Model	GP	EP	HPP
Yuan et al.^63^	The United States	317	Technology Acceptance Model; the Unified Theory of Acceptance and Use of Technology	GP	YI	HPP
Grenier Ouimet et al.^19^	Canada	178	—	PA	EP	DMRC
Tsai et al.^64^	Taiwan, China	60	the Theory of Reasoned Action	GP	OI	HPP
Liu et al.^29^	China	323	Uses and Gratifications theory; Stimulus-Organism-Response Model	PA	EP	DMRC
Kim et al.^65^	South Korea	191	SERVQUAL	GP	YI	HPP
Akter et al.^30^	Bangladesh	210	Value Co-Creation; Consumer Culture Theory; service-dominant logic	PA	EP	DMRC
Lee and Lee^66^	South Korea	129;159	Theory of Planned Behavior; Knowledge, Attitudes, and Practices Model; Health Belief Model; the Unified Theory of Acceptance and Use of Technology	HCP; GP	EP	WD
Guo et al.^67^	China	255	Elaboration Likelihood Model	PA	EP	DMRC
Beldad and Hegner^68^	German	476	Technology Acceptance Model	GP	YI	HPP
Meng et al.^69^	China	261	the Trust Theory	GP	OI	HPP
Luo et al.^26^	China	368	Protection Motivation Theory	GP	EP	DMRC
Hong et al.^70^	China	283	—	GP	EP	HPP
Leung and Chen^71^	Hong Kong, China	387	Expectation Confirmation Model of IS Continuance	GP	EP	HPP
Paré et al.^20^	Canada	580	Expectation Confirmation Model of IS Continuance; Technology Acceptance Model	GP	EP	WD
Akter et al.^72^	Bangladesh	283	SERVQUAL; Information System Success Model	GP	EP	DMRC
Birkmeyer et al.^73^	German	249	Technology Acceptance Model	GP	EP	HPP
Kim and Han^32^	South Korea	250	Social Cognitive Theory	GP	OI	HPP
Hartono et al.^23^	Indonesia	101	the Unified Theory of Acceptance and Use of Technology; Perceived Technology Security	GP	EP	DMRC
Kim et al.^74^	The United States	134	the Unified Theory of Acceptance and Use of Technology	PA	OI	DMRC
Zhang and Xu^75^	China	379	Expectation Confirmation Model of IS Continuance; Uses and Gratifications theory;	GP	YI	HPP
Chiu et al.^76^	China	342	Expectation Confirmation Model of IS Continuance; Investment Model	GP	EP	HPP
Jaana and Paré^77^	Canada	384	Expectation Confirmation Model of IS Continuance	GP	OI	WD
Chen et al.^78^	China	284	Elaboration Likelihood Model	GP	EP	DMRC
Chen et al.^79^	Taiwan, China	313	Technology Readiness	GP	EP	DMRC
Hossain^80^	Bangladesh	199	Information System Success Model	GP	EP	DMRC
Chen et al.^81^	China	284	Equity Theory; Stimulus-Organism-Response Model	PA	EP	DMRC
Anil Kumar and Natarajan^82^	India	453	Expectation Confirmation Model of IS Continuance; Technology Acceptance Model	PA	EP	DMRC
Hsieh et al.^83^	Taiwan, China	90	Expectation Confirmation Model of IS Continuance	HCP	EP	DMRC
Choi and Lee^84^	Australia	50	Expectation Confirmation Model of IS Continuance; Organismic Integration Theory	GP	EP	HPP

*YI* young individuals, *OI* older individuals, *EP* entire population, *HPP* health promotion and prevention, *DMRC* disease management and remote consultation, *WD* wearable devices, *GP* general public, *PA* patient, *HCP* health care professional.

Notes: 1. Young individuals are those under the age of 35, while older individuals are those who are over the age of 60. 2. mHealth for health promotion and prevention target determinants of health to promote health and prevent health problems; mHealth for disease management and remote consultation are used frequently to help patients (and healthcare professionals) manage diseases, particularly long-term conditions such as diabetes, chronic respiratory conditions, cancer, and mental health problems, or conduct remote disease consultation; medical wearable medical devices are any non-invasive device that has the ability to collect, transmit, and visualize patient health data.

### Meta-analysis

**Fig. 2:**
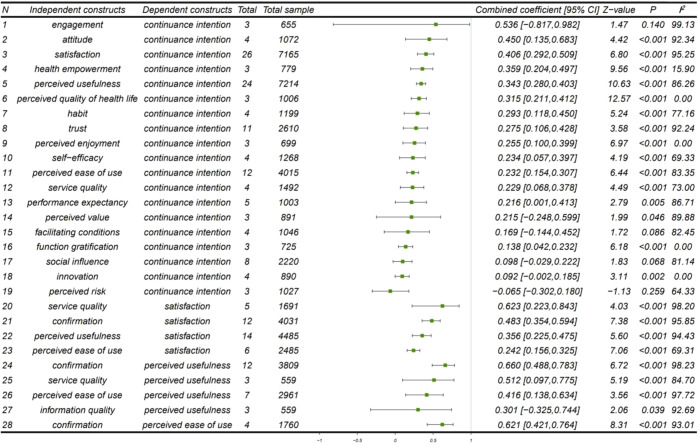
Summary estimation of pooled effect of coefficients using forest plot. Data presented forest plot of influence relationships included in the meta-analysis (28 influence relationships). *P*: *p*-value for the combined coefficient significance test of the effect of the independent constructs on the dependent constructs.

**Fig. 3:**
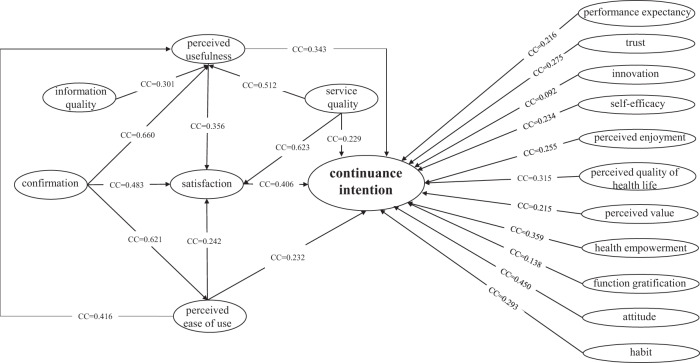
Path diagram of the influence relationship between variables. Drawing a path diagram for all the influence relationships with significant pooled effects, including 15 direct and 9 indirect influence relationships on continuance intention. Notes: CC = Combined coefficient.

(1) Direct impact factors of continuance intention. Figure 2 shows that out of the 19 direct impact factors of continuance intention, the pooled effects of four factors were not significant (social influence (0.098 [95% CI, −0.029, 0.222], *P* = 0.068, I^2^ = 81.14%); facilitating conditions (0.169 [95% CI, −0.144, 0.452], *P* = 0.086, *I*^2^ = 82.45%); perceived risk (−0.065 [95% CI, −0.302, 0.180], *P* = 0.259, *I*^2^ = 64.33%); engagement (0.536 [95% CI, −0.817, 0.982], *P* = 0.140, *I*^2^ = 99.13%)). The five factors that have been explored the most were satisfaction (26 times), perceived usefulness (24 times), perceived ease of use (12 times), trust (11 times), and social influence (8 times). According to the size of the pooled effect coefficients, the top five factors that have a direct impact on continuance intention were the attitude (0.450 [95% CI, 0.135, 0.683], *P* < 0.001, *I*^2^ = 92.34%), satisfaction (0.406 [95% CI, 0.292, 0.509], *P* < 0.001, *I*^2^ = 95.25%), health empowerment (0.359 [95% CI, 0.204, 0.497], *P* < 0.001, *I*^2^ = 15.90%), perceived usefulness (0.343 [95% CI, 0.280, 0.403], *P* < 0.001, *I*^2^ = 86.26%), and perceived quality of health life (0.315 [95% CI, 0.211, 0.412], *P* < 0.001, *I*^2^ = 0.00%), which were regarded as main impact factors on continuance intention.

(2) Impact factors of mediating factors. We also integrated and analyzed the impact factors of satisfaction, perceived usefulness, and perceived ease of use to explore the factors that indirectly affect the continuance intention. All four independent variables of satisfaction were significant; the most explored factor was perceived usefulness (14 times), and the pooled effect coefficient of service quality was the largest (0.623 [95% CI, 0.223, 0.843], *P* < 0.001, *I*^2^ = 98.20%). The pooled effects of the four independent variables of perceived usefulness were all significant, confirmation was the most explored impact factor (12 times), and the pooled effect coefficient of confirmation was the largest (0.660 [95% CI, 0.488, 0.783], *P* < 0.001, *I*^2^ = 98.23%). The pooled effect of confirmation on perceived ease of use was significant. The effect estimates and confidence intervals for both individual studies and the overall combined results were illustrated using a forest plot, which are shown in the Supplementary Figs. A1–A28 and Supplementary Table A1–A28. Figure 2 shows that most studies are highly heterogeneous. A path diagram was drawn for all the influence relationships with significant pooled effects in Fig. 3.

### Subgroup analysis

The results of our subgroup analyses stratified by the study design and population are summarized in Tables 2–6. Table 2 revealed that the association between trust and continuance intention, social influence and continuance intention, confirmation and satisfaction, and perceived ease of use and perceived usefulness were substantially changed by geographic region. The impact effects were stronger in the developing countries or regions than in the developed countries or regions.

**Table 2:** Subgroup analysis by country or region.

N	Independent constructs	Dependent constructs	Sum of squares	*P*^a^	Subgroup	Total	Total sample	Combined coefficient	*P*^b^	95% confidence interval (low to high)	*I*^2^
1	Satisfaction	Continuance intention	0.008	0.927	A	14	3150	0.410	<0.001	0.240 to 0.556	93.84
					B	12	4015	0.401	<0.001	0.214 to 0.559	97.59
2	Perceived usefulness	Continuance intention	0.013	0.909	A	13	3662	0.349	<0.001	0.233 to 0.455	90.79
					B	11	3552	0.342	<0.001	0.280 to 0.402	69.19
3	Perceived ease of use	Continuance intention	0.577	0.477	A	7	2165	0.257	<0.001	0.143 to 0.364	82.13
					B	5	1850	0.199	<0.001	0.041 to 0.348	84.07
4	Trust	Continuance intention	1.876	0.171	A	4	671	0.121	0.477	−0.398 to 0.581	93.67
					B	7	1939	0.353	<0.001	0.200 to 0.489	90.13
5	Social influence	Continuance intention	2.844	0.092	A	5	1443	0.057	0.472	−0.162 to 0.271	81.87
					B	3	777	0.182	<0.001	0.045 to 0.311	0.00
6	Confirmation	Satisfaction	6.332	0.012	A	7	2035	0.365	<0.001	0.281 to 0.443	60.29
					B	5	1996	0.606	<0.001	0.336 to 0.784	96.55
7	Perceived usefulness	Satisfaction	2.793	0.095	A	7	2035	0.261	<0.001	0.180 to 0.337	52.78
					B	7	2450	0.439	<0.001	0.175 to 0.644	96.92
8	Perceived ease of use	Satisfaction	0.076	0.783	A	3	1307	0.252	<0.001	0.016 to 0.461	75.99
					B	3	1178	0.231	<0.001	0.007 to 0.433	70.98
9	Confirmation	Perceived usefulness	0.001	0.976	A	8	2213	0.662	<0.001	0.460 to 0.799	97.19
					B	4	1596	0.657	0.005	−0.101 to 0.932	99.17
10	Perceived ease of use	Perceived usefulness	46.659	<0.001	A	4	1783	0.183	<0.001	0.083 to 0.280	41.56
					B	3	1178	0.653	<0.001	0.413 to 0.808	86.93

Notes: A: developed countries, B: developing countries. All *P* values were two-sided with a significance level of 0.05. a: *P*-value of test for groups difference of combined coefficient. b: *P*-value of test for significance of combined coefficient.

**Table 3:** Subgroup analysis by user type.

N	Independent constructs	Dependent constructs	Sum of squares	*P*^a^	Subgroup	Total	Total sample	Combined coefficient	*P*^b^	95% confidence interval (low to high)	*I*^2^
1	Satisfaction	Continuance intention	0.450	0.502	A	18	5496	0.442	<0.001	0.321 to 0.550	96.27
					B	6	1378	0.346	0.054	−0.120 to 0.687	96.47
2	Trust	Continuance intention	1.521	0.217	A	6	1359	0.351	<0.001	0.257 to 0.440	55.18
					B	5	1251	0.172	0.286	−0.271 to 0.554	96.24
3	Perceived usefulness	Continuance intention	1.571	0.210	A	15	5264	0.320	<0.001	0.245 to 0.391	86.82
					B	7	1659	0.405	<0.001	0.257 to 0.534	84.92
4	Perceived ease of use	Continuance intention	3.591	0.058	A	8	3157	0.255	<0.001	0.143 to 0.361	86.57
					B	4	858	0.146	<0.001	0.050 to 0.240	0.00
5	Social influence	Continuance intention	17.152	<0.001	A	5	1410	−0.012	0.515	−0.065 to 0.040	0.00
					B	3	810	0.248	<0.001	−0.012 to 0.477	57.77
6	Perceived usefulness	Satisfaction	0.039	0.844	A	9	3118	0.358	<0.001	0.171 to 0.520	95.76
					B	3	1076	0.386	0.031	−0.386 to 0.840	94.81
7	Confirmation	Perceived usefulness	6.718	0.010	A	7	2393	0.711	<0.001	0.455 to 0.859	98.20
					B	3	1125	0.387	<0.001	−0.084 to 0.716	90.33

Notes: A: general public, B: patients. All *P* values were two-sided with a significance level of 0.05. a: *P*-value of test for groups difference of combined coefficient. b: *P*-value of test for significance of combined coefficient.

**Table 4:** Subgroup analysis by mHealth type.

N	Independent constructs	Dependent constructs	Sum of squares	*P*^a^	Subgroup	Total	Total sample	Combined coefficient	*P*^b^	95% confidence interval (low to high)	*I*^2^
1	Satisfaction	Continuance intention	0.116	0.733	A	14	3784	0.420	<0.001	0.248 to 0.566	97.16
					B	10	2417	0.381	<0.001	0.148 to 0.574	95.17
2	Trust	Continuance intention	2.719	0.099	A	3	616	0.403	<0.001	0.137 to 0.614	67.50
					B	8	1994	0.225	0.023	−0.009 to 0.436	93.70
3	Perceived usefulness	Continuance intention	10.142	0.006	A	13	4200	0.298	<0.001	0.217 to 0.375	84.78
					B	8	1827	0.457	<0.001	0.364 to 0.541	77.54
					C	3	1187	0.237	0.001	−0.080 to 0.511	85.27
4	Perceived ease of use	Continuance intention	2.497	0.287	A	6	2193	0.262	<0.001	0.109 to 0.402	87.87
					B	3	635	0.147	0.001	−0.047 to 0.331	12.25
					C	3	1187	0.214	0.001	−0.070 to 0.467	83.10
5	Social influence	Continuance intention	7.751	0.005	A	4	1309	−0.019	0.269	−0.074 to 0.036	0.00
					B	3	688	0.218	0.014	−0.165 to 0.543	70.43
6	Perceived usefulness	Satisfaction	0.063	0.802	A	8	2355	0.381	<0.001	0.170 to 0.559	96.04
					B	4	1166	0.347	0.012	−0.094 to 0.674	92.33
7	Confirmation	Perceived usefulness	0.984	0.321	A	6	1630	0.672	<0.001	0.375 to 0.843	97.77
					B	4	1215	0.528	0.003	−0.048 to 0.840	95.86

Notes: A: health promotion and prevention, B: disease management and remote consultation, C: wearable devices. All *P* values were two-sided with a significance level of 0.05. a: *P*-value of test for groups difference of combined coefficient. b: *P*-value of test for significance of combined coefficient.

**Table 5:** Subgroup analysis by participants’ age.

N	Independent constructs	Dependent constructs	Sum of squares	*P*^a^	Subgroup	Total	Total sample	Combined coefficient	*P*^b^	95% confidence interval (low to high)	*I*^2^
1	Satisfaction	Continuance intention	13.150	0.001	A	19	5358	0.284	<0.001	0.221 to 0.345	82.49
					B	4	964	0.761	<0.001	0.352 to 0.926	97.91
					C	3	843	0.521	<0.001	0.091 to 0.788	91.15
2	Perceived usefulness	Continuance intention	0.001	0.980	A	19	5606	0.350	<0.001	0.271 to 0.424	88.74
					C	3	1001	0.349	<0.001	0.276 to 0.417	0.00

Notes: A: entire population, B: older individuals, C: young individuals. All *P* values were two-sided with a significance level of 0.05. a: *P*-value of test for groups difference of combined coefficient. b: *P*-value of test for significance of combined coefficient.

**Table 6:** Subgroup analysis by publication year.

N	Independent constructs	Dependent constructs	Sum of squares	*P*^a^	Subgroup	Total	Total sample	Combined coefficient	*P*^b^	95% confidence interval (low to high)	*I*^2^
1	Satisfaction	Continuance intention	0.599	0.439	A	9	1764	0.460	<0.001	0.212 to 0.652	93.92
					B	17	5401	0.378	<0.001	0.236 to 0.503	96.98
2	Perceived usefulness	Continuance intention	0.043	0.837	A	4	432	0.363	<0.001	0.055 to 0.608	81.20
					B	20	6782	0.341	<0.001	0.272 to 0.406	87.45
3	Trust	Continuance intention	0.274	0.600	A	4	709	0.220	0.170	−0.288 to 0.632	92.30
					B	7	1901	0.304	0.001	0.090 to 0.491	88.74
4	Perceived ease of use	Continuance intention	1.047	0.306	A	3	382	0.174	<0.001	−0.004 to 0.341	0.00
					B	9	3633	0.242	<0.001	0.140 to 0.339	87.18

Notes: A: 2011–2015, B: 2016-2021. All *P* values were two-sided with a significance level of 0.05. a: *P*-value of test for groups difference of combined coefficient. b: *P*-value of test for significance of combined coefficient.

The association between satisfaction and continuance intention, trust and continuance intention, social influence and continuance intention, and confirmation and perceived usefulness substantially changed by the user type (Table 3). Compared with public users, the impact of satisfaction and trust on the continuance intention was not significant in the patients’ group. Compared with the patients’ group, the impact of social influence on the continuance intention was not significant in the public group. The impact effect of confirmation on perceived usefulness was stronger in the public users’ group than in the patient group.

The associations between perceived usefulness and continuance intention, social influence and continuance intention were substantially changed by the mHealth type (Table 4). Compared with mHealth dedicated to disease management and remote consultation, the impact of social influence on the continuance intention was not significant in mHealth dedicated to health promotion and prevention. The impact effect coefficient of perceived usefulness on continuance intention was largest in the group of mHealth apps dedicated to disease management and remote consultation.

The association between satisfaction and continuance intention was substantially changed by the age of the study population (Table 5). The impact effect coefficient of satisfaction on continuance intention was the largest among older individuals.

The association between trust and continuance intention was substantially changed by the publication year (Table 6). The impact of trust on continuance intention was only significant in recent years (2016–2021). However, the results were not substantially changed by the study quality (Supplementary Table 2).

### Publication bias

Funnel plot and Egger linear regression tests revealed little evidence of publication bias (Supplementary Fig B1–B28).

## Discussion

Ranked according to the size of pooled effect coefficients, the top five factors that affect the continuance intention of mHealth were attitude, satisfaction, health empowerment, perceived usefulness, and perceived quality of health life. Attitude towards mHealth refers to a user’s positive or negative sentiment towards a mHealth service or device.^26^ Individuals’ cognitive coordination between seeking perception and behavior leads to attitudes toward specific behaviors that affect continuance intention.^27^ However, users have different attitudes towards the same mHealth service, and exploring ways to improve user attitudes should be studied in the future. User satisfaction refers to the user’s overall evaluation of product performance.^28^ Users evaluate satisfaction by comparing the actual service or product with their expectations of the service or product before use.^22^ High satisfaction means that the product or service performance exceeds user expectations; thus, they are more likely to continue using them. Satisfaction is an important precursor variable of the continuance intention in many theories and models, such as ECM-ISC and TAM, and is also the most frequently explored factor in studies of continuance intention of mHealth. This indicates that the positive impact of satisfaction on continuance intention is widely recognized. Health empowerment is an effective strategy for promoting personal health, and self-management is an important aspect of health empowerment.^29^ Specifically, health empowerment is the realization of patients’ control of their health-related conditions and awareness of the resources available in the healthcare service system through mHealth.^30^ Compared to traditional offline health management and disease control methods, mHealth has the advantages of reduced costs, time efficiency and more accessibility. In the use of mHealth services, if patients feel that they can effectively manage their health and control their health status, this will increase the possibility of mHealth continuance and achieve savings in terms of cost and time. Perceived usefulness refers to the degree to which individuals believe that using a specific system will improve their work performance.^28^ mHealth is a tool or service with an evident utilitarian nature that can be used by users to seek health management services or carry out disease prevention and control. Whether mHealth can improve the effect and efficiency of its health management is the focus of the user’s concern; therefore, high perceived usefulness is an important factor that promotes the continuance intention of users. Perceived quality of health life refers to the results obtained by users through their interactions with the mHealth, and could be defined as a sense of overall wellbeing in health.^31^ Continuous self-health management behavior is not easy, and users need a regulatory mechanism that includes goal setting, monitoring, and feedback reception.^32^ Perceived quality of health life is a kind of feedback reception of health management behavior for users. Positive result feedback will give users encouragement to continue setting goals. When this regulatory mechanism works well, users will spontaneously carry out continuous health management behaviors. Therefore, perceived quality of health life is an important factor in promoting users’ continuance intention.

In summary, among the five factors with the largest size pooled effect on mHealth continuance intention, two factors were related to mHealth functions and service quality (the degree to which the mHealth serves its actual purpose and to what extent improvement in the efficiency of user health management has a positive impact on continuance intention) and two factors were related to the user’s comprehensive feelings about mHealth (the user’s comprehensive emotion and overall evaluation of the impact of mHealth use on continuance intention). Lastly, health empowerment, which is closely related to health management, has a significant impact on the continuance intention of mHealth. This is also a unique impact factor on the user’s behavior in the field of mHealth, which was not mentioned in the review of the continuance intention of information systems and online technologies,^25,33^ representing an important finding of our study.

A meta-analysis can clarify whether a certain impact factor has a significant influence on continuance intention, while a subgroup analysis can further explore whether a specific impact factor has a stronger influence on a certain type of research subject and find significant differences in the effect of the same factor after adjustment of different characteristics. We found that the economic development level of countries or regions has a significant moderating effect on impact. Compared with developed countries or regions, confirmation in developing countries or regions has a stronger effect on satisfaction ((0.606 [95% CI, 0.336, 0.784], *P* < 0.001, *I*^2^ = 96.55%) vs. (0.365 [95% CI, 0.281, 0.443], *P* < 0.001, *I*^2^ = 60.29%)), indicating that users in developing countries consider whether the performance of the mHealth has reached the expected level when deciding on whether to continue using it. Similarly, in developing countries or regions, the impact effect of perceived ease of use on perceived usefulness was strengthened ((0.653 [95% CI, 0.413, 0.808], *P* < 0.001, *I*^2^ = 86.93%) vs. (0.183 [95% CI, 0.083, 0.280], P < 0.001, I^2^ = 41.56%)). This indicates that users in developing countries or regions are more inclined to consider mHealth to be useful if they think that it is easy to use, compared to those in developed countries or regions, who believe that the perceived ease of use of mHealth has a relatively weak effect on its perceived usefulness. In addition, the impact of trust on continuance intention has a significant effect only in developing countries or regions ((0.353 [95% CI, 0.200, 0.489], *P* < 0.001, *I*^2^ = 90.13%) vs. (0.121 [95% CI, −0.398, 0.581], *P* = 0.477, *I*^2^ = 93.67%)), which may be attributed to the rapid development of science and technology in economically developed countries and their tendency to use more advanced technologies.^34^ However, mHealth technology in developing countries is still in its early stages of development, and users are still in the initial stage of accepting mHealth services and have more uncertainty about it. We hypothesize that once users trust mHealth services, they will be more inclined to continue using them.

The user type has a significant moderating effect on impact effects. The impact effect of satisfaction on continuance intention was only significant in the public ((0.442 [95% CI, 0.321, 0.550], P < 0.001, I^2^ = 96.27%) vs. (0.346 [95% CI, −0.120, 0.687], P = 0.054, I^2^ = 96.47%)), indicating that the public is more inclined to continue using mHealth when they have a higher satisfaction. The impact effect of confirmation on perceived usefulness was significantly enhanced in the public user group ((0.711 [95% CI, 0.455, 0.859], P < 0.001, I^2^ = 98.20%) vs. (0.387 [95% CI, −0.084, 0.716], P < 0.001, I^2^ = 90.33%)), indicating that when their expectations were confirmed, they were more inclined to think that mHealth was useful. Compared with patients who need disease management, the expectations of healthy people were relatively easy to be confirmed. In contrast, the impact of social influence on continuance intention was only significant in patients than healthy individuals ((0.248 [95% CI, −0.012, 0.477], P < 0.001, I^2^ = 57.77%) vs. (−0.012 [95% CI, −0.065, 0.040], P = 0.515, I^2^ = 0.00%)), indicating that if the social environment of the patient supports the use of mHealth technology, then patients are more inclined to continue using mHealth. The difference in impact effects between the two types of users is related to their purpose in using mHealth services and the types of services and tools; therefore, developers of mHealth technology should target their users accordingly.

The mHealth type has a significant moderating effect on impact effects. The impacts of perceived usefulness and social influence on continuance intention were significantly enhanced in mHealth targeted toward disease management and remote consultation. This phenomenon is related to the fact that the target users of this kind of mHealth are patients, and their main purpose when using it is the remote consultation or disease management. When the mHealth meets its utilitarian needs, users find it useful and are more likely to continue using it.

User age has a significant moderating effect on the impact effects. The pooled effect coefficient of satisfaction on continuance intention was the largest in older adults (0.761 [95% CI, 0.352, 0.926], *P* < 0.001, *I*^2^ = 97.91%) and the smallest in the entire age group (0.284 [95% CI, 0.221, 0.345], *P* < 0.001, *I*^2^ = 82.49%), indicating that when users are satisfied with mHealth, older users have a greater tendency to continue using it than other age groups. These results provide theoretical bases for establishing personalized mHealth designs. mHealth services can be designed to provide various functions of personalized experience according to the age of users, such as focusing on exploring the impact that affects the satisfaction of older adult users.

Analysis of the moderating effect of the publication year revealed the changing trends of the impact factors. The impact of trust on continuance intention was not significant in studies published earlier but showed a significant effect in studies published more recently ((0.220 [95% CI, −0.288, 0.632], *P* = 0.170, *I*^2^ = 92.30%) vs. (0.304 [95% CI, 0.090, 0.491], *P* = 0.001, *I*^2^ = 88.74%)). This finding can be attributed to the increased trust of users in mHealth services. With technological advances, users are gradually becoming concerned about the security issues associated with technology.

According to the research results, the factors with the strongest pooled impact effect on mHealth continuance intention can be divided into three aspects: mHealth function and service quality, users’ comprehensive feelings towards mHealth and health empowerment. Therefore, mHealth developers can improve the design in three ways. First, efforts should be made to improve the effectiveness of mHealth in health promotion and disease management. For example, the health interventions provided should be verified by evidence-based medicine and significantly improve the health status of users. Users’ perception that mHealth is useful for their health management is an important basis for continued use. Second, developers can collect users’ evaluation of various functions in their experience with the application through user generated content, solve the limitations of functions, and improve user satisfaction.^35^ Finally, health empowerment has a significant positive impact on continuance intention, indicating that users not only require mHealth to significantly improve their health status, but also hope that mHealth can improve their health management ability. Therefore, mHealth should provide enough health education to improve users’ health literacy, so that users can make health management decisions and fully participate in their own health management.

Subgroup analyses showed that the geographic region, user type and mHealth type significantly affected the results. Therefore, developers should clarify the target population of mobile health and distinguish the countries and regions of its distribution. First, trust had a significant impact on continuance intention only in developing countries, and the impact of perceived ease of use on perceived usefulness increased significantly in developing countries. This result may be due to the immature development of mHealth technology in developing countries and the consequent uncertainty among users. Developers should strengthen the usability of mHealth in developing countries, simplify the process of human-computer interaction, fully display the design and role of mHealth, improve its security, and increase users’ trust and perceived ease of use. Second, developers should pay attention to the differences between user types and mHealth types. The impact of social influence on continuance intention is only significant in the patients’ population and mHealth for disease management and remote consultation. Therefore, mHealth for patients should take measures to increase the social influence of products. For example, mHealth can provide an online community for exchange between patients and increase users’ opportunities to accept social influence. In contrast, operators can consider promoting mHealth among medical professionals. After recognition, medical professionals may support their patients to continue using mHealth to improve their health status.

This study had some limitations. First, although we searched the literature as comprehensively as possible, several studies were not retrieved owing to the limitations of the literature database. Nevertheless, we searched eight databases from multidisciplinary focus areas and developed a retrieval strategy considering that search terms have multiple meanings. Therefore, we consider our study to be representative. Second, although this study verified the moderating effects of the economic development level of countries or regions, user type, mHealth type, user age, and publication time on the impact effects, characteristics that have moderating effects, such as cultural characteristics, income, gender, health literacy, race, and user occupations, may have been unidentified. Owing to the lack of comprehensive data in the included literature, we did not analyze the role of the above-mentioned factors; thus, follow-up studies should explore these characteristics.

The continuance of mHealth plays an important role in building a self-health management model that realizes user health empowerment and is also the key to the survival of mHealth companies in the market. A comprehensive evaluation of the impact factors and moderating factors of mHealth continuance intention is of great value for proposing improvement strategies. To summarize, this study analyzed 58 quantitative studies on the continuance intention of mHealth services, and explored the pooled effect of 28 impact effects through meta-analysis. The results showed that the theoretical basis of the current mHealth continuance intention studies tend to be integrated and diversified; ECM-ISC, TAM, and UTAUT are the widely used theoretical models, of which ECM-ISC is the most widely recognized. Moreover, ranked according to the size of pooled effect coefficients, the top five factors that affect the continuance intention of mHealth were the attitude, satisfaction, health empowerment, perceived usefulness, and perceived quality of health life. Health empowerment is a specific factor of the user’s information behavior in the health field and may attract the attention of scholars and developers in the future. The results of subgroup analysis showed that the geographic region, user type, mHealth type, user age and research publication time have a significant moderating effect on some impact effects. Therefore, researchers and developers should consider the countries or regions where mHealth is used, accurately identify the user characteristics of the target users, and design personalized mHealth services to improve the continuance of mHealth and ultimately achieve a sustainable self-health management model.

## Methods

### Search strategy

We followed the Preferred Reporting Items for Systematic Reviews and Meta-Analyses (PRISMA) guidelines for conducting and reporting items for systematic reviews (Supplementary Table 3). A systematic literature search for cross-sectional studies up to October 8, 2021, was conducted in the following databases: PubMed, Embase, WOS core collection, CINAHL, Scopus, PsycInfo, EI and ACM. The retrieval formula consisted of three parts: “mHealth”, “continuance”, and “intention”. The search had no language restriction. We also searched and reviewed the references cited within the retrieved relevant reports for any additional studies. The detailed retrieval formula is shown in Supplementary Table 4.

### Study selection

Studies were included in the current meta-analysis if they met the following criteria: (1) The full text could be obtained. For articles of which the full text was not provided by the literature database and publishers, we contacted the author of the article through ResearchGate (a scientific research social network service website) to request it. If by the end of the data analysis there was no response to the full-text request, it was regarded as being unable to obtain the full-text. (2) The main outcome of the study was the continuance intention. Continuance intention is the user’s willingness and tendency to continue using mHealth.^15^ This variable was measured by a self-reported questionnaire, usually on a 5 or 7 point Likert scale, such as “I intend to continue using the mHealth App I used rather than discontinue its use”. (3) The type of study was quantitative. (4) The research object was mHealth related products or services. WHO defined mHealth as “medical and public health practice supported by mobile devices, such as mobile phones, patient monitoring devices, PDAs, and other wireless devices”.^1^ Therefore, the mHealth selected in this study needed to meet two characteristics: the products or services should be provided through mobile devices and the service provided by mHealth belonged to the scope of medical or health care. The participants of the study were users who had used mHealth, including the general population for preventive care and patients for disease management. The exclusion criteria were as follows: (1) studies not showing regression coefficients between the variables (research methods were not based on correlation analysis or regression analysis); (2) reviews, letters, comments, editorial; and (3) studies not published under a peer-review process. Three researchers (Tong Wang, Jun Liang, and Mingfu Nuo) independently screened titles and abstracts and the full text of each potentially relevant article was then evaluated.

### Data extraction and quality assessment

A standardized data collection form was used to extract data. From each included study, we extracted the following information: first author, publication year, paper title, mHealth type, user type and age characteristics, sample size, country or region where the study was conducted, statistical methods, independent and dependent variables, regression coefficient, *P* value and other statistical indices. In some of the included studies, the hypothesis of the association is based on the relevant theories of psychology or behavior, therefore, we collected the theories or models on which the hypothesis is based. Two independent investigators (Tong Wang and Mingfu Nuo) performed the data extraction process, and any disagreements were resolved with group discussion.

The quality of included studies was evaluated according to the JBI PACES checklist for analytical cross-sectional studies^36^ by two researchers (Tong Wang and Jun Liang).

### Statistical analysis

In this meta-analysis, the main outcome was the pooled regression coefficient with 95% confidence intervals. According to Sarkar et al.,^37^ the influence relationships included in the meta-analysis must be tested at least three times in different studies. Statistical analysis was performed on the associations between the impact factors and continuance intention that were tested at least three times in the included studies. Path analysis was usually used in the study of continuance intention to explore the direct and indirect effects of impact factors on continuance intention. Therefore, we not only integrated the direct impact factors of continuance intention, but also integrated the impact factors of its main direct impact factors to analyze the variables that have indirect effects. Taking the three most relevant impact factors of continuance intention as mediating factors, we analyzed the integration effect of the association between the impact factors and mediating factors. We calculated the summarized regression coefficients and their corresponding 95% CI using a random-effects model^24^, which could incorporate both within- and between-study variability. Additionally, we applied the inverse variance weighting method with an additive between-studies variance component in the random effects model based on the DerSimonian-Laird estimator to pool the effect sizes. Owing to many variables included in the studies, with most of them being users’ subjective feelings, the definitions and measurements of all variables in each study were distinct. We grouped the same construct from the same theory or model as the same variable. For the same constructs derived from different theories or no theoretical basis, researchers differentiated them according to definition and measurement items. Taking the definition with more occurrences as the standard, the concepts that are different from the standard definition were regarded as different variables and were not integrated. Any disagreements were solved by group discussion.

The included studies were analyzed for bias risk using funnel plots and Egger’s tests. Heterogeneity between studies was assessed using I^2^ ( <50%, 50–74%, and >75% were low, moderate and high heterogeneity, respectively).

To examine the significance of the difference in regression coefficients and the possible influence of residual confounding factors, we performed subgroup analyses on possible sources of heterogeneity, including geographic location, user’s type, mHealth type, user’s age, and study quality. First, some studies have shown that the economic development of a country or region has a moderating effect on the use of information systems;^25^ therefore, we classified the studies into economically developed and developing groups according to the countries or regions where the survey was conducted. Second, Chauhan et al. found that the types of users and eHealth app have a significant moderating effect on their acceptance. Based on their classification of the user types, we categorized the studies into the general public and patient groups (subgroup analyses of healthcare professionals were not performed due to the small number of studies using healthcare professionals as mHealth users).^24^ Users of mHealth were mainly divided into healthy individuals and patients with diseases.^24^ According to different target users, mHealth can be divided into two categories: 1. health promotion and prevention, and 2. disease management and remote consultation.^24,38^ In addition, according to the main functions of the device, wearable devices for health status monitoring were an important category of mHealth. Therefore, mHealth was divided into three subgroups in this study: 1. health promotion and prevention, 2. disease management and remote consultation, and 3. wearable devices. Finally, some studies categorized the study participants as college students^39^ or older adults aged ≥60 years^32^; therefore, we analyzed the age characteristics of the participants and categorized the studies as follows: young adults (18–35 years old), older adults (≥60 years old), and the entire age group. In addition, the studies were divided into two groups according to publication year: early (2010–2015) and recent (2016–2021).

We used the SPSS statistical software version 24 and meta-essentials 1.1 for all statistical analyses^40^, and all P values were two-sided with a significance level of 0.05. This study was not registered.

### Reporting summary

Further information on research design is available in the Nature Research Reporting Summary linked to this article.

## Supplementary information

Supplementary Information

Reporting Summary

## Data Availability

The authors declare that all the data included in this study are available within the paper and its Supplementary Information files.
