# Supplementary material for: Identifying major impact factors affecting the continuance intention of mHealth: a systematic review and multi-subgroup meta-analysis
Source: NPJ Digit Med. 2022 Sep 15;5:145. doi: 10.1038/s41746-022-00692-9 (PMC9476418; doi:10.1038/s41746-022-00692-9)
Supplement: Supplementary file 1 — Supplementary Information [file 41746_2022_692_MOESM1_ESM.pdf]

Supplementary Information

Identifying major impact factors affecting the continuance intention of mHealth: systematic review and multi-subgroup meta-analysis

Supplementary Table 1. Quality assessment of included prospective studies 1
Supplementary Figure A1. Forest plot of the random-effects meta-analysis of the association between satisfaction and continuance intention 7
Supplementary Table A1. Pooled regression coefficient of the association between satisfaction and continuance intention 8
Supplementary Figure A2. Forest plot of the random-effects meta-analysis of the association between perceived usefulness and continuance intention 9
Supplementary Table A2. Pooled regression coefficient of the association between perceived usefulness and continuance intention 10
Supplementary Figure A3. Forest plot of the random-effects meta-analysis of the association between perceived ease of use and continuance intention 11
Supplementary Table A3. Pooled regression coefficient of the association between perceived ease of use and continuance intention 11
Supplementary Figure A4. Forest plot of the random-effects meta-analysis of the association between trust and continuance intention 12
Supplementary Table A4. Pooled regression coefficient of the association between trust and continuance intention 12
Supplementary Figure A5. Forest plot of the random-effects meta-analysis of the association between social influence and continuance intention 13
Supplementary Table A5. Pooled regression coefficient of the association between social influence and continuance intention 13
Supplementary Figure A6. Forest plot of the random-effects meta-analysis of the association between performance expectancy and continuance intention 14
Supplementary Table A6. Pooled regression coefficient of the association between performance expectancy and continuance intention 14
Supplementary Figure A7. Forest plot of the random-effects meta-analysis of the association between facilitating conditions and continuance intention 15
Supplementary Table A7. Pooled regression coefficient of the association between facilitating conditions and continuance intention 15
Supplementary Figure A8. Forest plot of the random-effects meta-analysis of the association between service quality and continuance intention 15
Supplementary Table A8. Pooled regression coefficient of the association between service quality and continuance intention 15

Supplementary Figure A9. Forest plot of the random-effects meta-analysis of the association between attitude and continuance intention 16
Supplementary Table A9. Pooled regression coefficient of the association between attitude and continuance intention 16
Supplementary Figure A10. Forest plot of the random-effects meta-analysis of the association between habit and continuance intention 16
Supplementary Table A10. Pooled regression coefficient of the association between habit and continuance intention 16
Supplementary Figure A11. Forest plot of the random-effects meta-analysis of the association between innovation and continuance intention 17
Supplementary Table A11. Pooled regression coefficient of the association between innovation and continuance intention 17
Supplementary Figure A12. Forest plot of the random-effects meta-analysis of the association between self-efficacy and continuance intention 17
Supplementary Table A12. Pooled regression coefficient of the association between self-efficacy and continuance intention 17
Supplementary Figure A13. Forest plot of the random-effects meta-analysis of the association between perceived enjoyment and continuance intention 18
Supplementary Table A13. Pooled regression coefficient of the association between perceived enjoyment and continuance intention 18
Supplementary Figure A14. Forest plot of the random-effects meta-analysis of the association between perceived quality of health life and continuance intention 18
Supplementary Table A14. Pooled regression coefficient of the association between perceived quality of health life and continuance intention 18
Supplementary Figure A15. Forest plot of the random-effects meta-analysis of the association between perceived risk and continuance intention 19
Supplementary Table A15. Pooled regression coefficient of the association between perceived risk and continuance intention 19
Supplementary Figure A16. Forest plot of the random-effects meta-analysis of the association between perceived value and continuance intention 19
Supplementary Table A16. Pooled regression coefficient of the association between perceived value and continuance intention 19
Supplementary Figure A17. Forest plot of the random-effects meta-analysis of the association between health empowerment and continuance intention 20
Supplementary Table A17. Pooled regression coefficient of the association between health empowerment and continuance intention 20
Supplementary Figure A18. Forest plot of the random-effects meta-analysis of the association between engagement and continuance intention 20
Supplementary Table A18. Pooled regression coefficient of the association between engagement and continuance intention 20
Supplementary Figure A19. Forest plot of the random-effects meta-analysis of the association between function gratification and continuance intention 21
Supplementary Table A19. Pooled regression coefficient of the association between function gratification and continuance intention 21

Supplementary Figure A20. Forest plot of the random-effects meta-analysis of the association between perceived usefulness and satisfaction 22
Supplementary Table A20. Pooled regression coefficient of the association between perceived usefulness and satisfaction 22
Supplementary Figure A21. Forest plot of the random-effects meta-analysis of the association between confirmation and satisfaction 23
Supplementary Table A21. Pooled regression coefficient of the association between confirmation and satisfaction 23
Supplementary Figure A22. Forest plot of the random-effects meta-analysis of the association between perceived ease of use and satisfaction 24
Supplementary Table A22. Pooled regression coefficient of the association between perceived ease of use and satisfaction 24
Supplementary Figure A23. Forest plot of the random-effects meta-analysis of the association between service quality and satisfaction 24
Supplementary Table A23. Pooled regression coefficient of the association between service quality and satisfaction 25
Supplementary Figure A24. Forest plot of the random-effects meta-analysis of the association between confirmation and perceived usefulness 25
Supplementary Table A24. Pooled regression coefficient of the association between confirmation and perceived usefulness 25
Supplementary Figure A25. Forest plot of the random-effects meta-analysis of the association between perceived ease of use and perceived usefulness 26
Supplementary Table A25. Pooled regression coefficient of the association between perceived ease of use and perceived usefulness 26
Supplementary Figure A26. Forest plot of the random-effects meta-analysis of the association between service quality and perceived usefulness 26
Supplementary Table A26. Pooled regression coefficient of the association between service quality and perceived usefulness 27
Supplementary Figure A27. Forest plot of the random-effects meta-analysis of the association between information quality and perceived usefulness 27
Supplementary Table A27. Pooled regression coefficient of the association between information quality and perceived usefulness 27
Supplementary Figure A28. Forest plot of the random-effects meta-analysis of the association between confirmation and perceived ease of use 27
Supplementary Table A28. Pooled regression coefficient of the association between confirmation and perceived ease of use 28
Supplementary Table 2. Subgroup analysis by research quality 29
Supplementary Figure B1. Funnel Plot for all studies reporting on the regression coefficient of satisfaction on continuance intention 32
Supplementary Figure B2. Funnel Plot for all studies reporting on the regression coefficient of perceived usefulness on continuance intention 33
Supplementary Figure B3. Funnel Plot for all studies reporting on the regression coefficient of perceived ease of use on continuance intention 34
Supplementary Figure B4. Funnel Plot for all studies reporting on the regression coefficient of

trust on continuance intention 35
Supplementary Figure B5. Funnel Plot for all studies reporting on the regression coefficient of social influence on continuance intention 36
Supplementary Figure B6. Funnel Plot for all studies reporting on the regression coefficient of performance expectancy on continuance intention 37
Supplementary Figure B7. Funnel Plot for all studies reporting on the regression coefficient of facilitating conditions on continuance intention 38
Supplementary Figure B8. Funnel Plot for all studies reporting on the regression coefficient of service quality on continuance intention 39
Supplementary Figure B9. Funnel Plot for all studies reporting on the regression coefficient of attitude on continuance intention 40
Supplementary Figure B10. Funnel Plot for all studies reporting on the regression coefficient of habit on continuance intention 41
Supplementary Figure B11. Funnel Plot for all studies reporting on the regression coefficient of innovation on continuance intention 42
Supplementary Figure B12. Funnel Plot for all studies reporting on the regression coefficient of self-efficacy on continuance intention 43
Supplementary Figure B13. Funnel Plot for all studies reporting on the regression coefficient of perceived enjoyment on continuance intention 44
Supplementary Figure B14. Funnel Plot for all studies reporting on the regression coefficient of perceived quality of health life on continuance intention 45
Supplementary Figure B15. Funnel Plot for all studies reporting on the regression coefficient of perceived risk on continuance intention 46
Supplementary Figure B16. Funnel Plot for all studies reporting on the regression coefficient of perceived value on continuance intention 47
Supplementary Figure B17. Funnel Plot for all studies reporting on the regression coefficient of health empowerment on continuance intention 48
Supplementary Figure B18. Funnel Plot for all studies reporting on the regression coefficient of engagement on continuance intention 49
Supplementary Figure B19. Funnel Plot for all studies reporting on the regression coefficient of function gratification on continuance intention 50
Supplementary Figure B20. Funnel Plot for all studies reporting on the regression coefficient of perceived usefulness on satisfaction 51
Supplementary Figure B21. Funnel Plot for all studies reporting on the regression coefficient of confirmation on satisfaction 52
Supplementary Figure B22. Funnel Plot for all studies reporting on the regression coefficient of perceived ease of use on satisfaction 53
Supplementary Figure B23. Funnel Plot for all studies reporting on the regression coefficient of service quality on satisfaction 54
Supplementary Figure B24. Funnel Plot for all studies reporting on the regression coefficient of confirmation on perceived usefulness 55
Supplementary Figure B25. Funnel Plot for all studies reporting on the regression coefficient of perceived ease of use on perceived usefulness 56
Supplementary Figure B26. Funnel Plot for all studies reporting on the regression coefficient

of service quality on perceived usefulness 57
Supplementary Figure B27. Funnel Plot for all studies reporting on the regression coefficient of information quality on perceived usefulness 58
Supplementary Figure B28. Funnel Plot for all studies reporting on the regression coefficient of confirmation on perceived ease of use 59
Supplementary Table 3. PRISMA checklist 60
Supplementary Table 4. Query strategy and results 64

JBI CRITICAL APPRAISAL CHECKLIST FOR ANALYTICAL CROSS-SECTIONAL STUDIES

item 1: Were the criteria for inclusion in the sample clearly defined?

item 2: Were the study subjects and the setting described in detail?

item 3: Was the exposure measured in a valid and reliable way?

item 4: Were objective, standard criteria used for measurement of the condition?

item 5: Were confounding factors identified?

item 6: Were strategies to deal with confounding factors stated?

item 7: Were the outcomes measured in a valid and reliable way?

item 8: Was appropriate statistical analysis used?

Supplementary Table 1. Quality assessment of included prospective studies

studies	item 1	item 2	item 3	item 4	item 5	item 6	item 7	item 8	Overall appraisal	Researcher
Park et al,2018	2	1	1	1	0	0	1	1	7	Tong Wang
Hamari and Koivisto,2015	0	2	2	2	2	0	2	2	12	Tong Wang
Lee and Choo, 2017	1	1	2	2	1	2	2	2	13	Tong Wang
Rho et al,2015	1	2	1	2	2	2	1	2	13	Tong Wang
Li et al,2019	1	1	2	2	1	2	2	2	13	Tong Wang
Gu et al,2018	2	2	2	0	1	0	2	2	10	Tong Wang
Choo et al,2020	2	2	2	2	0	0	2	2	12	Tong Wang
Ahmad et al,2020	2	0	1	0	2	0	1	2	8	Tong Wang
Hsiao and Chen,2019	1	2	2	1	2	0	2	2	12	Tong Wang
Kaium et al,2020	2	2	1	2	2	0	1	2	12	Tong Wang

Akter et al,2011	2	2	2	2	2	0	2	2	14	Tong Wang
Meng et al,2021	1	1	2	2	2	2	2	2	14	Tong Wang
Zhang et al,2018	2	2	2	2	2	2	2	2	16	Tong Wang
Esmaeilzadeh, 2020	2	2	2	2	2	2	2	2	16	Tong Wang
Yin et al,2021	2	2	2	1	2	0	2	2	13	Tong Wang
Esmaeilzadeh, 2021	2	2	2	2	2	0	2	2	14	Tong Wang
Cho,2016	0	1	1	2	1	2	1	1	9	Tong Wang
Wang T et al, 2021	2	2	2	2	2	2	2	2	16	Tong Wang
Uei et al, 2013	2	2	1	2	1	2	1	1	12	Tong Wang
Lee and Kim,2021	2	2	2	1	1	0	2	2	13	Tong Wang
Akter et al,2010	2	2	2	2	2	0	2	2	14	Tong Wang
Sharma and Khadka,2019	1	2	2	2	1	0	2	2	12	Tong Wang
Devina et al,2019	2	2	1	1	2	0	1	1	10	Tong Wang
Damberg ,2021	2	2	2	2	2	0	2	2	14	Tong Wang
Liu et al,2019	2	2	2	2	2	2	2	2	16	Tong Wang
Akter et al,2013	2	2	2	1	1	0	2	2	12	Tong Wang
Soni et al,2021	1	1	2	1	1	0	2	2	10	Tong Wang
Song et al,2021	2	2	2	2	2	0	2	2	14	Tong Wang
Huang and Ren, 2020	2	2	2	1	2	2	2	2	15	Tong Wang
Yuan et al,2015	0	1	2	2	1	0	2	1	9	Tong Wang
Grenier Ouimet et al,2020	2	2	2	2	2	2	2	2	16	Tong Wang
Tsai et al,2014	2	1	1	2	1	2	1	2	12	Tong Wang
Liu et al,2021	2	2	2	2	2	2	2	2	16	Tong Wang
Kim et al,2019	2	2	2	2	1	0	2	2	13	Tong Wang
Akter et al,2021	2	1	2	2	2	0	2	2	13	Tong Wang

Lee SM and Lee DH,2020	1	1	2	2	2	0	2	2	12	Tong Wang
Guo et al,2020	0	1	2	1	2	2	2	2	12	Tong Wang
Beldad and Hegner,2018	0	1	1	2	1	0	1	2	8	Tong Wang
Meng et al,2020	0	1	2	1	2	0	2	2	10	Tong Wang
Luo et al,2021	2	2	2	2	2	2	2	2	16	Tong Wang
Hong et al,2019	1	1	2	2	2	2	2	2	14	Tong Wang
Leung and Chen,2019	2	2	2	2	2	2	2	2	16	Tong Wang
Paré et al,2018	0	1	2	2	2	0	2	2	11	Tong Wang
Akter et al,2013	1	2	2	2	2	0	2	2	13	Tong Wang
Birkmeyer et al,2021	2	2	2	2	2	0	2	2	14	Tong Wang
Kim and Han,2021	0	1	2	2	2	2	2	2	13	Tong Wang
Hartono et al,2021	0	0	2	2	2	0	2	2	10	Tong Wang
Kim et al,2019	0	1	1	2	2	2	1	2	11	Tong Wang
Zhang and Xu,2020	2	2	2	1	1	0	2	2	12	Tong Wang
Chiu et al,2020	2	1	2	2	2	0	2	2	13	Tong Wang
Jaana and Paré,2020	1	2	1	2	2	0	1	2	11	Tong Wang
Chen et al,2018	2	2	2	2	2	2	2	2	16	Tong Wang
Chen SC et al,2014	2	0	2	2	2	0	2	2	12	Tong Wang
Hossain,2016	1	1	2	1	2	0	2	2	11	Tong Wang
Chen et al,2020	1	2	2	2	2	0	2	2	13	Tong Wang
Anil Kumar and Natarajan,2020	1	2	1	1	2	0	1	2	10	Tong Wang
Hsieh et al,2016	2	2	2	2	2	0	2	2	14	Tong Wang
Choi and Lee,2015	0	0	1	1	0	0	1	1	4	Tong Wang
Park et al,2018	2	1	1	1	1	0	1	2	9	Jun Liang
Hamari and Koivisto,2015	0	2	2	2	2	0	2	2	12	Jun Liang

Lee and Choo, 2017	1	1	2	2	1	2	2	2	13	Jun Liang
Rho et al,2015	1	2	1	2	2	2	1	2	13	Jun Liang
Li et al,2019	1	1	2	2	1	2	2	2	13	Jun Liang
Gu et al,2018	2	2	2	1	2	0	2	2	12	Jun Liang
Choo et al,2020	2	1	2	2	1	0	2	2	12	Jun Liang
Ahmad et al,2020	2	0	1	0	2	0	1	2	8	Jun Liang
Hsiao and Chen,2019	1	2	2	2	2	0	2	2	13	Jun Liang
Kaium et al,2020	2	1	1	2	2	0	1	2	11	Jun Liang
Akter et al,2011	1	0	2	2	2	0	2	2	11	Jun Liang
Meng et al,2021	1	1	2	2	2	2	2	2	14	Jun Liang
Zhang et al,2018	2	2	2	2	2	2	2	2	16	Jun Liang
Esmaeilzadeh, 2020	2	2	2	2	2	2	2	2	16	Jun Liang
Yin et al,2021	2	1	2	1	2	0	2	2	12	Jun Liang
Esmaeilzadeh, 2021	2	2	2	2	2	0	2	2	14	Jun Liang
Cho,2016	0	1	1	2	1	2	1	1	9	Jun Liang
Wang T et al, 2021	2	2	2	2	2	2	2	2	16	Jun Liang
Uei et al, 2013	2	2	1	2	1	2	1	1	12	Jun Liang
Lee and Kim,2021	2	2	2	1	1	0	2	2	13	Jun Liang
Akter et al,2010	2	2	2	2	2	0	2	2	14	Jun Liang
Sharma and Khadka,2019	1	2	2	1	1	0	2	2	11	Jun Liang
Devina et al,2019	2	2	1	1	2	0	1	1	10	Jun Liang
Damberg ,2021	2	1	2	2	2	0	2	2	13	Jun Liang
Liu et al,2019	2	2	2	2	2	2	2	2	16	Jun Liang
Akter et al,2013	2	2	2	1	1	0	2	2	12	Jun Liang
Soni et al,2021	1	1	2	1	1	0	2	2	10	Jun Liang

Song et al,2021	2	2	2	2	2	0	2	2	14	Jun Liang
Huang and Ren, 2020	2	1	2	1	2	2	2	2	14	Jun Liang
Yuan et al,2015	1	1	2	2	1	0	2	1	10	Jun Liang
Grenier Ouimet et al,2020	1	2	2	1	2	2	2	2	14	Jun Liang
Tsai et al,2014	2	2	1	2	1	2	1	2	13	Jun Liang
Liu et al,2021	2	1	2	2	2	2	2	2	15	Jun Liang
Kim et al,2019	2	2	2	2	1	0	2	2	13	Jun Liang
Akter et al,2021	2	1	2	2	2	0	2	2	13	Jun Liang
Lee SM and Lee DH,2020	1	1	2	2	2	0	2	2	12	Jun Liang
Guo et al,2020	0	1	2	1	2	2	2	2	12	Jun Liang
Beldad and Hegner,2018	0	1	1	2	1	0	1	2	8	Jun Liang
Meng et al,2020	0	1	2	1	2	0	2	2	10	Jun Liang
Luo et al,2021	2	2	2	2	2	2	2	2	16	Jun Liang
Hong et al,2019	1	2	2	2	2	2	2	2	15	Jun Liang
Leung and Chen,2019	2	2	2	2	2	2	2	2	16	Jun Liang
Paré et al,2018	1	1	2	2	2	0	2	2	12	Jun Liang
Akter et al,2013	1	2	2	2	2	0	2	2	13	Jun Liang
Birkmeyer et al,2021	2	2	2	2	2	0	2	2	14	Jun Liang
Kim and Han,2021	0	1	2	2	2	2	2	2	13	Jun Liang
Hartono et al,2021	0	0	2	2	2	0	2	2	10	Jun Liang
Kim et al,2019	0	1	1	2	2	2	1	2	11	Jun Liang
Zhang and Xu,2020	2	2	2	1	1	0	2	2	12	Jun Liang
Chiu et al,2020	2	1	2	2	2	0	2	2	13	Jun Liang
Jaana and Paré,2020	1	2	1	2	2	0	1	2	11	Jun Liang
Chen et al,2018	2	1	2	2	2	2	2	2	15	Jun Liang

Chen SC et al,2014	2	0	2	2	2	0	2	2	12	Jun Liang
Hossain,2016	1	1	2	1	2	0	2	2	11	Jun Liang
Chen et al,2020	1	2	2	2	2	0	2	2	13	Jun Liang
Anil Kumar and Natarajan,2020	1	2	1	1	2	0	1	2	10	Jun Liang
Hsieh et al,2016	2	2	2	2	2	0	2	2	14	Jun Liang
Choi and Lee,2015	0	1	1	1	0	1	1	1	6	Jun Liang

Supplementary Figure A1. Forest plot of the random-effects meta-analysis of the association between satisfaction and continuance intention

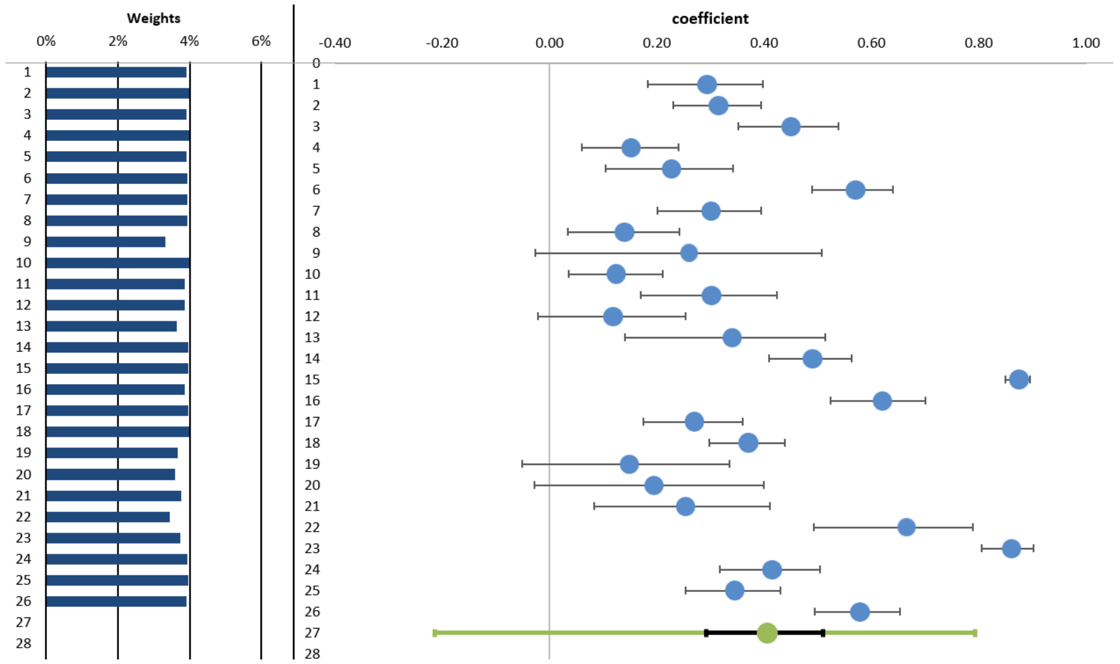

Supplementary Table A1. Pooled regression coefficient of the association between satisfaction and continuance intention

N	Study name	Coefficient	CI Lower limit	CI Upper limit	Weight
1	Akter et al,2010	0.294	0.183	0.397	3.92%
2	Akter et al,2013	0.315	0.231	0.394	3.98%
3	Akter et al,2013	0.450	0.351	0.539	3.92%
4	Anil Kumar and Natarajan,2020	0.152	0.060	0.241	3.98%
5	Birkmeyer et al,2021	0.227	0.105	0.342	3.91%
6	Chen SC et al,2014	0.570	0.490	0.641	3.94%
7	Chiu et al,2020	0.301	0.201	0.395	3.95%
8	Cho,2016	0.140	0.034	0.243	3.95%
9	Choi and Lee,2015	0.260	-0.027	0.507	3.33%
10	Gu et al,2018	0.124	0.036	0.210	3.99%
11	Hossain,2016	0.302	0.169	0.424	3.86%
12	Hsiao and Chen,2019	0.118	-0.022	0.253	3.87%
13	Hsieh et al,2016	0.340	0.140	0.513	3.63%
14	Jaana and Paré,2020	0.490	0.410	0.563	3.96%
15	Kaium et al,2020	0.875	0.849	0.896	3.97%
16	Kim et al,2019	0.620	0.523	0.701	3.86%
17	Leung and Chen,2019	0.270	0.175	0.360	3.96%
18	Paré et al,2018	0.370	0.297	0.438	4.00%
19	Rho et al,2015	0.148	-0.051	0.336	3.68%
20	Rho et al,2015	0.194	-0.029	0.398	3.59%
21	Song et al,2021	0.254	0.083	0.410	3.76%
22	Tsai et al,2014	0.666	0.492	0.789	3.44%
23	Uei et al, 2013	0.861	0.806	0.902	3.74%
24	Wang T et al, 2021	0.415	0.317	0.504	3.94%
25	Zhang and Xu,2020	0.345	0.253	0.431	3.96%
26	Zhang et al,2018	0.579	0.494	0.653	3.92%

Supplementary Figure A2. Forest plot of the random-effects meta-analysis of the association between perceived usefulness and continuance intention

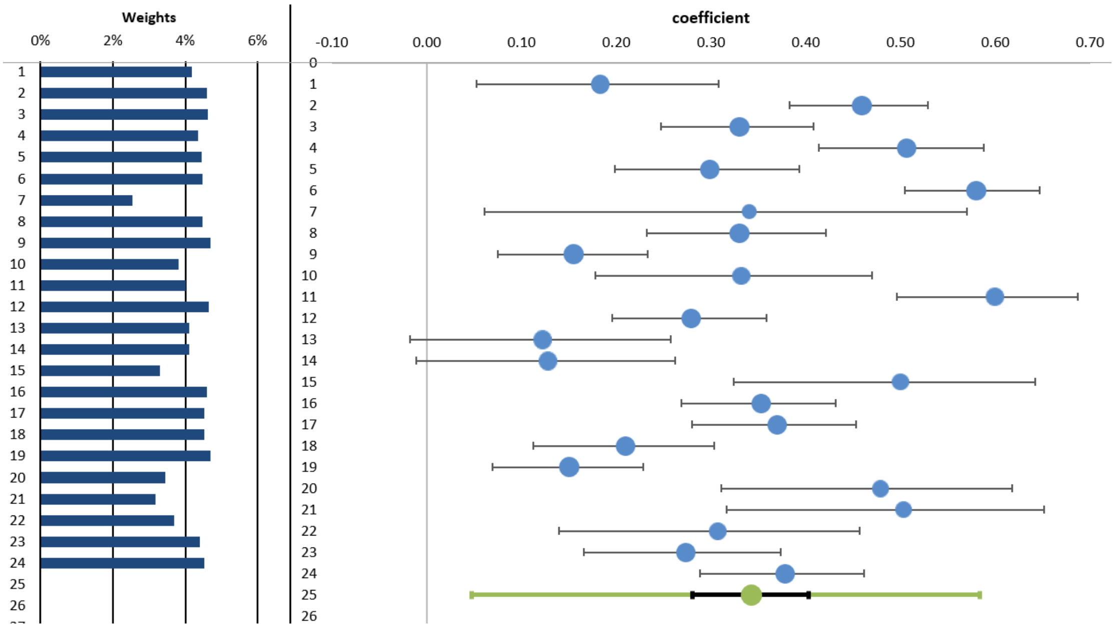

Supplementary Table A2. Pooled regression coefficient of the association between perceived usefulness and continuance intention

N	Study name	Regression	CI Lower limit	CI Upper limit	Weight
1	Ahmad et al,2020	0.183	0.052	0.308	4.19%
2	Anil Kumar and Natarajan,2020	0.459	0.383	0.529	4.60%
3	Beldad and Hegner,2018	0.330	0.247	0.408	4.62%
4	Chen et al,2018	0.506	0.414	0.588	4.36%
5	Chiu et al,2020	0.299	0.199	0.393	4.46%
6	Cho,2016	0.580	0.505	0.646	4.47%
7	Choi and Lee,2015	0.340	0.061	0.570	2.55%
8	Choo et al,2020	0.330	0.232	0.421	4.47%
9	Damberg ,2021	0.155	0.075	0.233	4.71%
10	Devina et al,2019	0.332	0.178	0.470	3.83%
11	Grenier Ouimet et al,2020	0.600	0.496	0.687	4.01%
12	Gu et al,2018	0.279	0.195	0.359	4.64%
13	Hamari and Koivisto,2015	0.122	-0.018	0.257	4.11%
14	Hsiao and Chen,2019	0.128	-0.011	0.263	4.11%
15	Hsieh et al,2016	0.500	0.324	0.642	3.30%
16	Huang and Ren, 2020	0.353	0.269	0.432	4.60%
17	Jaana and Paré,2020	0.370	0.280	0.454	4.52%
18	Leung and Chen,2019	0.210	0.112	0.304	4.53%
19	Paré et al,2018	0.150	0.069	0.229	4.70%
20	Rho et al,2015	0.479	0.311	0.618	3.44%
21	Rho et al,2015	0.503	0.317	0.652	3.17%
22	Song et al,2021	0.307	0.140	0.457	3.70%
23	Wang T et al, 2021	0.273	0.166	0.374	4.40%
24	Zhang and Xu,2020	0.378	0.288	0.461	4.52%

Supplementary Figure A3. Forest plot of the random-effects meta-analysis of the association between perceived ease of use and continuance intention

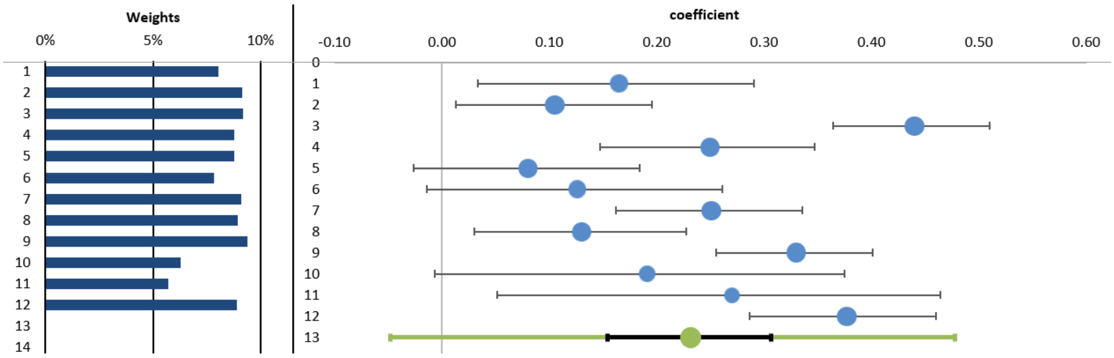

Supplementary Table A3. Pooled regression coefficient of the association between perceived ease of use and continuance intention

N	Study name	Regression	CI Lower limit	CI Upper limit	Weight
1	Ahmad et al,2020	0.165	0.034	0.291	8.06%
2	Anil Kumar and Natarajan,2020	0.105	0.013	0.195	9.12%
3	Beldad and Hegner,2018	0.440	0.364	0.510	9.18%
4	Cho,2016	0.250	0.148	0.347	8.76%
5	Choo et al,2020	0.080	-0.026	0.184	8.77%
6	Hamari and Koivisto,2015	0.126	-0.014	0.261	7.85%
7	Huang and Ren, 2020	0.251	0.162	0.336	9.11%
8	Jaana and Paré,2020	0.130	0.030	0.227	8.92%
9	Paré et al,2018	0.330	0.255	0.401	9.38%
10	Rho et al,2015	0.191	-0.007	0.375	6.27%
11	Rho et al,2015	0.270	0.051	0.464	5.69%
12	Zhang and Xu,2020	0.377	0.287	0.461	8.90%

Supplementary Figure A4. Forest plot of the random-effects meta-analysis of the association between trust and continuance intention

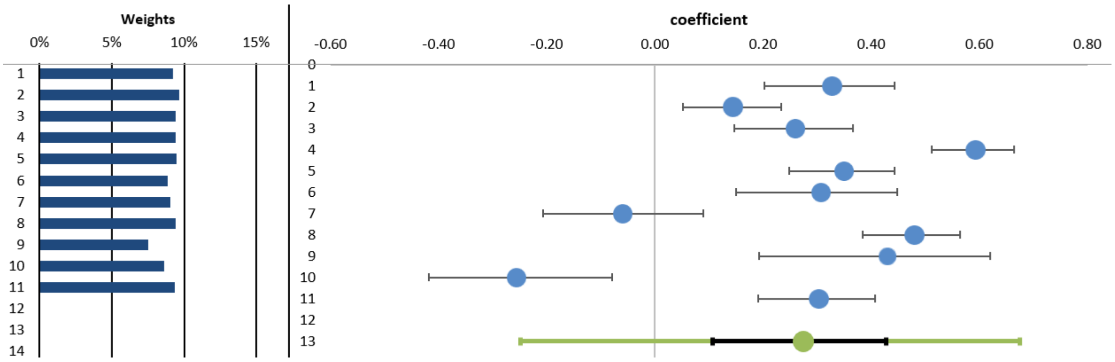

Supplementary Table A4. Pooled regression coefficient of the association between trust and continuance intention

N	Study name	Regression	CI Lower limit	CI Upper limit	Weight
1	Akter et al,2011	0.328	0.203	0.443	9.22%
2	Anil Kumar and Natarajan,2020	0.144	0.052	0.233	9.64%
3	Chen et al,2018	0.260	0.148	0.366	9.41%
4	Chen et al,2020	0.593	0.512	0.664	9.41%
5	Chen SC et al,2014	0.350	0.248	0.444	9.47%
6	Devina et al,2019	0.307	0.150	0.448	8.86%
7	Grenier Ouimet et al,2020	-0.060	-0.206	0.089	9.06%
8	Hong et al,2019	0.480	0.385	0.565	9.41%
9	Tsai et al,2014	0.430	0.192	0.620	7.51%
10	Uei et al, 2013	-0.256	-0.418	-0.079	8.63%
11	Zhang et al,2018	0.303	0.191	0.408	9.38%

Supplementary Figure A5. Forest plot of the random-effects meta-analysis of the association between social influence and continuance intention

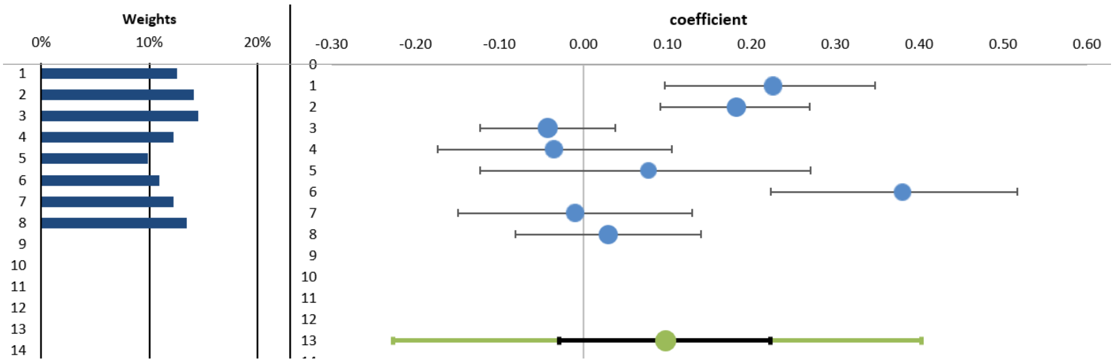

Supplementary Table A5. Pooled regression coefficient of the association between social influence and continuance intention

N	Study name	Regression	CI Lower limit	CI Upper limit	Weight
1	Ahmad et al,2020	0.226	0.097	0.348	12.56%
2	Anil Kumar and Natarajan,2020	0.182	0.091	0.270	14.12%
3	Damberg ,2021	-0.043	-0.123	0.038	14.53%
4	Hamari and Koivisto,2015	-0.035	-0.174	0.105	12.25%
5	Hartono et al,2021	0.077	-0.123	0.271	9.89%
6	Kim et al,2019	0.380	0.223	0.517	10.95%
7	Park et al,2018	-0.010	-0.149	0.129	12.26%
8	Yuan et al,2015	0.030	-0.081	0.140	13.43%

Supplementary Figure A6. Forest plot of the random-effects meta-analysis of the association between performance expectancy and continuance intention

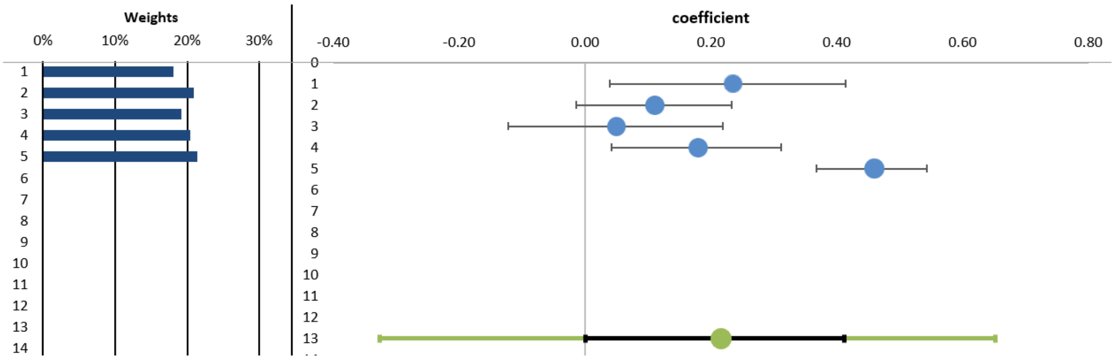

Supplementary Table A6. Pooled regression coefficient of the association between performance expectancy and continuance intention

N	Study name	Regression	CI Lower limit	CI Upper limit	Weight
1	Hartono et al,2021	0.235	0.039	0.414	18.05%
2	Kim and Han,2021	0.111	-0.014	0.232	20.95%
3	Kim et al,2019	0.050	-0.122	0.219	19.16%
4	Park et al,2018	0.180	0.042	0.311	20.41%
5	Yuan et al,2015	0.460	0.368	0.543	21.43%

Supplementary Figure A7. Forest plot of the random-effects meta-analysis of the association between facilitating conditions and continuance intention

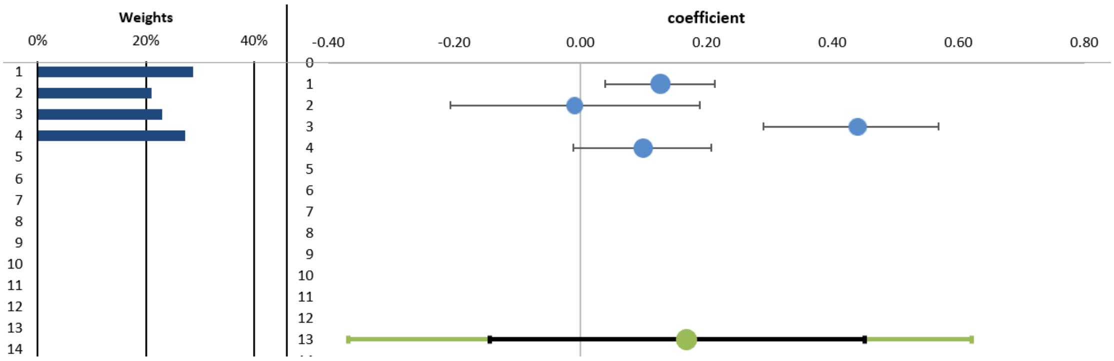

Supplementary Table A7. Pooled regression coefficient of the association between facilitating conditions and continuance intention

N	Study name	Regression	CI Lower limit	CI Upper limit	Weight
1	Gu et al,2018	0.127	0.039	0.213	28.65%
2	Hartono et al,2021	-0.009	-0.206	0.189	21.09%
3	Kim et al,2019	0.440	0.291	0.568	23.00%
4	Yuan et al,2015	0.100	-0.011	0.208	27.27%

Supplementary Figure A8. Forest plot of the random-effects meta-analysis of the association between service quality and continuance intention

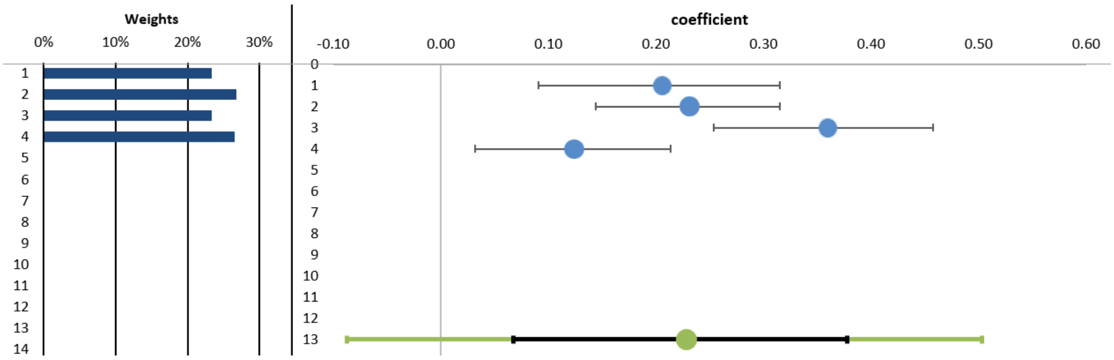

Supplementary Table A8. Pooled regression coefficient of the association between service quality and continuance intention

N	Study name	Regression	CI Lower limit	CI Upper limit	Weight
1	Akter et al,2010	0.206	0.091	0.315	23.31%
2	Akter et al,2013	0.231	0.144	0.315	26.82%
3	Akter et al,2013	0.360	0.254	0.458	23.31%
4	Anil Kumar and Natarajan,2020	0.124	0.032	0.214	26.56%

Supplementary Figure A9. Forest plot of the random-effects meta-analysis of the association between attitude and continuance intention

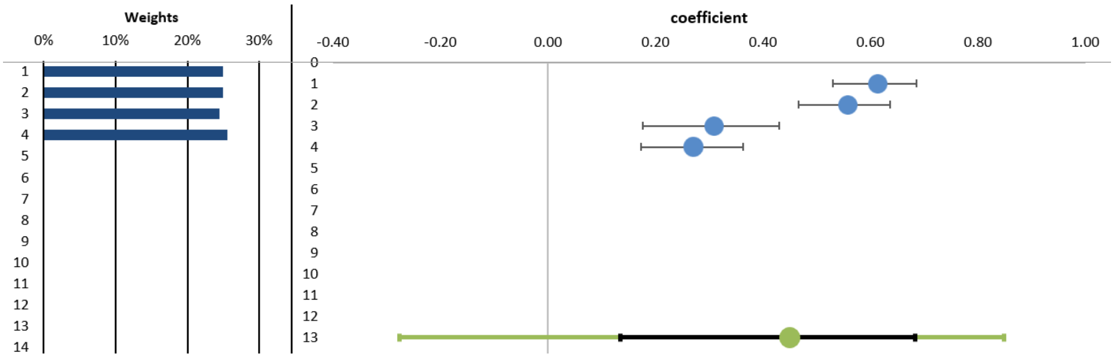

Supplementary Table A9. Pooled regression coefficient of the association between attitude and continuance intention

N	Study name	Regression	CI Lower limit	CI Upper limit	Weight
1	Birkmeyer et al,2021	0.614	0.530	0.686	24.94%
2	Guo et al,2020	0.558	0.467	0.638	24.99%
3	Hamari and Koivisto,2015	0.309	0.177	0.430	24.45%
4	Luo et al,2021	0.271	0.173	0.363	25.62%

Supplementary Figure A10. Forest plot of the random-effects meta-analysis of the association between habit and continuance intention

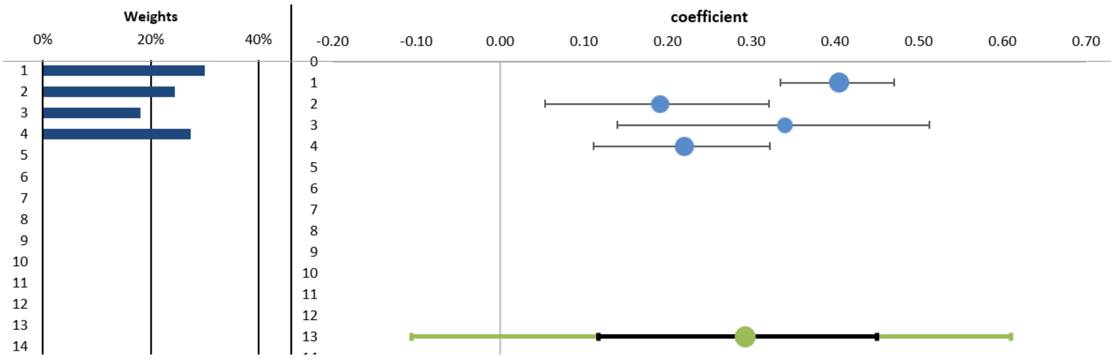

Supplementary Table A10. Pooled regression coefficient of the association between habit and continuance intention

N	Study name	Regression	CI Lower limit	CI Upper limit	Weight
1	Damberg ,2021	0.405	0.335	0.470	30.06%
2	Hsiao and Chen,2019	0.191	0.053	0.322	24.52%
3	Hsieh et al,2016	0.340	0.140	0.513	18.09%
4	Yuan et al,2015	0.220	0.112	0.323	27.33%

Supplementary Figure A11. Forest plot of the random-effects meta-analysis of the association between innovation and continuance intention

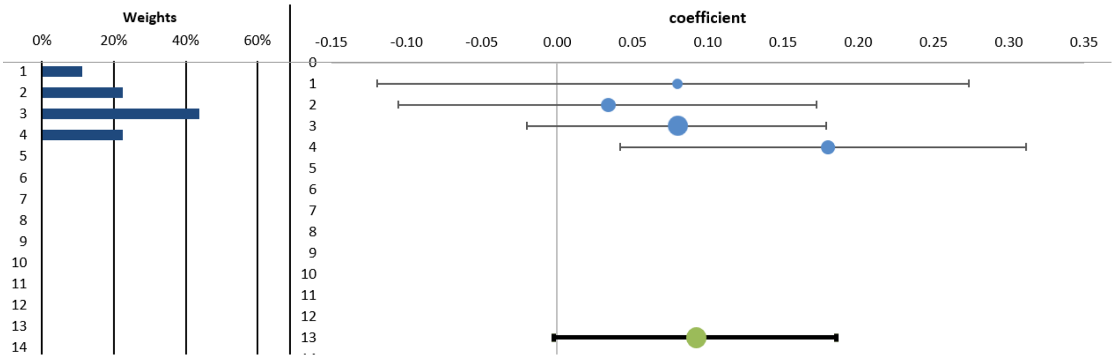

Supplementary Table A11. Pooled regression coefficient of the association between innovation and continuance intention

N	Study name	Regression	CI Lower limit	CI Upper limit	Weight
1	Hartono et al,2021	0.080	-0.120	0.273	11.16%
2	Hsiao and Chen,2019	0.034	-0.106	0.172	22.55%
3	Leung and Chen,2019	0.080	-0.020	0.179	43.74%
4	Park et al,2018	0.180	0.042	0.311	22.55%

Supplementary Figure A12. Forest plot of the random-effects meta-analysis of the association between self-efficacy and continuance intention

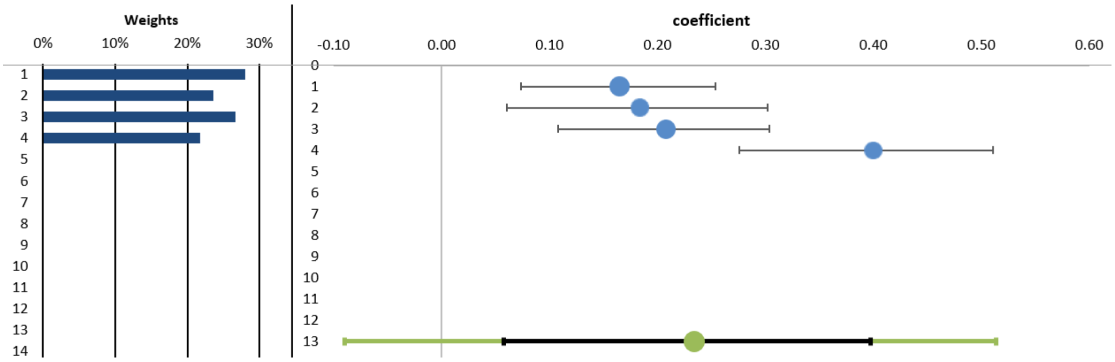

Supplementary Table A12. Pooled regression coefficient of the association between self-efficacy and continuance intention

N	Study name	Regression	CI Lower limit	CI Upper limit	Weight
1	Huang and Ren, 2020	0.165	0.073	0.254	28.02%
2	Kim and Han,2021	0.184	0.061	0.302	23.61%
3	Luo et al,2021	0.208	0.108	0.304	26.65%
4	Park et al,2018	0.400	0.276	0.511	21.72%

Supplementary Figure A13. Forest plot of the random-effects meta-analysis of the association between perceived enjoyment and continuance intention

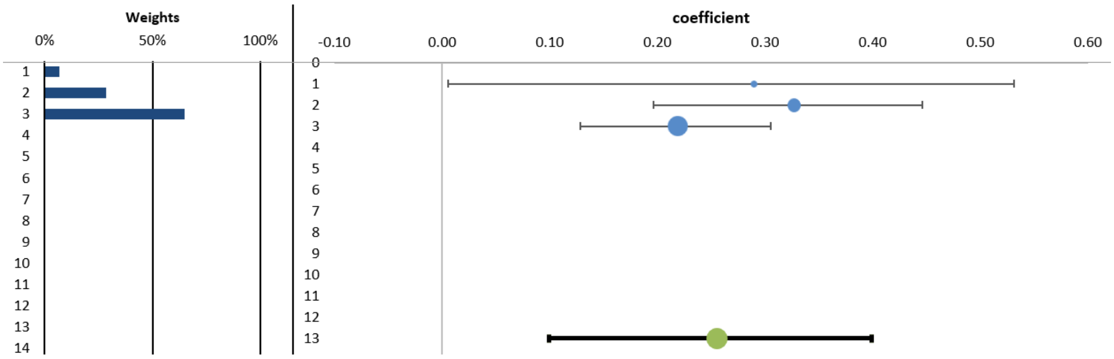

Supplementary Table A13. Pooled regression coefficient of the association between perceived enjoyment and continuance intention

N	Study name	Regression	CI Lower limit	CI Upper limit	Weight
1	Choi and Lee,2015	0.290	0.005	0.531	6.81%
2	Hamari and Koivisto,2015	0.327	0.196	0.446	28.55%
3	Huang and Ren, 2020	0.219	0.129	0.306	64.64%

Supplementary Figure A14. Forest plot of the random-effects meta-analysis of the association between perceived quality of health life and continuance intention

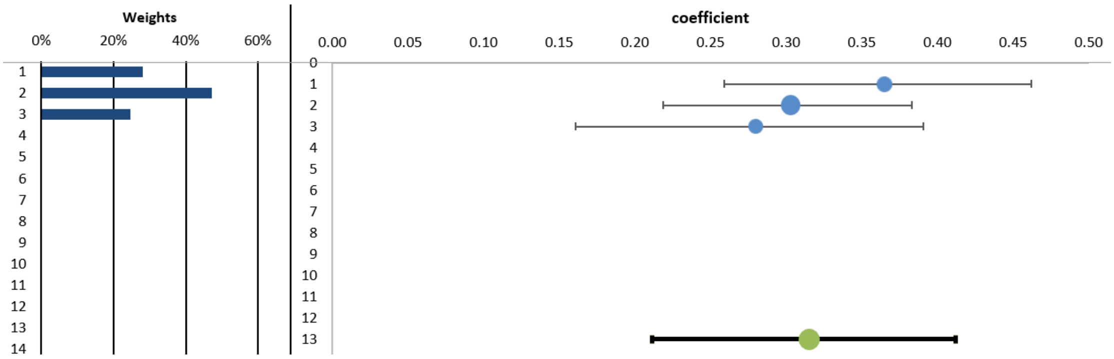

Supplementary Table A14. Pooled regression coefficient of the association between perceived quality of health life and continuance intention

N	Study name	Regression	CI Lower limit	CI Upper limit	Weight
1	Akter et al,2010	0.365	0.259	0.462	28.08%
2	Akter et al,2013	0.303	0.219	0.383	47.14%
3	Kim and Han,2021	0.280	0.161	0.391	24.77%

Supplementary Figure A15. Forest plot of the random-effects meta-analysis of the association between perceived risk and continuance intention

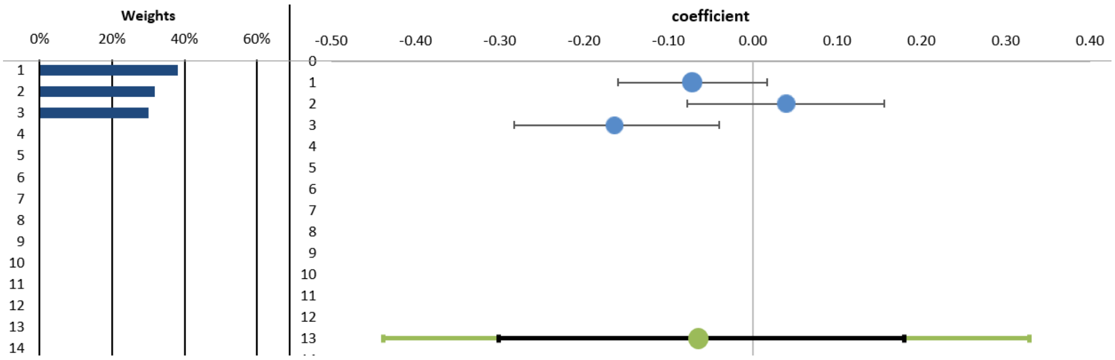

Supplementary Table A15. Pooled regression coefficient of the association between perceived risk and continuance intention

N	Study name	Regression	CI Lower limit	CI Upper limit	Weight
1	Gu et al,2018	-0.072	-0.159	0.017	38.11%
2	Hong et al,2019	0.040	-0.077	0.156	31.73%
3	Kim and Han,2021	-0.164	-0.283	-0.040	30.16%

Supplementary Figure A16. Forest plot of the random-effects meta-analysis of the association between perceived value and continuance intention

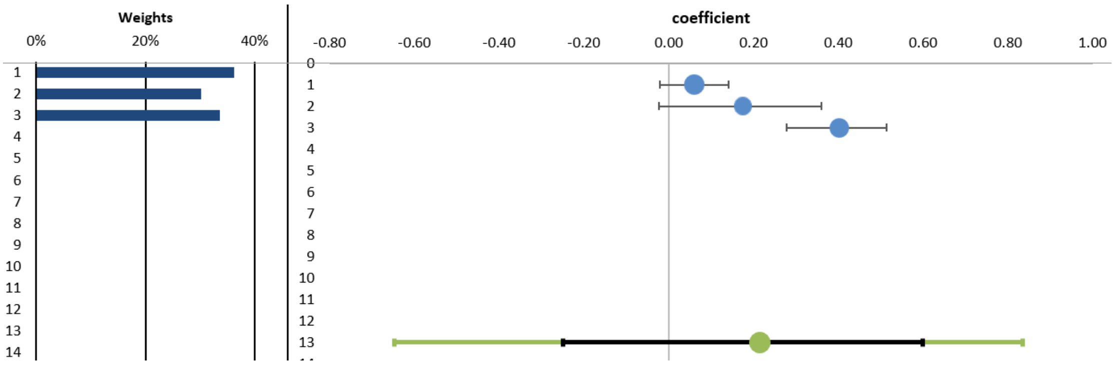

Supplementary Table A16. Pooled regression coefficient of the association between perceived value and continuance intention

#	Study name	Regression	CI Lower limit	CI Upper limit	Weight
1	Damberg ,2021	0.061	-0.020	0.141	36.24%
2	Hartono et al,2021	0.175	-0.024	0.360	30.20%
3	Hossain,2016	0.403	0.279	0.514	33.56%

Supplementary Figure A17. Forest plot of the random-effects meta-analysis of the association between health empowerment and continuance intention

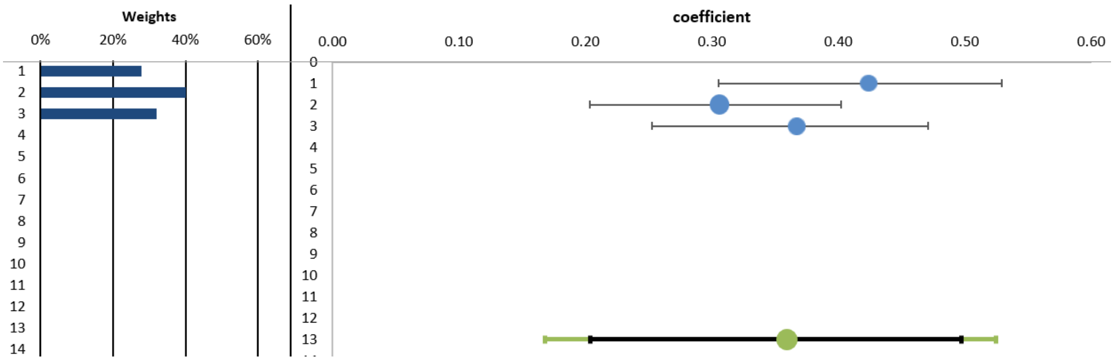

Supplementary Table A17. Pooled regression coefficient of the association between health empowerment and continuance intention

N	Study name	Regression	CI Lower limit	CI Upper limit	Weight
1	Akter et al,2021	0.424	0.305	0.530	27.88%
2	Liu et al,2021	0.306	0.203	0.402	40.15%
3	Sharma and Khadka,2019	0.367	0.253	0.471	31.98%

Supplementary Figure A18. Forest plot of the random-effects meta-analysis of the association between engagement and continuance intention

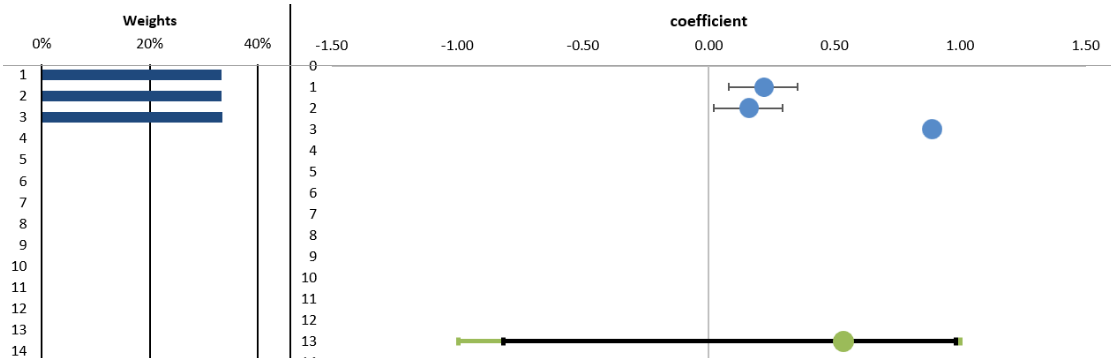

Supplementary Table A18. Pooled regression coefficient of the association between engagement and continuance intention

#	Study name	Regression	CI Lower limit	CI Upper limit	Weight
1	Kim et al,2019	0.220	0.080	0.352	33.30%
2	Park et al,2018	0.160	0.021	0.293	33.31%
3	Soni et al,2021	0.887	0.858	0.910	33.39%

Supplementary Figure A19. Forest plot of the random-effects meta-analysis of the association between function gratification and continuance intention

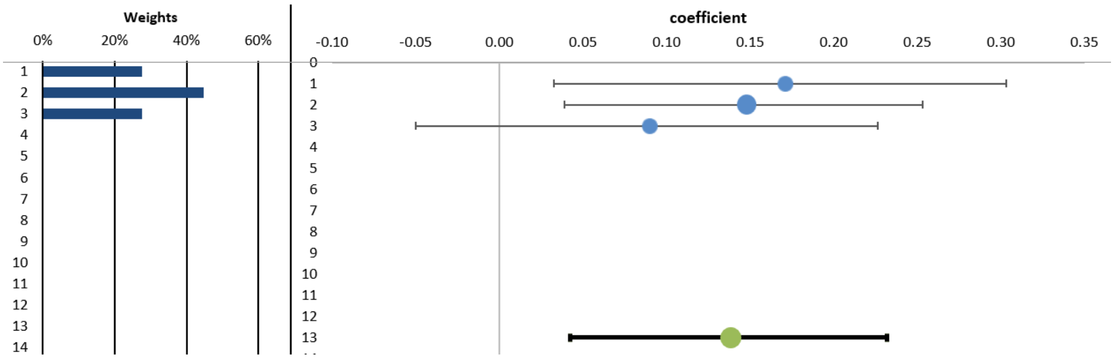

Supplementary Table A19. Pooled regression coefficient of the association between function gratification and continuance intention

N	Study name	Regression	CI Lower limit	CI Upper limit	Weight
1	Hsiao and Chen,2019	0.171	0.033	0.303	27.65%
2	Liu et al,2021	0.148	0.039	0.253	44.69%
3	Park et al,2018	0.090	-0.050	0.226	27.65%

Supplementary Figure A20. Forest plot of the random-effects meta-analysis of the association between perceived usefulness and satisfaction

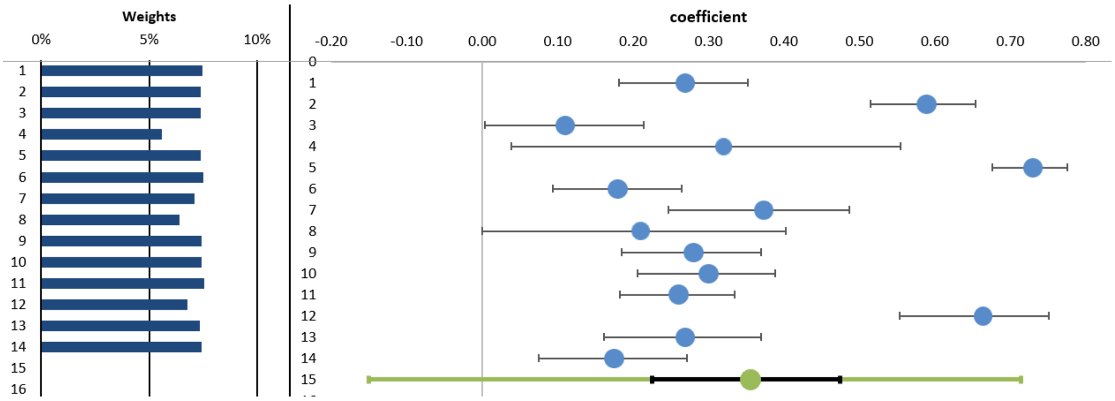

Supplementary Table A20. Pooled regression coefficient of the association between perceived usefulness and satisfaction

N	Study name	Regression	CI Lower limit	CI Upper limit	Weight
1	Anil Kumar and Natarajan,2020	0.269	0.181	0.353	7.46%
2	Chiu et al,2020	0.589	0.515	0.654	7.37%
3	Cho,2016	0.110	0.004	0.214	7.37%
4	Choi and Lee,2015	0.320	0.039	0.554	5.58%
5	Choo et al,2020	0.730	0.676	0.776	7.37%
6	Gu et al,2018	0.180	0.093	0.264	7.49%
7	Hsiao and Chen,2019	0.373	0.247	0.487	7.11%
8	Hsieh et al,2016	0.210	0.000	0.402	6.41%
9	Jaana and Paré,2020	0.280	0.185	0.370	7.41%
10	Leung and Chen,2019	0.300	0.206	0.388	7.41%
11	Paré et al,2018	0.260	0.182	0.334	7.53%
12	Song et al,2021	0.664	0.554	0.751	6.78%
13	Wang T et al, 2021	0.269	0.162	0.370	7.32%
14	Zhang and Xu,2020	0.175	0.075	0.271	7.40%

Supplementary Figure A21. Forest plot of the random-effects meta-analysis of the association between confirmation and satisfaction

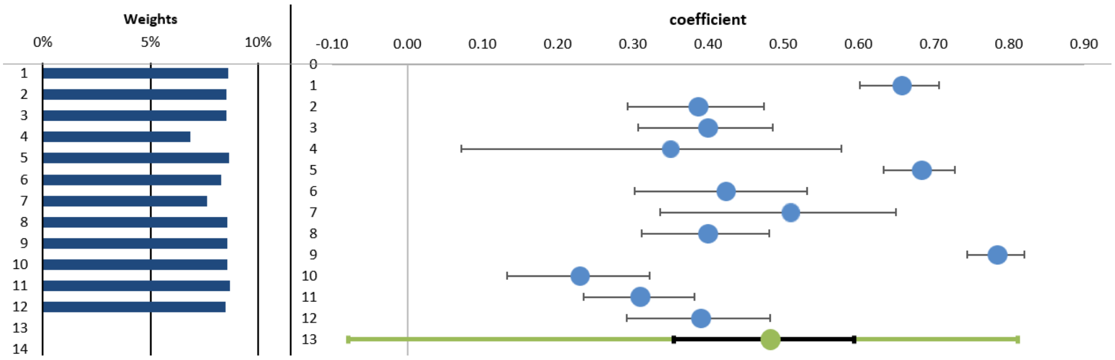

Supplementary Table A21. Pooled regression coefficient of the association between confirmation and satisfaction

N	Study name	Regression	CI Lower limit	CI Upper limit	Weight
1	Anil Kumar and Natarajan,2020	0.658	0.602	0.707	8.62%
2	Chiu et al,2020	0.387	0.293	0.474	8.53%
3	Cho,2016	0.400	0.307	0.486	8.53%
4	Choi and Lee,2015	0.350	0.072	0.577	6.83%
5	Gu et al,2018	0.684	0.634	0.728	8.64%
6	Hsiao and Chen,2019	0.424	0.303	0.532	8.30%
7	Hsieh et al,2016	0.510	0.336	0.650	7.65%
8	Jaana and Paré,2020	0.400	0.312	0.481	8.57%
9	Kaium et al,2020	0.785	0.744	0.820	8.58%
10	Leung and Chen,2019	0.230	0.133	0.323	8.57%
11	Paré et al,2018	0.310	0.234	0.382	8.68%
12	Wang T et al, 2021	0.391	0.291	0.482	8.49%

Supplementary Figure A22. Forest plot of the random-effects meta-analysis of the association between perceived ease of use and satisfaction

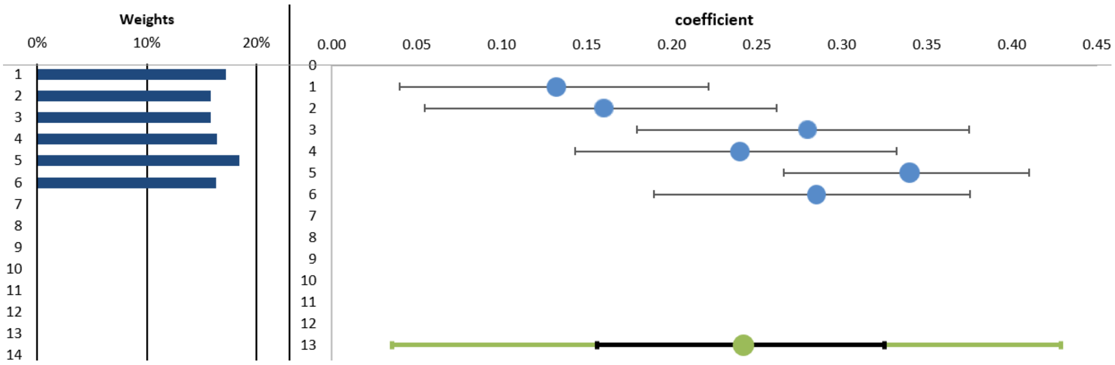

Supplementary Table A22. Pooled regression coefficient of the association between perceived ease of use and satisfaction

N	Study name	Regression	CI Lower limit	CI Upper limit	Weight
1	Anil Kumar and Natarajan,2020	0.132	0.040	0.222	17.25%
2	Cho,2016	0.160	0.055	0.262	15.79%
3	Choo et al,2020	0.280	0.180	0.375	15.83%
4	Jaana and Paré,2020	0.240	0.143	0.332	16.40%
5	Paré et al,2018	0.340	0.266	0.410	18.41%
6	Zhang and Xu,2020	0.285	0.189	0.375	16.33%

Supplementary Figure A23. Forest plot of the random-effects meta-analysis of the association between service quality and satisfaction

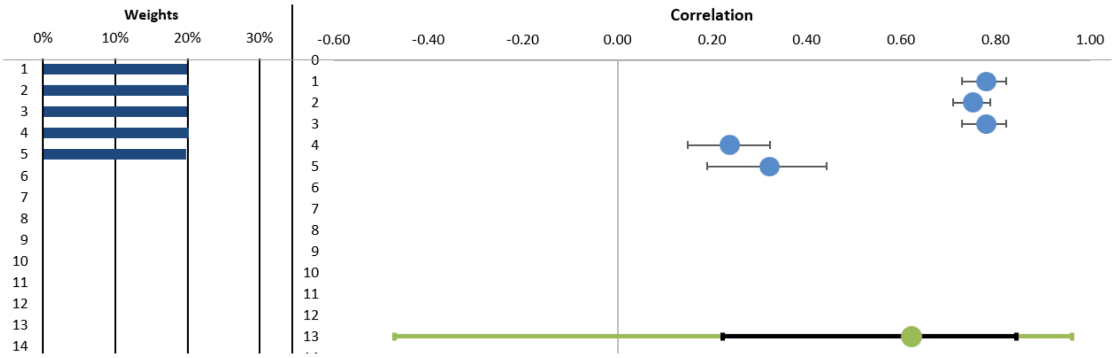

Supplementary Table A23. Pooled regression coefficient of the association between service quality and satisfaction

N	Study name	Regression	CI Lower limit	CI Upper limit	Weight
1	Akter et al,2010	0.780	0.730	0.822	19.97%
2	Akter et al,2013	0.753	0.711	0.790	20.14%
3	Akter et al,2013	0.780	0.730	0.822	19.97%
4	Anil Kumar and Natarajan,2020	0.238	0.149	0.323	20.13%
5	Hossain,2016	0.322	0.191	0.442	19.79%

Supplementary Figure A24. Forest plot of the random-effects meta-analysis of the association between confirmation and perceived usefulness

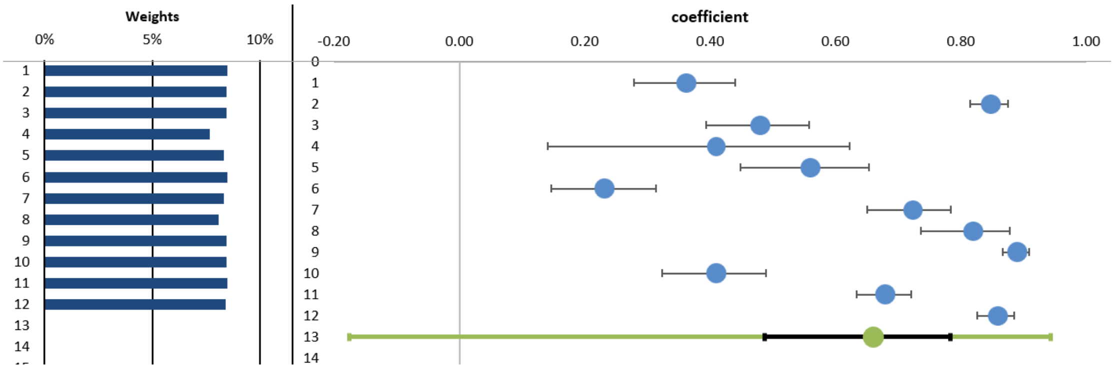

Supplementary Table A24. Pooled regression coefficient of the association between confirmation and perceived usefulness

N	Study name	Regression	CI Lower limit	CI Upper limit	Weight
1	Anil Kumar and Natarajan,2020	0.362	0.279	0.440	8.47%
2	Chiu et al,2020	0.848	0.815	0.875	8.43%
3	Cho,2016	0.480	0.394	0.558	8.43%
4	Choi and Lee,2015	0.410	0.142	0.622	7.67%
5	Grenier Ouimet et al,2020	0.560	0.449	0.654	8.31%
6	Gu et al,2018	0.232	0.147	0.314	8.48%
7	Hsiao and Chen,2019	0.724	0.650	0.784	8.34%
8	Hsieh et al,2016	0.820	0.737	0.879	8.06%
9	Jaana and Paré,2020	0.890	0.867	0.909	8.45%
10	Leung and Chen,2019	0.410	0.323	0.490	8.45%
11	Paré et al,2018	0.680	0.634	0.722	8.49%
12	Wang T et al, 2021	0.859	0.826	0.886	8.42%

Supplementary Figure A25. Forest plot of the random-effects meta-analysis of the association between perceived ease of use and perceived usefulness

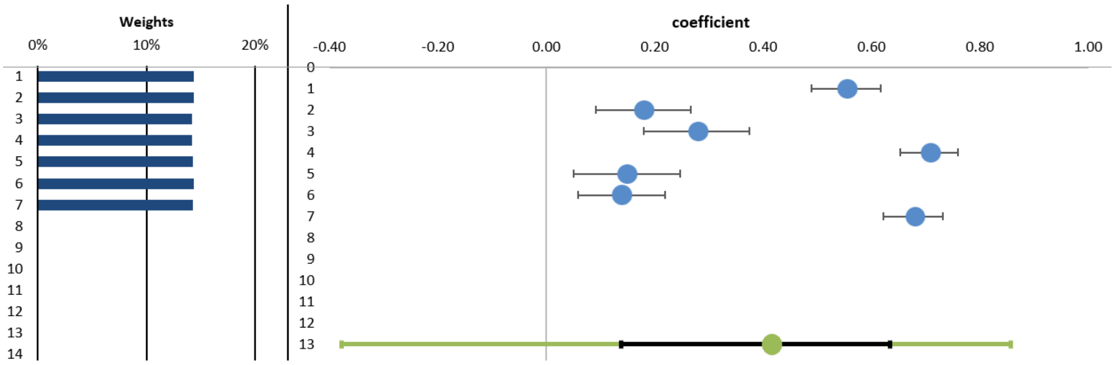

Supplementary Table A25. Pooled regression coefficient of the association between perceived ease of use and perceived usefulness

N	Study name	Regression	CI Lower limit	CI Upper limit	Weight
1	Anil Kumar and Natarajan,2020	0.556	0.489	0.617	14.32%
2	Beldad and Hegner,2018	0.180	0.091	0.266	14.33%
3	Cho,2016	0.280	0.179	0.375	14.22%
4	Choo et al,2020	0.710	0.653	0.759	14.22%
5	Jaana and Paré,2020	0.150	0.050	0.247	14.26%
6	Paré et al,2018	0.140	0.059	0.219	14.38%
7	Zhang and Xu,2020	0.681	0.623	0.732	14.26%

Supplementary Figure A26. Forest plot of the random-effects meta-analysis of the association between service quality and perceived usefulness

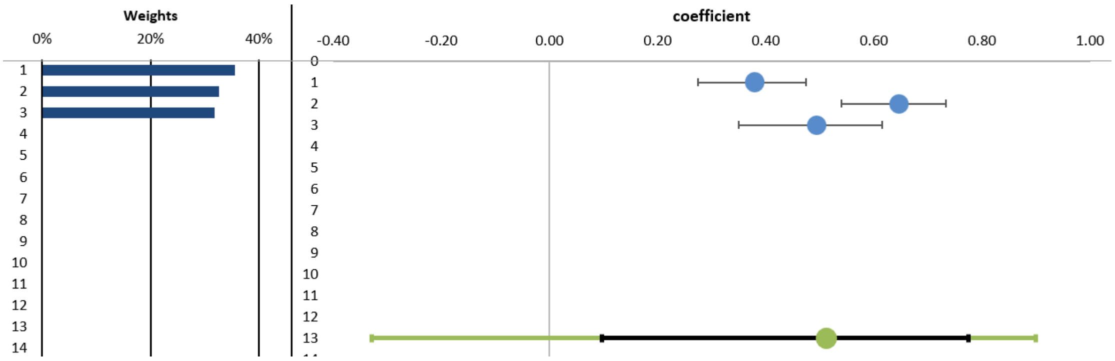

Supplementary Table A26. Pooled regression coefficient of the association between service quality and perceived usefulness

#	Study name	Regression	CI Lower limit	CI Upper limit	Weight
1	Chen et al,2018	0.380	0.275	0.475	35.65%
2	Devina et al,2019	0.647	0.541	0.733	32.55%
3	Song et al,2021	0.494	0.350	0.615	31.79%

Supplementary Figure A27. Forest plot of the random-effects meta-analysis of the association between information quality and perceived usefulness

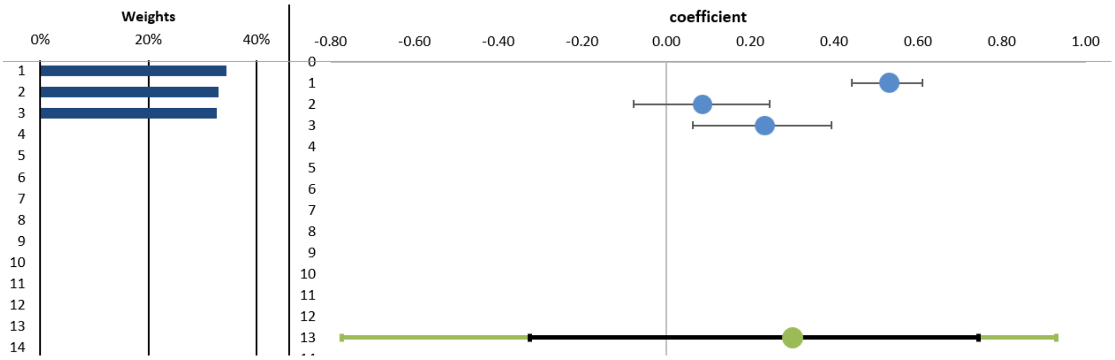

Supplementary Table A27. Pooled regression coefficient of the association between information quality and perceived usefulness

N	Study name	Regression	CI Lower limit	CI Upper limit	Weight
1	Chen et al,2018	0.531	0.442	0.610	34.43%
2	Devina et al,2019	0.087	-0.078	0.247	32.98%
3	Song et al,2021	0.235	0.063	0.393	32.60%

Supplementary Figure A28. Forest plot of the random-effects meta-analysis of the association between confirmation and perceived ease of use

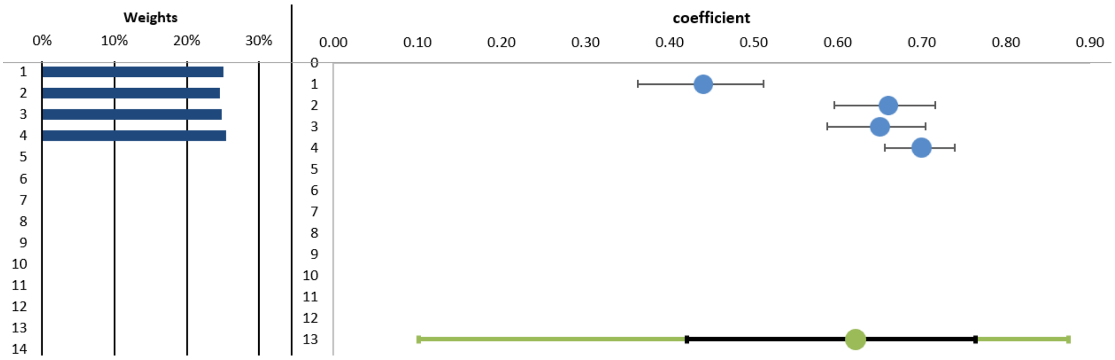

Supplementary Table A28. Pooled regression coefficient of the association between confirmation and perceived ease of use

#	Study name	Regression	CI Lower limit	CI Upper limit	Weight
1	Anil Kumar and Natarajan,2020	0.440	0.362	0.512	25.11%
2	Cho,2016	0.660	0.595	0.716	24.58%
3	Jaana and Paré,2020	0.650	0.588	0.704	24.81%
4	Paré et al,2018	0.700	0.656	0.739	25.49%

Supplementary Table 2. Subgroup analysis by research quality

N	Independent constructs	Dependent constructs	Sum of squares	*P* [a]	Subgroup	Total	Total sample	Combined Effect Size	*P* [b]	95% confidence interval	
										Low	High
1	satisfaction	continuance intention	0.265	.607	A	26	7165	0.406	<.001	0.292	0.509
					B	22	5825	0.445	<.001	0.321	0.554
2	perceived usefulness	continuance intention	0.056	.812	A	24	7214	0.343	<.001	0.280	0.403
					B	17	5029	0.332	<.001	0.252	0.408
3	perceived ease of use	continuance intention	0.020	.888	A	12	4015	0.232	<.001	0.154	0.307
					B	8	2520	0.224	<.001	0.129	0.314
4	trust	continuance intention	0.121	.728	A	10	2326	0.237	.001	0.073	0.388
					B	8	1727	0.240	.009	0.024	0.434
5	social influence	continuance intention	0.000	.999	A	8	2220	0.098	.068	0.098	-0.029
					B	3	925	0.098	.494	-0.479	0.616
6	performance expectancy	continuance intention	1.574	.210	A	5	1003	0.216	.005	0.001	0.413
					B	2	384	0.090	.002	-0.274	0.431
7	habit	continuance intention	0.074	.786	A	4	1199	0.293	<.001	0.118	0.450
					B	3	882	0.319	<.001	0.019	0.566

8	innovation	continuance intention	0.279	.597	A	4	890	0.092	.002	-0.002	0.185
					B	2	588	0.064	.003	-0.210	0.330
9	self-efficacy	continuance intention	0.751	.386	A	4	1268	0.234	<.001	0.057	0.397
					B	3	1067	0.184	<.001	0.127	0.241
10	perceived risk	continuance intention	0.001	.979	A	3	1027	-0.065	.259	-0.302	0.180
					B	2	533	-0.061	.549	-0.878	0.847
11	function gratification	continuance intention	0.106	.745	A	3	725	0.138	<.001	0.042	0.232
					B	2	524	0.157	<.001	0.012	0.295
12	perceived usefulness	satisfaction	0.322	.570	A	14	4485	0.356	<.001	0.225	0.475
					B	10	3145	0.407	<.001	0.237	0.553
13	confirmation	satisfaction	0.119	.730	A	12	4031	0.483	<.001	0.354	0.594
					B	8	2691	0.449	<.001	0.265	0.601
14	perceived ease of use	satisfaction	1.558	.212	A	6	2485	0.242	<.001	0.156	0.325
					B	4	1689	0.293	<.001	0.222	0.361
15	service quality	satisfaction	0.123	.725	A	5	1691	0.623	<.001	0.223	0.843
					B	4	1492	0.679	<.001	0.201	0.896
16	confirmation	perceived usefulness	1.204	.272	A	12	3809	0.660	<.001	0.488	0.783
					B	8	2469	0.758	<.001	0.605	0.857
17	perceived ease of	perceived	0.060	.807	A	7	2961	0.416	<.001	0.138	0.634

	use	usefulness									
					B	4	1689	0.464	.015	-0.153	0.820
18	service quality	perceived usefulness	0.690	.406	A	3	559	0.512	<.001	0.097	0.775
					B	2	413	0.426	<.001	-0.401	0.870
19	Information quality	perceived usefulness	0.214	.644	A	3	559	0.301	.039	-0.325	0.744
					B	2	413	0.399	.017	-0.949	0.990
20	confirmation	perceived ease of use	0.946	.331	A	4	1760	0.621	<.001	0.421	0.764
					B	2	964	0.678	<.001	0.240	0.887

Note: A: All studies, B: No low-quality studies (Score less than or equal to 10). a: P-value of test for groups difference of combined coefficient. b: P-value of test for significance of combined coefficient.

Supplementary Figure B1. Funnel Plot for all studies reporting on the regression coefficient of satisfaction on continuance intention

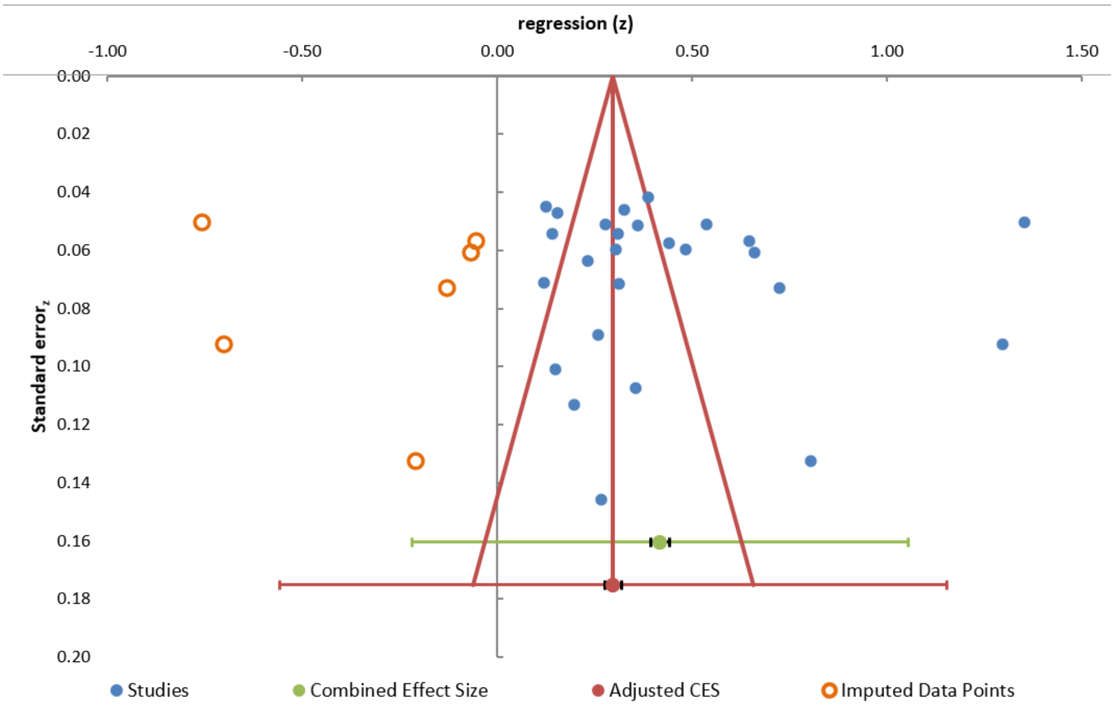

Egger's test for small-study effects:

	Estimate	SE	CI LL	CI UL
Intercept	1.37	3.60	-6.04	8.79
Slope	0.34	0.22	-0.11	0.79

t test	0.38
p-value	0.71

Supplementary Figure B2. Funnel Plot for all studies reporting on the regression coefficient of perceived usefulness on continuance intention

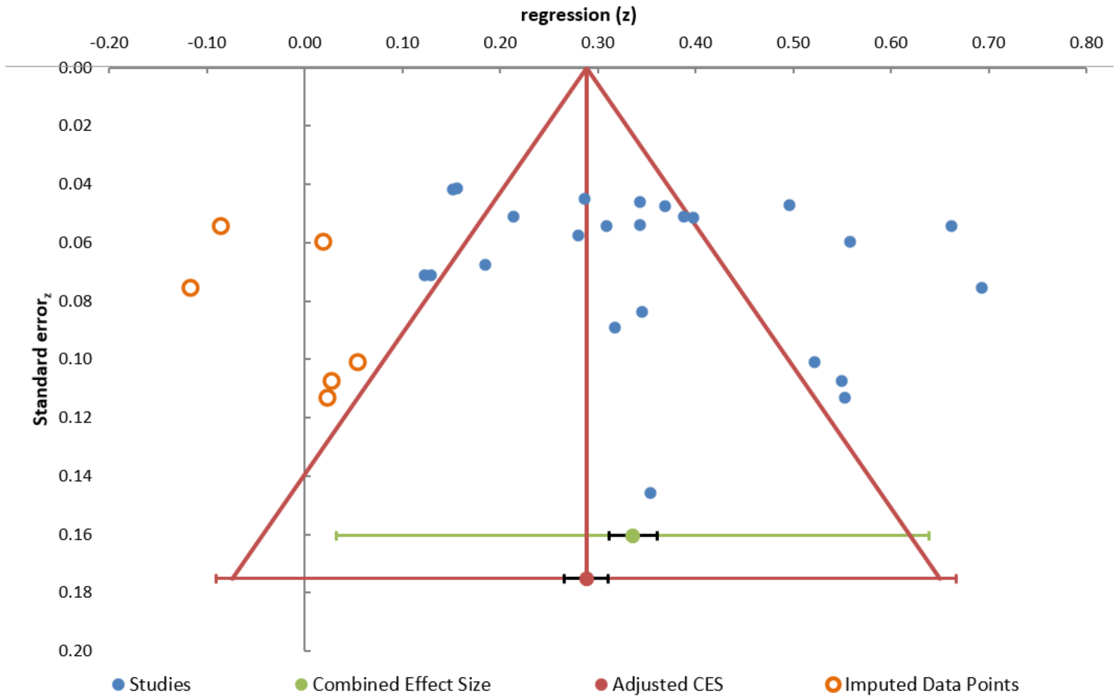

Egger's test for small-study effects:

	Estimate	SE	CI LL	CI UL
Intercept	2.55	1.90	-1.38	6.48
Slope	0.19	0.11	-0.03	0.42

t test	1.34
p-value	0.19

Supplementary Figure B3. Funnel Plot for all studies reporting on the regression coefficient of perceived ease of use on continuance intention

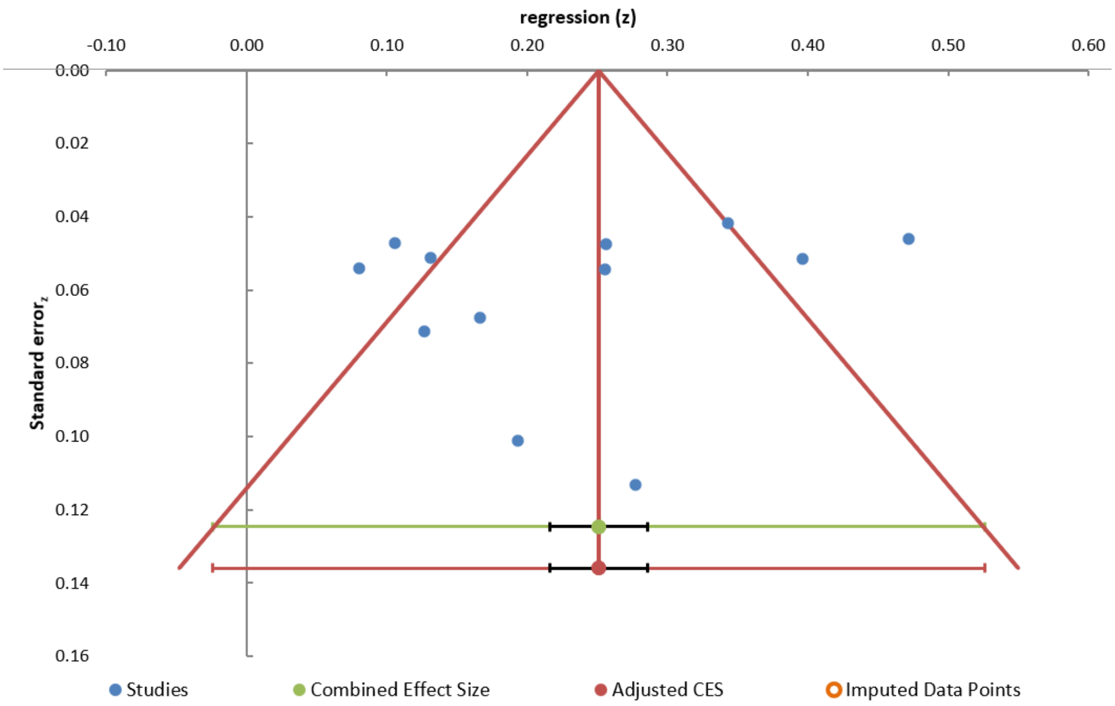

Egger's test for small-study effects:

	Estimate	SE	CI LL	CI UL
Intercept	-2.51	2.86	-8.80	3.78
Slope	0.38	0.16	0.04	0.73

t test	-0.88
p-value	0.40

Supplementary Figure B4. Funnel Plot for all studies reporting on the regression coefficient of trust on continuance intention

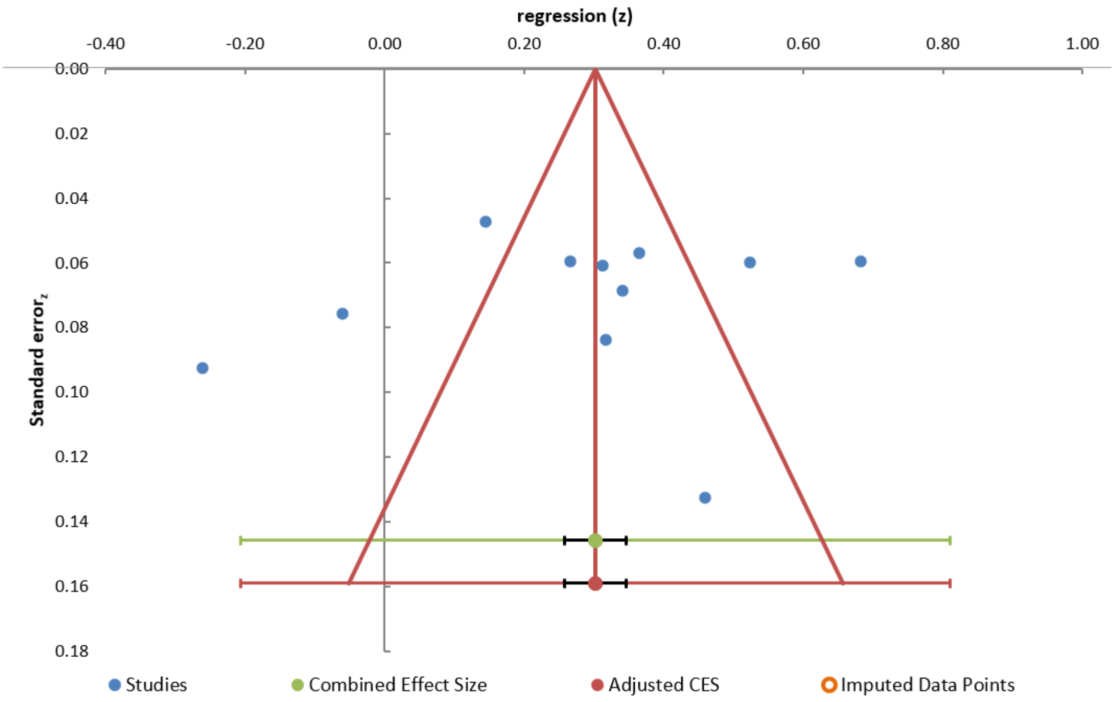

Egger's test for small-study effects:

	Estimate	SE	CI LL	CI UL
Intercept	-2.57	4.78	-13.23	8.09
Slope	0.47	0.31	-0.23	1.16

t test	-0.54
p-value	0.60

Supplementary Figure B5. Funnel Plot for all studies reporting on the regression coefficient of social influence on continuance intention

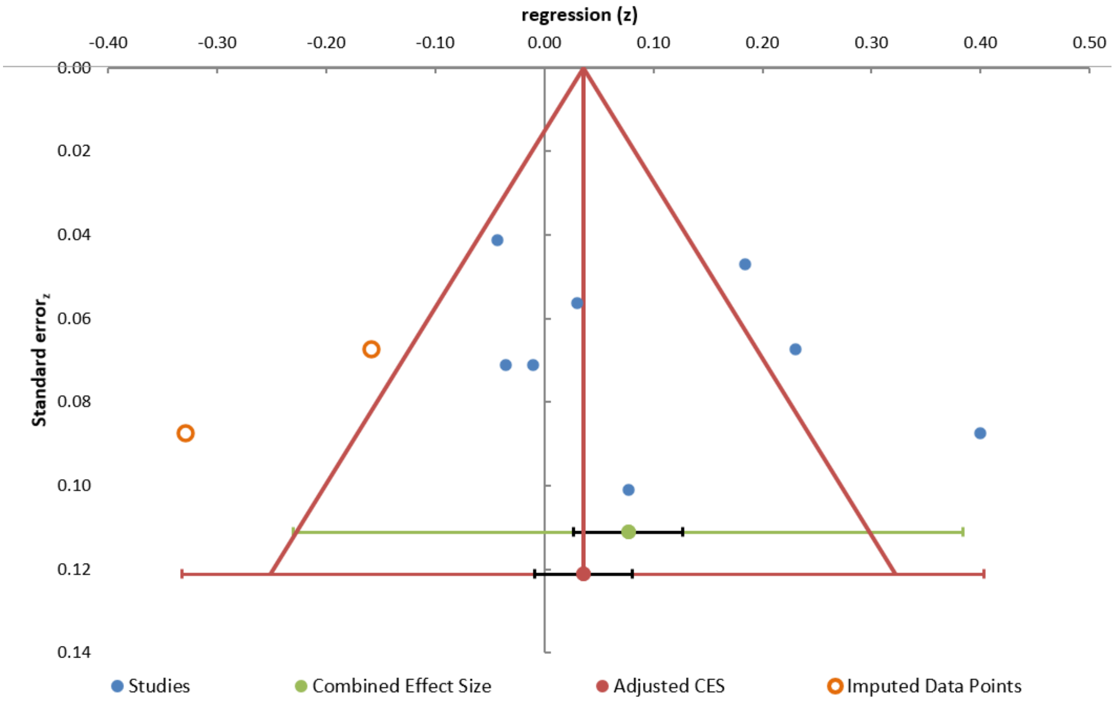

Egger's test for small-study effects:

	Estimate	SE	CI LL	CI UL
Intercept	2.87	2.99	-4.19	9.93
Slope	-0.09	0.18	-0.52	0.34

t test	0.96
p-value	0.37

Supplementary Figure B6. Funnel Plot for all studies reporting on the regression coefficient of performance expectancy on continuance intention

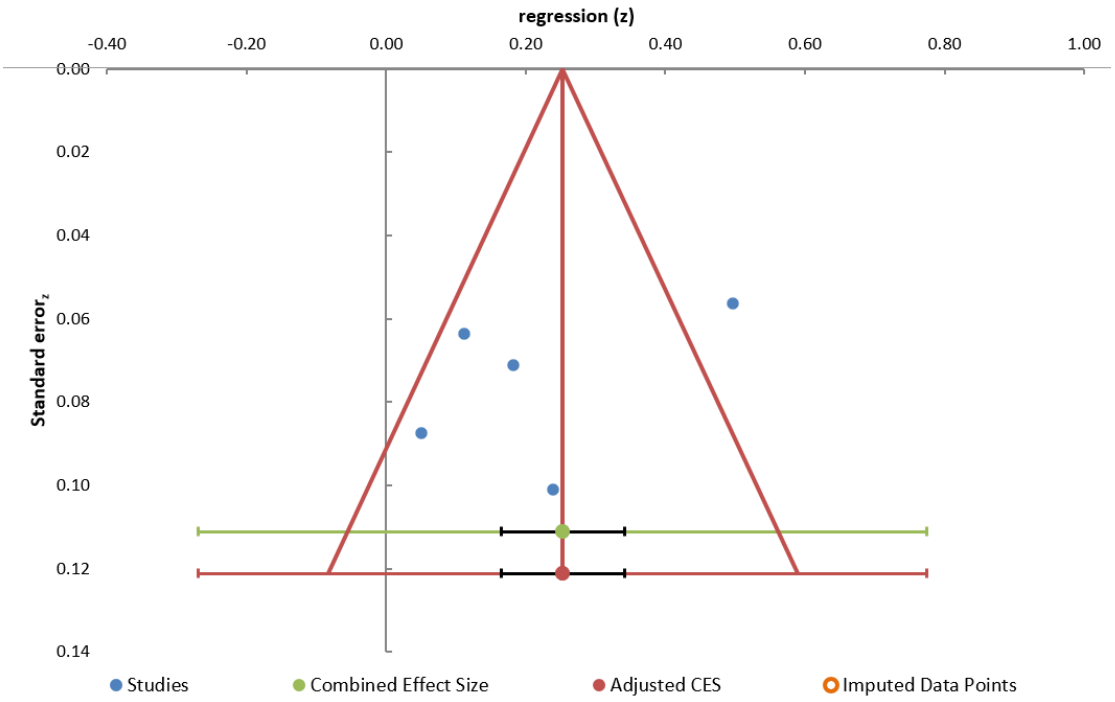

Egger's test for small-study effects:

	Estimate	SE	CI LL	CI UL
Intercept	-6.62	5.93	-23.08	9.83
Slope	0.71	0.42	-0.46	1.88

t test	-1.12
p-value	0.35

Supplementary Figure B7. Funnel Plot for all studies reporting on the regression coefficient of facilitating conditions on continuance intention

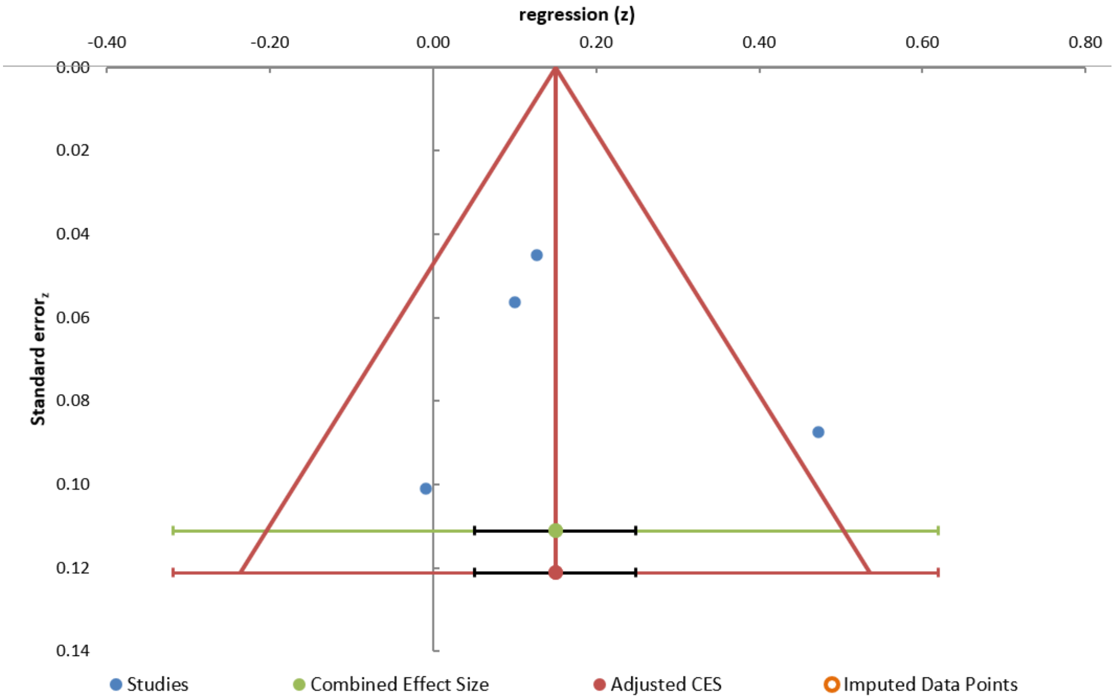

Egger's test for small-study effects:

	Estimate	SE	CI LL	CI UL
Intercept	1.96	4.57	-12.58	16.50
Slope	0.03	0.28	-0.87	0.94

t test	0.43
p-value	0.71

Supplementary Figure B8. Funnel Plot for all studies reporting on the regression coefficient of service quality on continuance intention

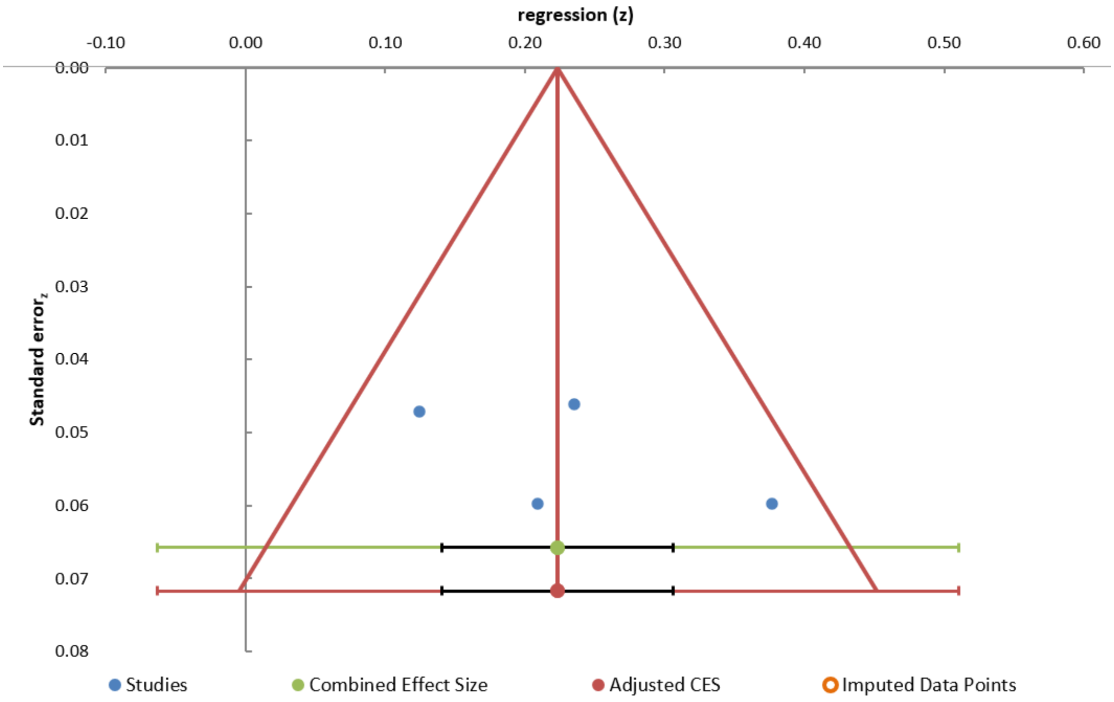

Egger's test for small-study effects:

	Estimate	SE	CI LL	CI UL
Intercept	8.05	7.73	-16.54	32.63
Slope	-0.19	0.40	-1.47	1.09

t test	1.04
p-value	0.41

Supplementary Figure B9. Funnel Plot for all studies reporting on the regression coefficient of attitude on continuance intention

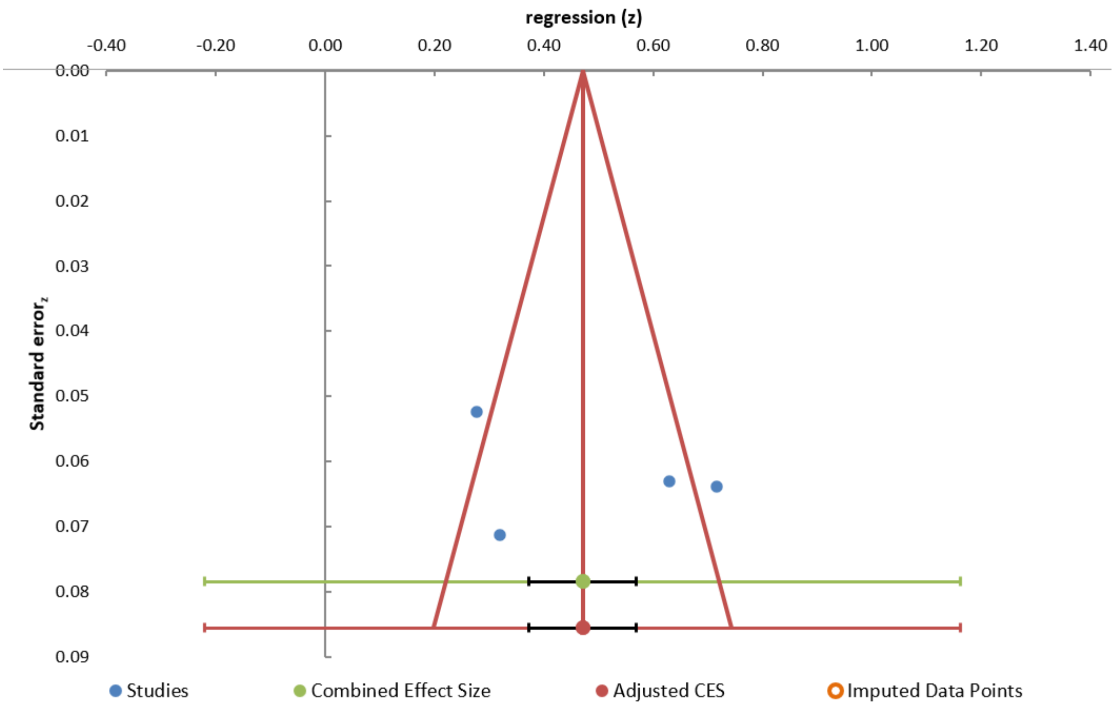

Egger's test for small-study effects:

	Estimate	SE	CI LL	CI UL
Intercept	10.80	18.07	-46.70	68.30
Slope	-0.19	1.11	-3.72	3.34

t test	0.60
p-value	0.61

Supplementary Figure B10. Funnel Plot for all studies reporting on the regression coefficient of habit on continuance intention

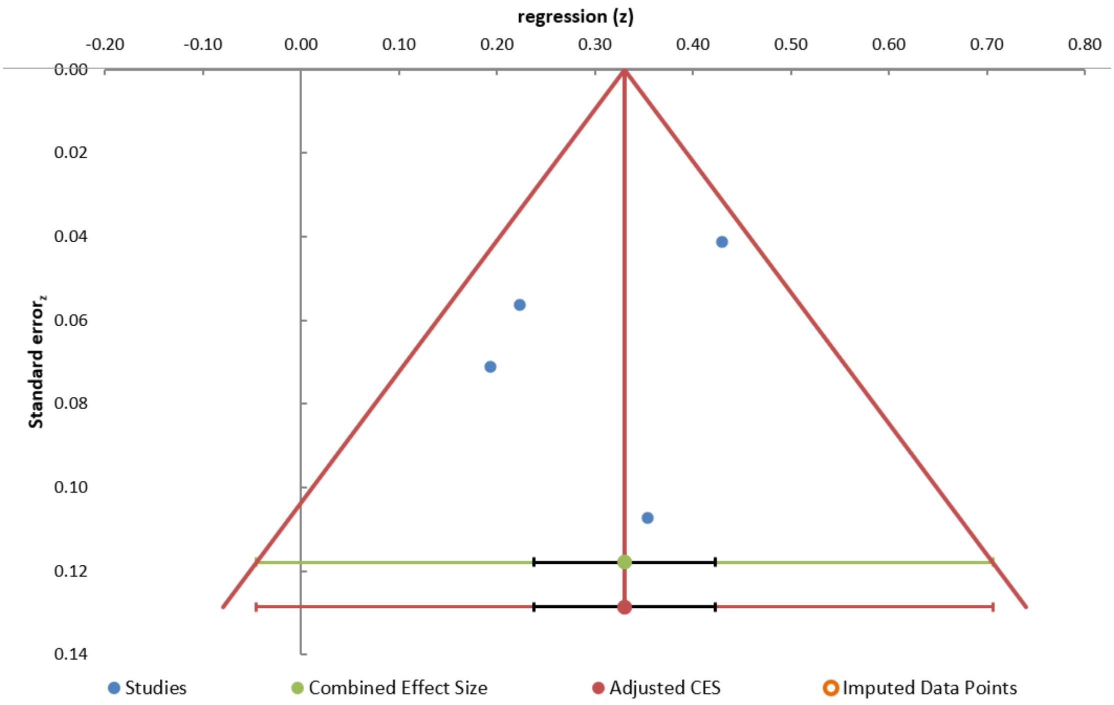

Egger's test for small-study effects:

	Estimate	SE	CI LL	CI UL
Intercept	-2.95	3.47	-14.00	8.11
Slope	0.49	0.20	-0.15	1.13

t test	-0.85
p-value	0.49

Supplementary Figure B11. Funnel Plot for all studies reporting on the regression coefficient of innovation on continuance intention

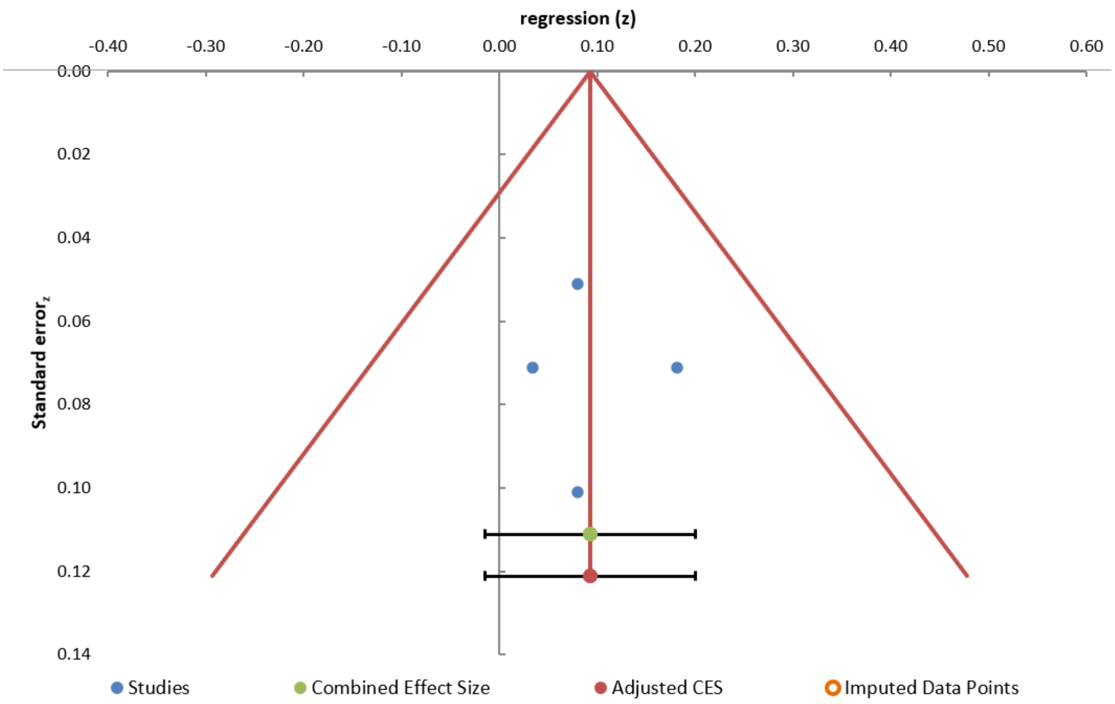

Egger's test for small-study effects:

	Estimate	SE	CI LL	CI UL
Intercept	0.28	2.32	-7.09	7.65
Slope	0.07	0.16	-0.42	0.57

t test	0.12
p-value	0.92

Supplementary Figure B12. Funnel Plot for all studies reporting on the regression coefficient of self-efficacy on continuance intention

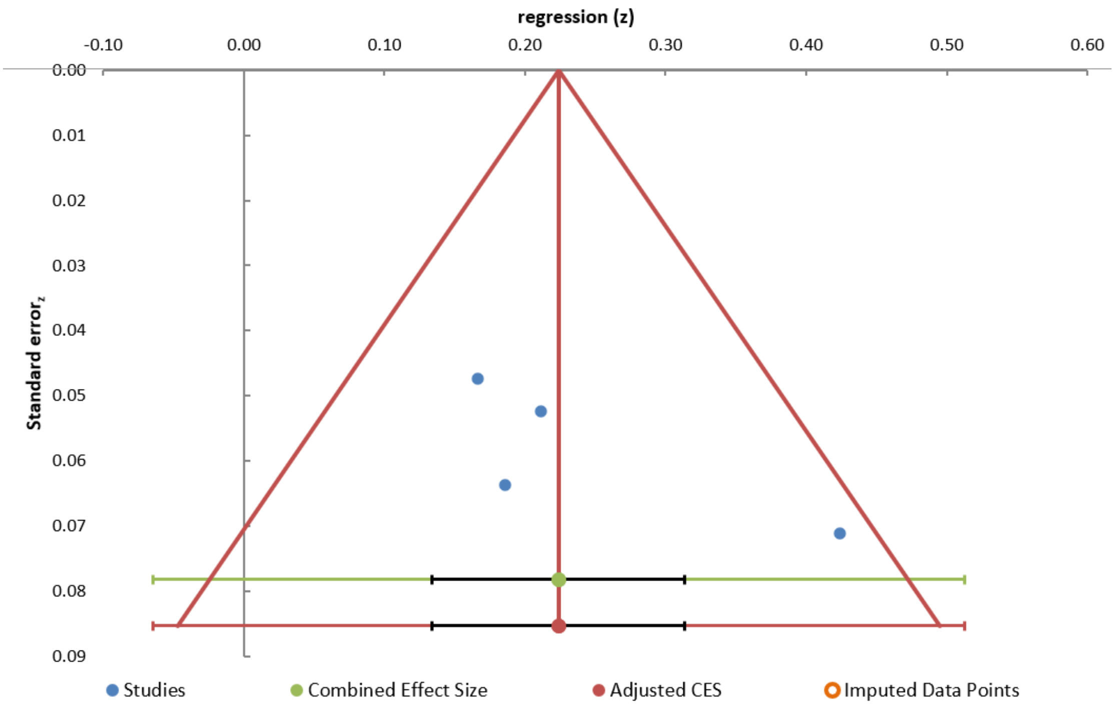

Egger's test for small-study effects:

	Estimate	SE	CI LL	CI UL
Intercept	7.83	4.42	-6.23	21.89
Slope	-0.21	0.25	-1.01	0.58

t test	1.77
p-value	0.22

Supplementary Figure B13. Funnel Plot for all studies reporting on the regression coefficient of perceived enjoyment on continuance intention

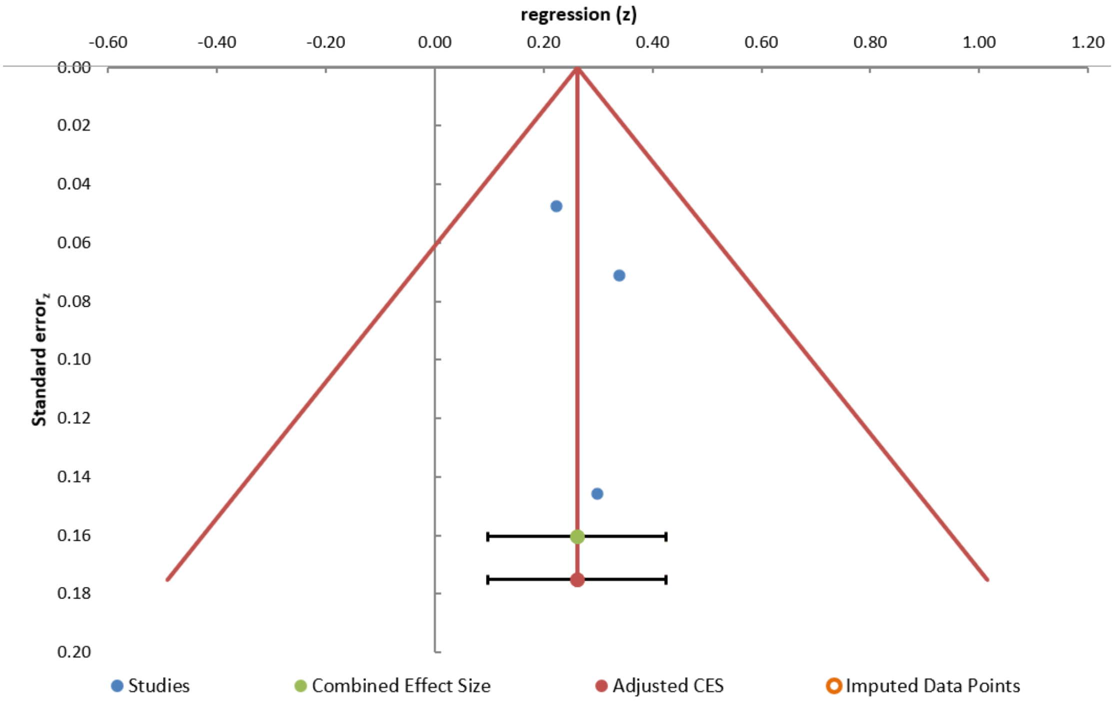

Egger's test for small-study effects:

	Estimate	SE	CI LL	CI UL
Intercept	1.23	1.70	-6.07	8.52
Slope	0.19	0.11	-0.29	0.67

t test	0.72
p-value	0.60

Supplementary Figure B14. Funnel Plot for all studies reporting on the regression coefficient of perceived quality of health life on continuance intention

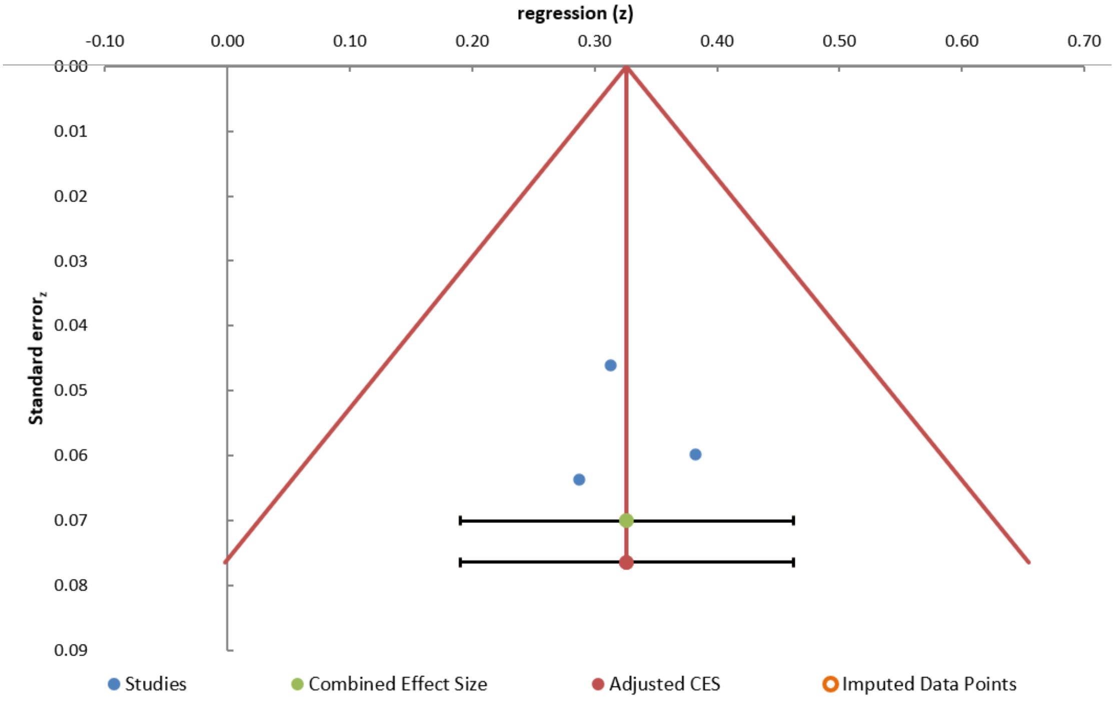

Egger's test for small-study effects:

	Estimate	SE	CI LL	CI UL
Intercept	0.80	4.61	-19.05	20.65
Slope	0.28	0.25	-0.81	1.37

t test	0.17
p-value	0.89

Supplementary Figure B15. Funnel Plot for all studies reporting on the regression coefficient of perceived risk on continuance intention

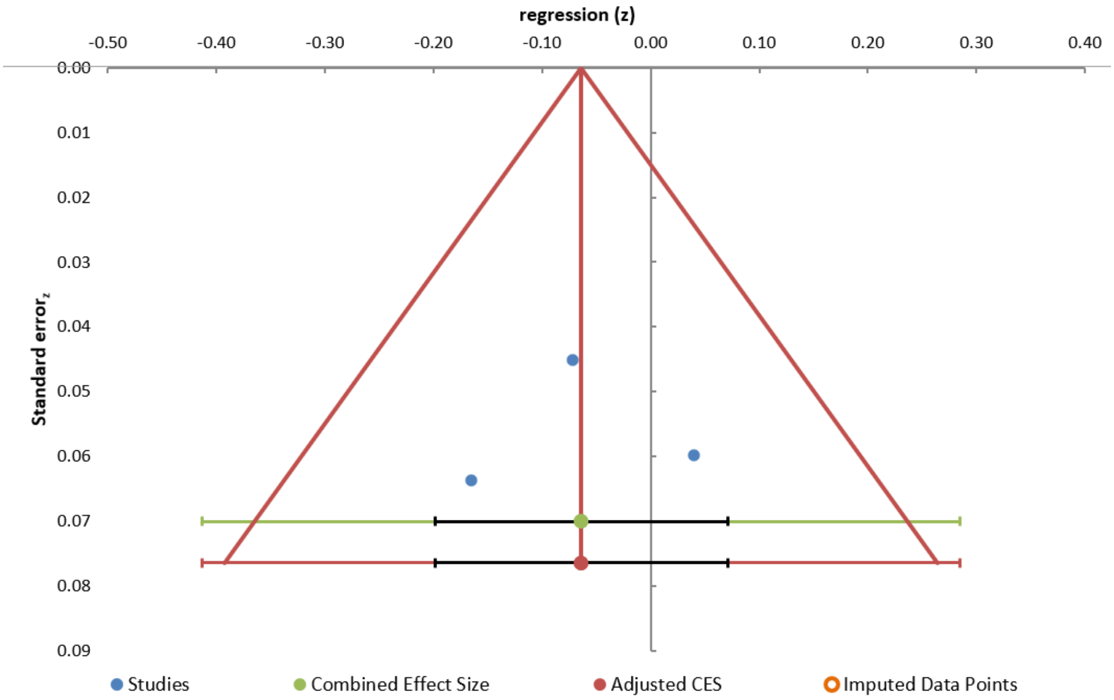

Egger's test for small-study effects:

	Estimate	SE	CI LL	CI UL
Intercept	-0.54	8.89	-38.78	37.71
Slope	-0.03	0.48	-2.11	2.04

t test	-0.06
p-value	0.96

Supplementary Figure B16. Funnel Plot for all studies reporting on the regression coefficient of perceived value on continuance intention

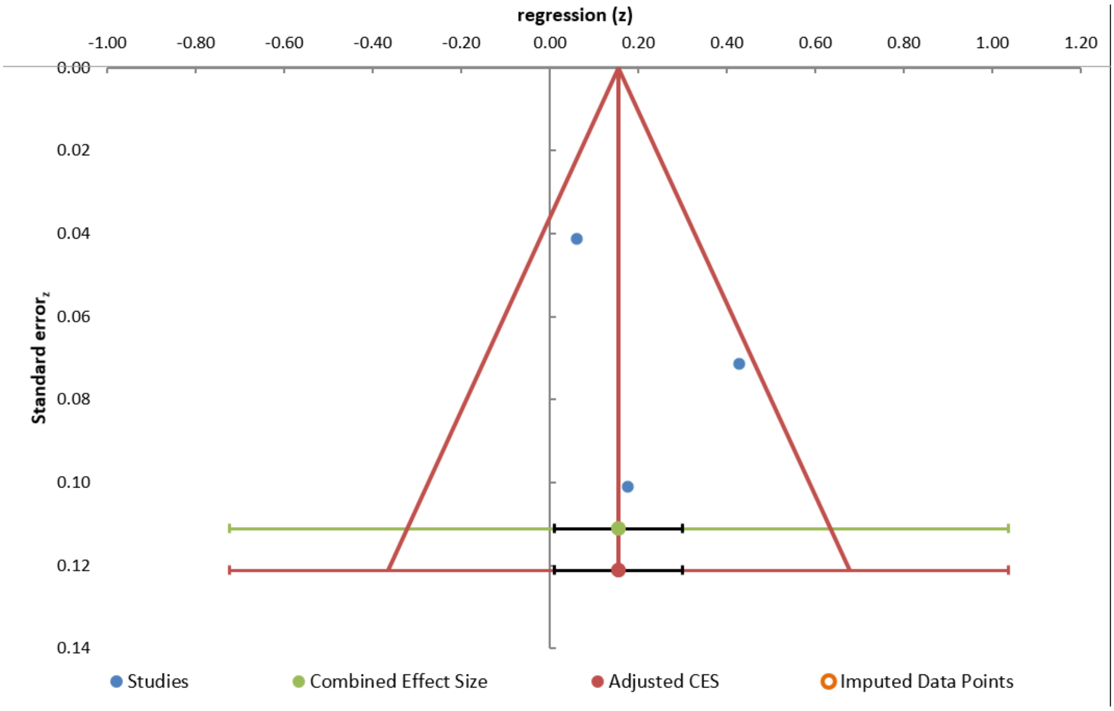

Egger's test for small-study effects:

	Estimate	SE	CI LL	CI UL
Intercept	4.67	5.60	-19.43	28.77
Slope	-0.10	0.33	-1.51	1.31

t test	0.83
p-value	0.56

Supplementary Figure B17. Funnel Plot for all studies reporting on the regression coefficient of health empowerment on continuance intention

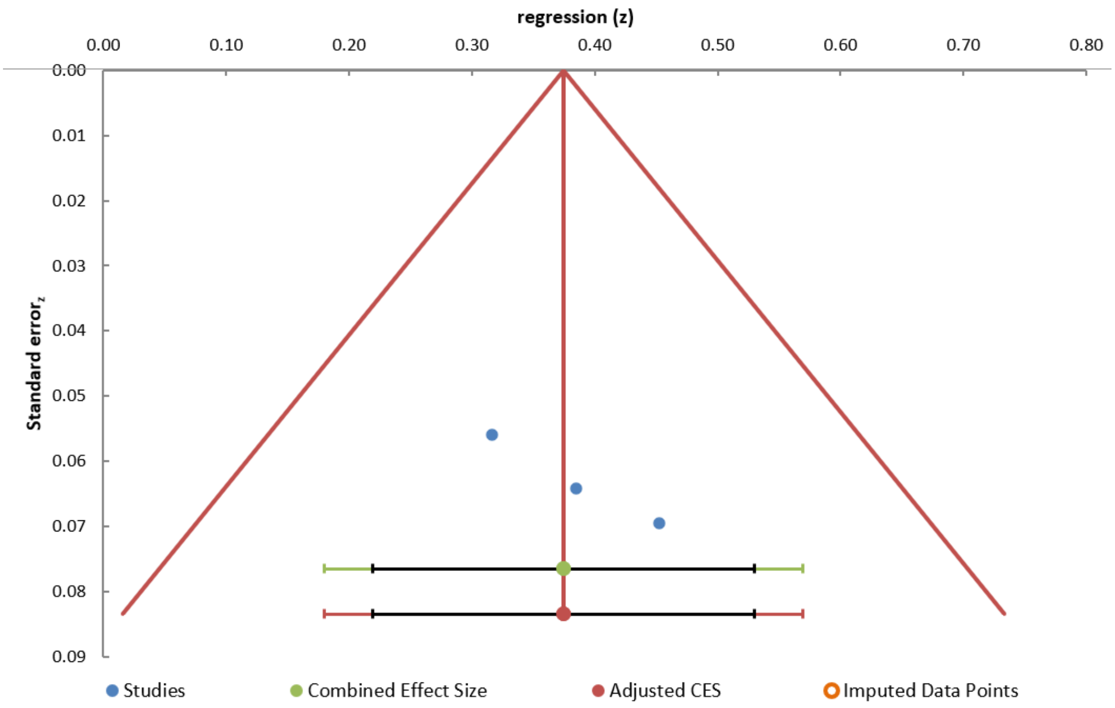

Egger's test for small-study effects:

	Estimate	SE	CI LL	CI UL
Intercept	9.76	1.11	4.99	14.53
Slope	-0.23	0.07	-0.53	0.07

t test	8.80
p-value	0.07

Supplementary Figure B18. Funnel Plot for all studies reporting on the regression coefficient of engagement on continuance intention

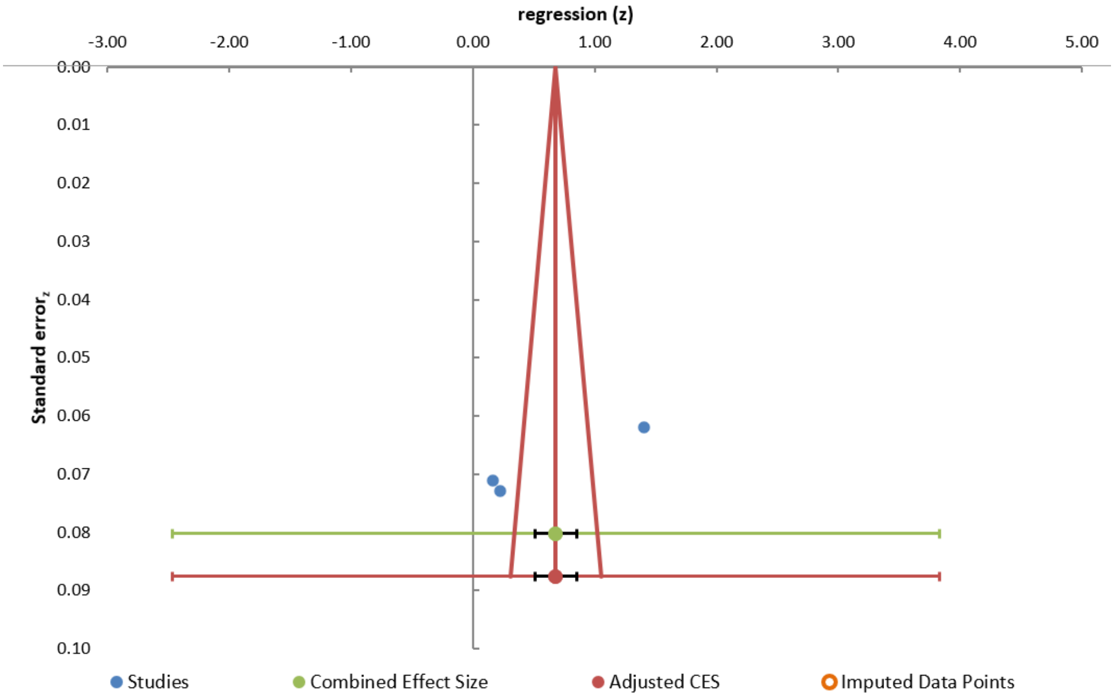

Egger's test for small-study effects:

	Estimate	SE	CI LL	CI UL
Intercept	-118.79	22.46	-215.43	-22.15
Slope	8.76	1.53	2.17	15.34

t test	-5.29
p-value	0.12

Supplementary Figure B19. Funnel Plot for all studies reporting on the regression coefficient of function gratification on continuance intention

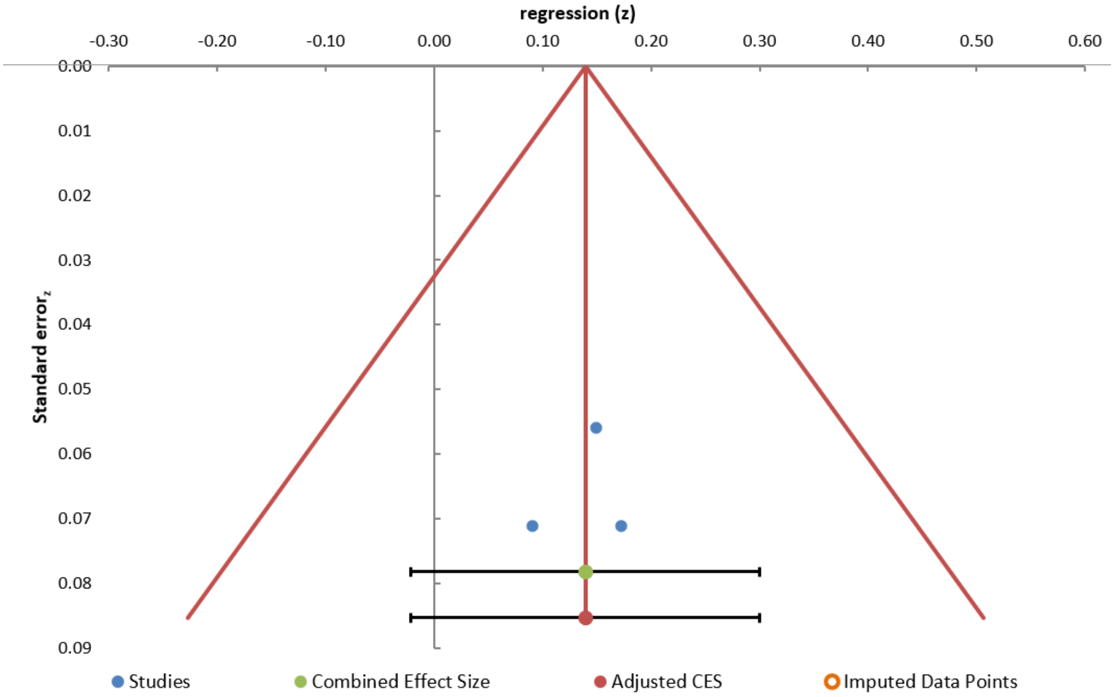

Egger's test for small-study effects:

	Estimate	SE	CI LL	CI UL
Intercept	-1.16	4.07	-18.66	16.33
Slope	0.21	0.26	-0.92	1.35

t test	-0.29
p-value	0.82

Supplementary Figure B20. Funnel Plot for all studies reporting on the regression coefficient of perceived usefulness on satisfaction

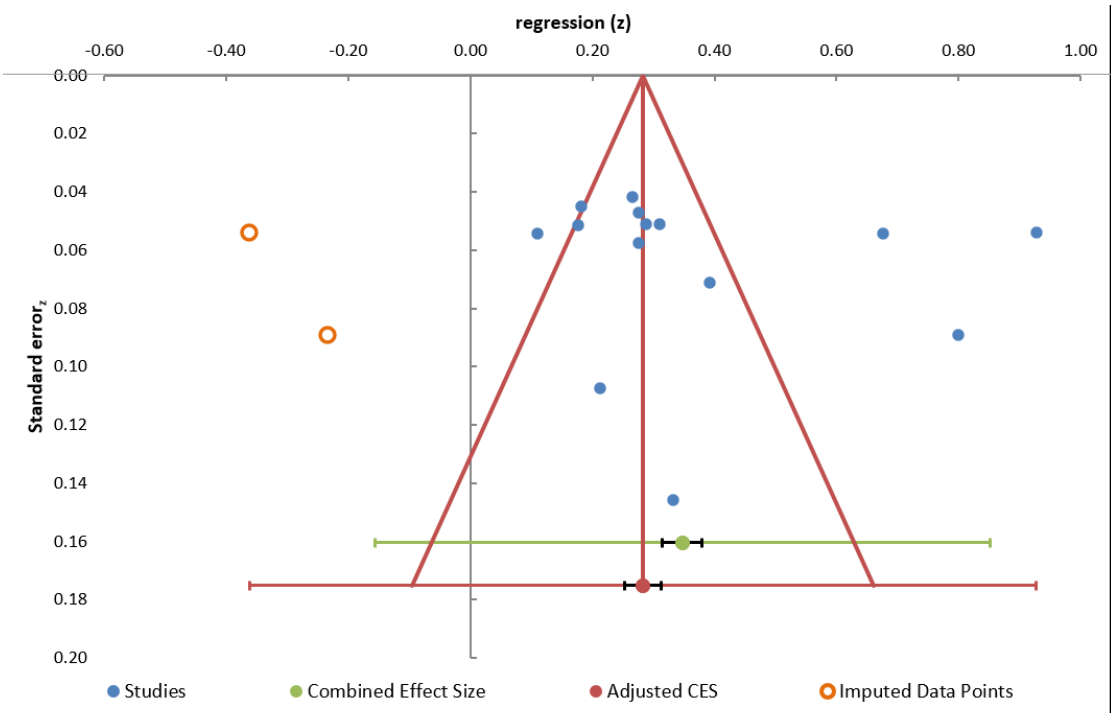

Egger's test for small-study effects:

	Estimate	SE	CI LL	CI UL
Intercept	3.14	4.28	-6.11	12.39
Slope	0.18	0.24	-0.34	0.70

t test	0.73
p-value	0.48

Supplementary Figure B21. Funnel Plot for all studies reporting on the regression coefficient of confirmation on satisfaction

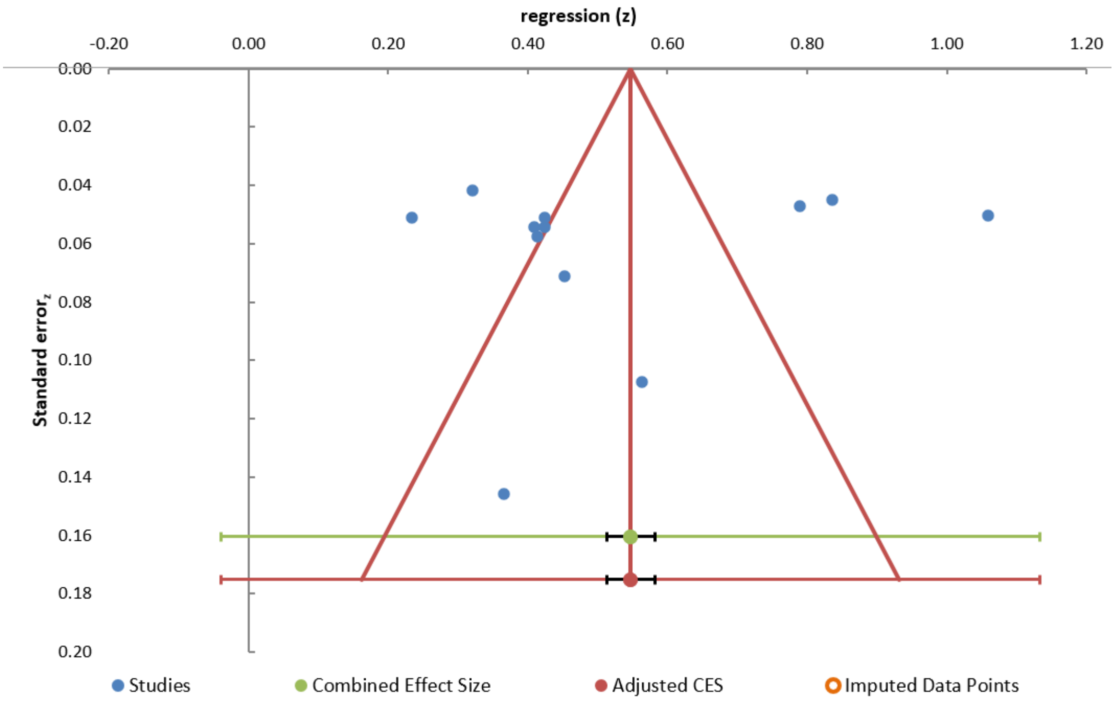

Egger's test for small-study effects:

	Estimate	SE	CI LL	CI UL
Intercept	-2.18	5.51	-14.32	9.96
Slope	0.66	0.30	0.00	1.33

t test	-0.40
p-value	0.70

Supplementary Figure B22. Funnel Plot for all studies reporting on the regression coefficient of perceived ease of use on satisfaction

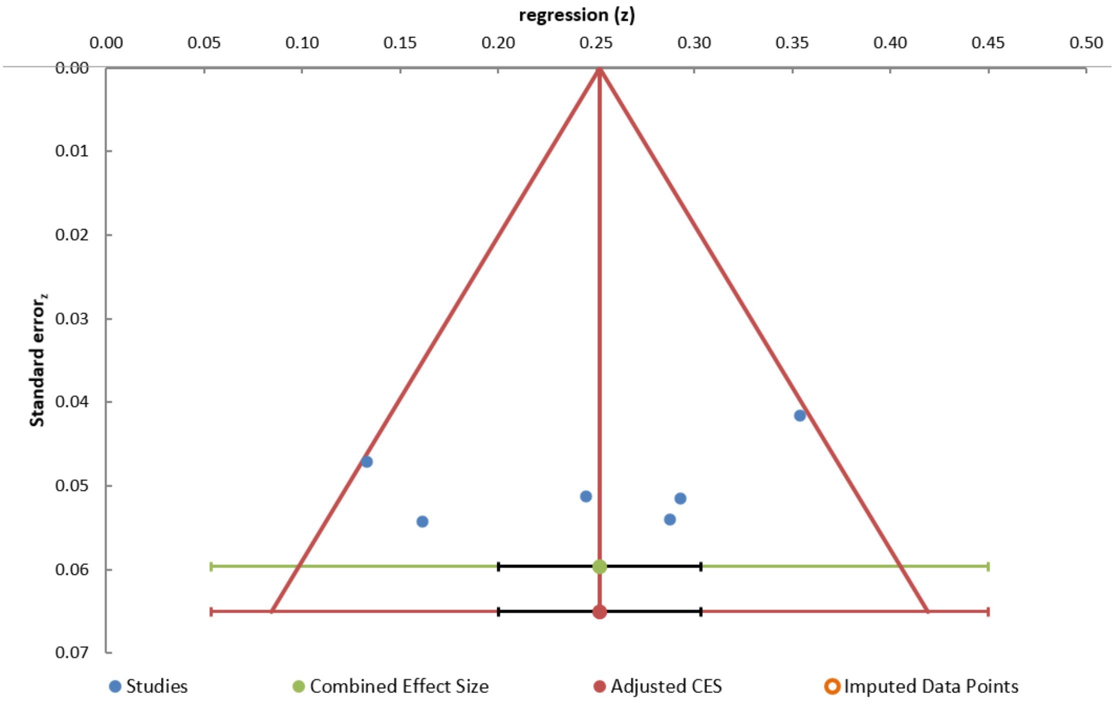

Egger's test for small-study effects:

	Estimate	SE	CI LL	CI UL
Intercept	-7.27	7.79	-27.30	12.75
Slope	0.61	0.38	-0.38	1.60

t test	-0.93
p-value	0.40

Supplementary Figure B23. Funnel Plot for all studies reporting on the regression coefficient of service quality on satisfaction

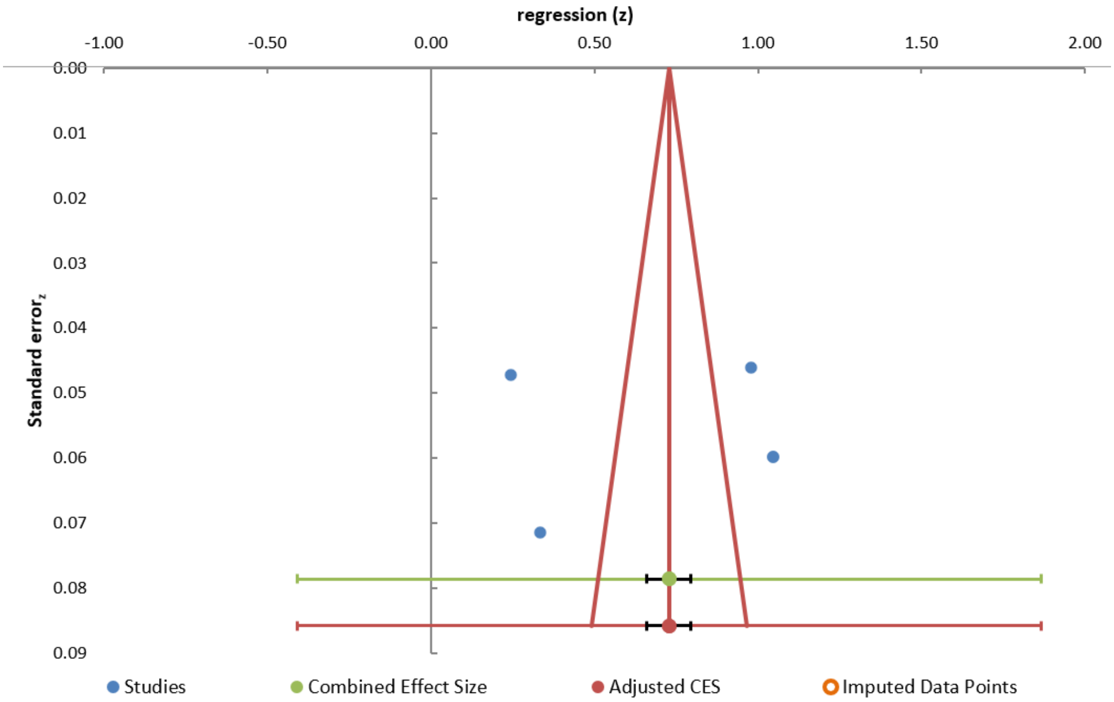

Egger's test for small-study effects:

	Estimate	SE	CI LL	CI UL
Intercept	1.90	24.02	-64.78	68.58
Slope	0.63	1.31	-3.02	4.27

t test	0.08
p-value	0.94

Supplementary Figure B24. Funnel Plot for all studies reporting on the regression coefficient of confirmation on perceived usefulness

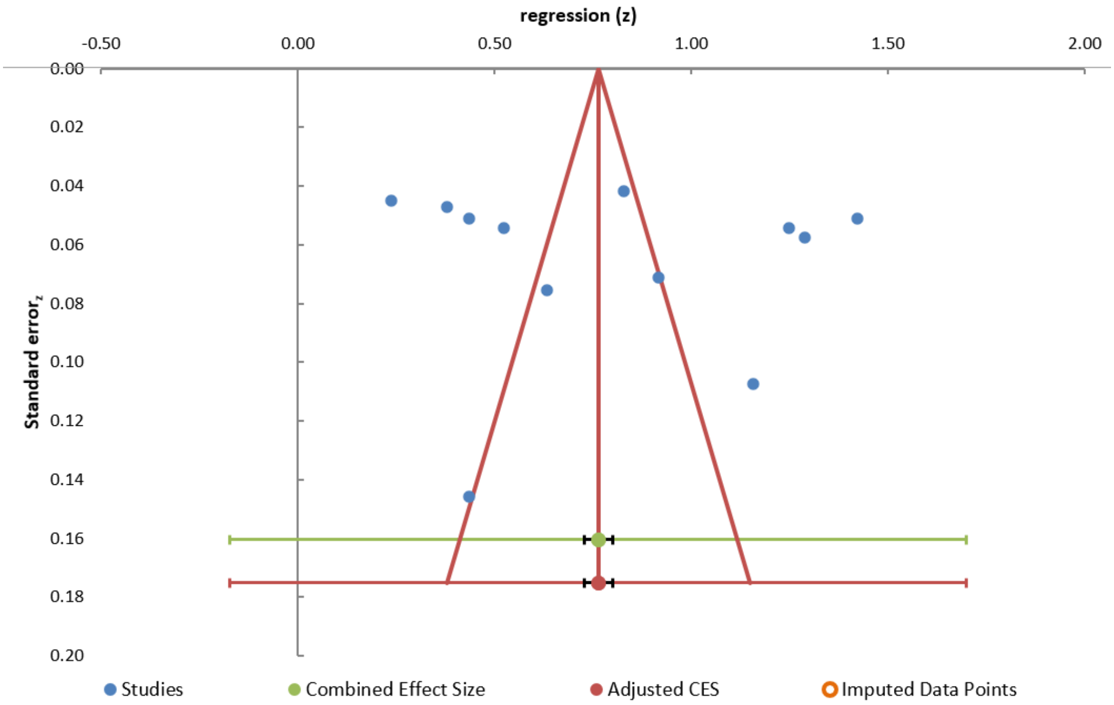

Egger's test for small-study effects:

	Estimate	SE	CI LL	CI UL
Intercept	3.92	8.04	-13.76	21.61
Slope	0.55	0.45	-0.45	1.55

t test	0.49
p-value	0.64

Supplementary Figure B25. Funnel Plot for all studies reporting on the regression coefficient of perceived ease of use on perceived usefulness

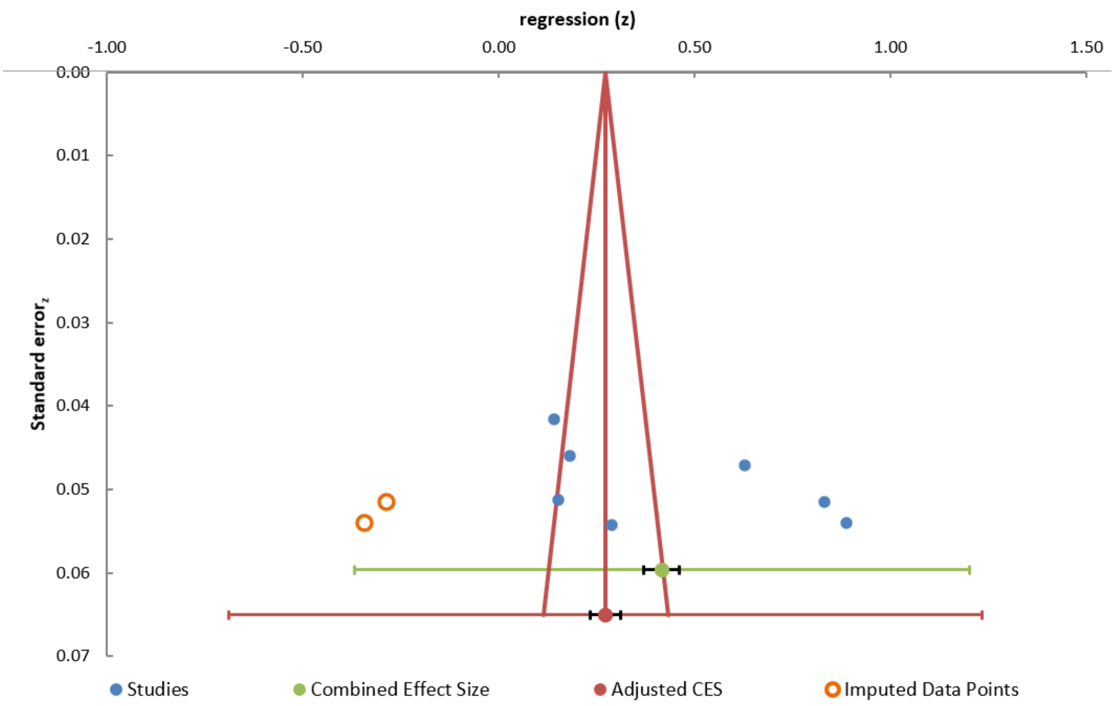

Egger's test for small-study effects:

	Estimate	SE	CI LL	CI UL
Intercept	35.31	25.34	-26.70	97.32
Slope	-1.30	1.24	-4.33	1.73

t test	1.39
p-value	0.22

Supplementary Figure B26. Funnel Plot for all studies reporting on the regression coefficient of service quality on perceived usefulness

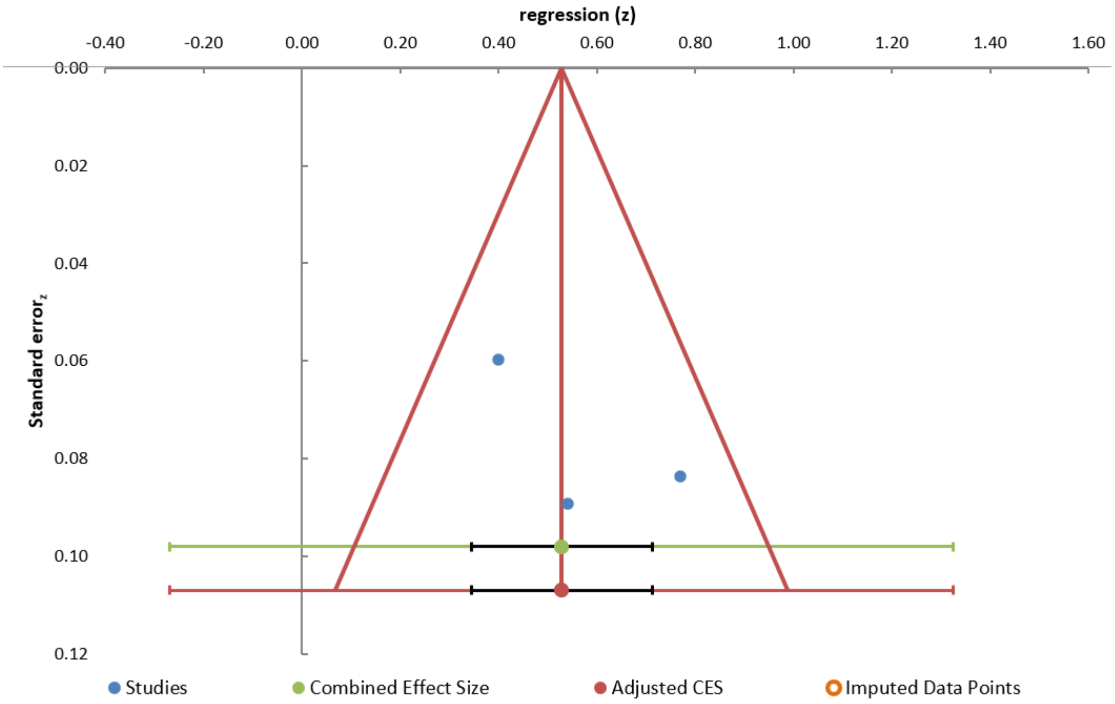

Egger's test for small-study effects:

	Estimate	SE	CI LL	CI UL
Intercept	8.89	7.31	-22.55	40.33
Slope	-0.12	0.54	-2.44	2.21

t test	1.22
p-value	0.44

Supplementary Figure B27. Funnel Plot for all studies reporting on the regression coefficient of information quality on perceived usefulness

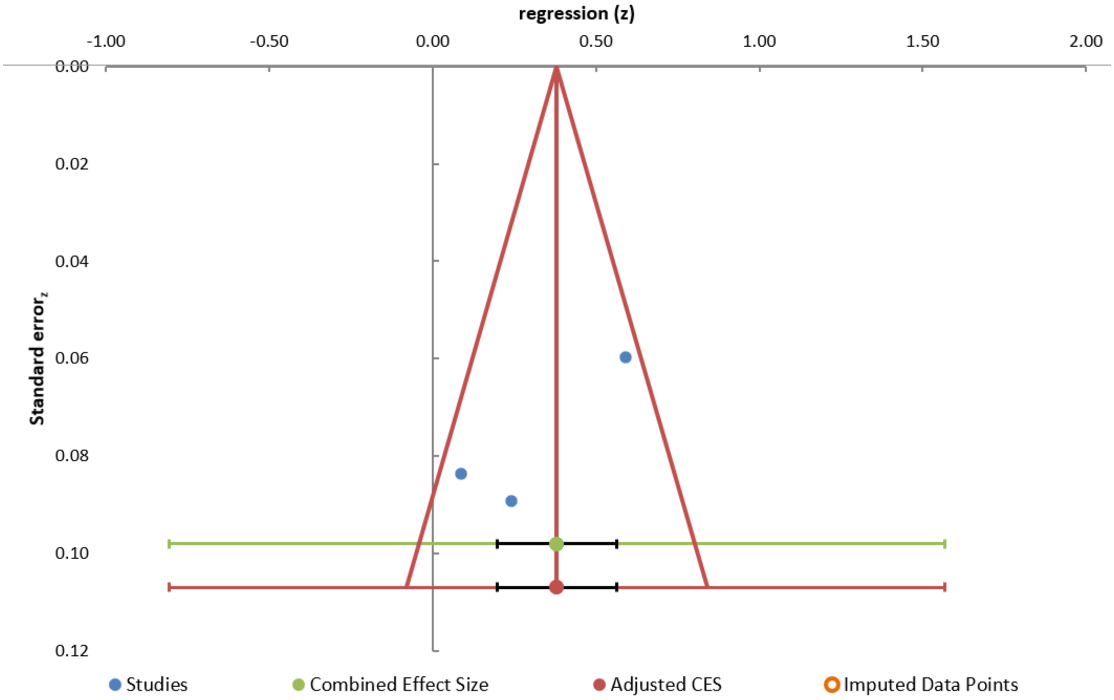

Egger's test for small-study effects:

	Estimate	SE	CI LL	CI UL
Intercept	−15.44	6.22	−42.22	11.33
Slope	1.50	0.46	−0.48	3.48

t test	−2.48
p-value	0.24

Supplementary Figure B28. Funnel Plot for all studies reporting on the regression coefficient of confirmation on perceived ease of use

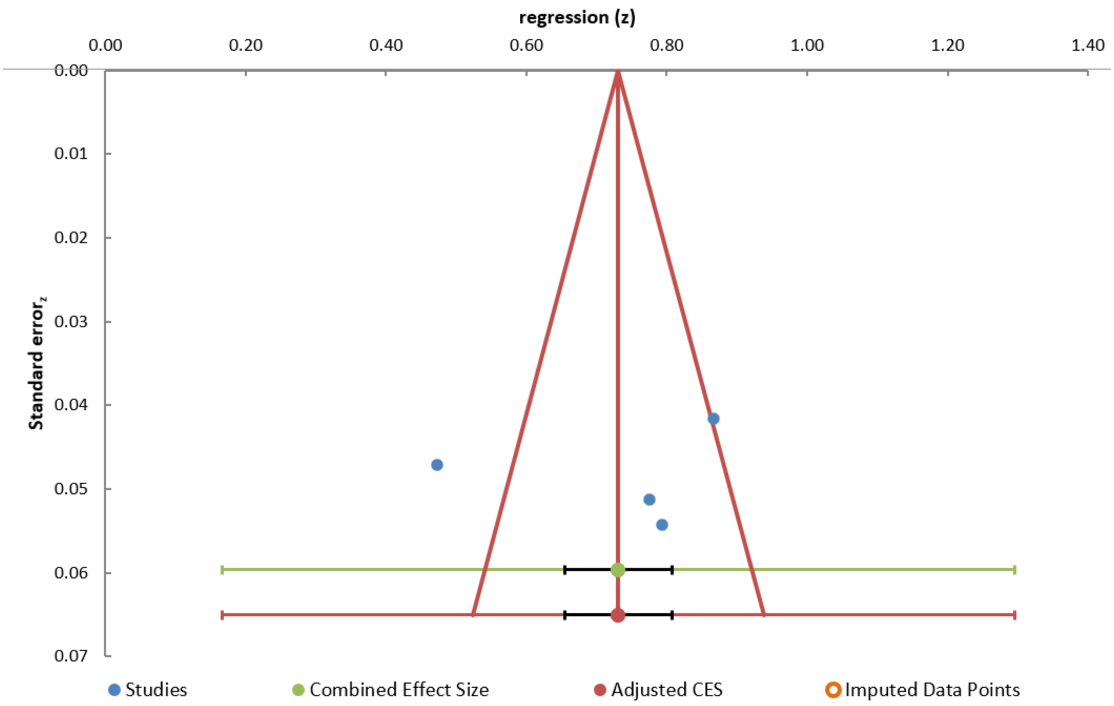

Egger's test for small-study effects:

	Estimate	SE	CI LL	CI UL
Intercept	-5.30	22.67	-77.43	66.84
Slope	0.98	1.08	-2.47	4.43

t test	-0.23
p-value	0.84

Supplementary Table 3. PRISMA checklist

Section and Topic	Item #	Checklist item	Location where item is reported
**TITLE**			
Title	1	Identify the report as a systematic review.	P1
**ABSTRACT**			
Abstract	2	See the PRISMA 2020 for Abstracts checklist.	P3
**INTRODUCTION**			
Rationale	3	Describe the rationale for the review in the context of existing knowledge.	P4-6
Objectives	4	Provide an explicit statement of the objective(s) or question(s) the review addresses.	P7
**METHODS**			
Eligibility criteria	5	Specify the inclusion and exclusion criteria for the review and how studies were grouped for the syntheses.	P22
Information sources	6	Specify all databases, registers, websites, organisations, reference lists and other sources searched or consulted to identify studies. Specify the date when each source was last searched or consulted.	P21
Search strategy	7	Present the full search strategies for all databases, registers and websites, including any filters and limits used.	Supplementary Table 4
Selection process	8	Specify the methods used to decide whether a study met the inclusion criteria of the review, including how many reviewers screened each record and each report retrieved, whether they worked independently, and if applicable, details of automation tools used in the process.	P22-23
Data collection process	9	Specify the methods used to collect data from reports, including how many reviewers collected data from each report, whether they worked independently, any processes for obtaining or confirming data from study investigators, and if applicable, details of automation tools used in the process.	P23
Data items	10a	List and define all outcomes for which data were sought. Specify whether all results that were compatible with each outcome domain in each study were sought (e.g. for all measures, time points, analyses), and if not, the methods used to decide which results to collect.	P23

Section and Topic	Item #	Checklist item	Location where item is reported
	10b	List and define all other variables for which data were sought (e.g. participant and intervention characteristics, funding sources). Describe any assumptions made about any missing or unclear information.	P23
Study risk of bias assessment	11	Specify the methods used to assess risk of bias in the included studies, including details of the tool(s) used, how many reviewers assessed each study and whether they worked independently, and if applicable, details of automation tools used in the process.	P24
Effect measures	12	Specify for each outcome the effect measure(s) (e.g. risk ratio, mean difference) used in the synthesis or presentation of results.	P23
Synthesis methods	13a	Describe the processes used to decide which studies were eligible for each synthesis (e.g. tabulating the study intervention characteristics and comparing against the planned groups for each synthesis (item #5)).	P25-26
	13b	Describe any methods required to prepare the data for presentation or synthesis, such as handling of missing summary statistics, or data conversions.	P24
	13c	Describe any methods used to tabulate or visually display results of individual studies and syntheses.	n/a
	13d	Describe any methods used to synthesize results and provide a rationale for the choice(s). If meta-analysis was performed, describe the model(s), method(s) to identify the presence and extent of statistical heterogeneity, and software package(s) used.	P24
	13e	Describe any methods used to explore possible causes of heterogeneity among study results (e.g. subgroup analysis, meta-regression).	P25
	13f	Describe any sensitivity analyses conducted to assess robustness of the synthesized results.	P12
Reporting bias assessment	14	Describe any methods used to assess risk of bias due to missing results in a synthesis (arising from reporting biases).	P24
Certainty assessment	15	Describe any methods used to assess certainty (or confidence) in the body of evidence for an outcome.	P23
**RESULTS**			
Study selection	16a	Describe the results of the search and selection process, from the number of records identified in the search to the number of studies included in the review, ideally using a flow diagram.	P8

Section and Topic	Item #	Checklist item	Location where item is reported
	16b	Cite studies that might appear to meet the inclusion criteria, but which were excluded, and explain why they were excluded.	Figure 1
Study characteristics	17	Cite each included study and present its characteristics.	Table 1
Risk of bias in studies	18	Present assessments of risk of bias for each included study.	P12
Results of individual studies	19	For all outcomes, present, for each study: (a) summary statistics for each group (where appropriate) and (b) an effect estimate and its precision (e.g. confidence/credible interval), ideally using structured tables or plots.	Supplementary Figure A1-A28
Results of syntheses	20a	For each synthesis, briefly summarise the characteristics and risk of bias among contributing studies.	Supplementary Figure B1-B28
	20b	Present results of all statistical syntheses conducted. If meta-analysis was done, present for each the summary estimate and its precision (e.g. confidence/credible interval) and measures of statistical heterogeneity. If comparing groups, describe the direction of the effect.	Figure 1
	20c	Present results of all investigations of possible causes of heterogeneity among study results.	P11-12
	20d	Present results of all sensitivity analyses conducted to assess the robustness of the synthesized results.	P12
Reporting biases	21	Present assessments of risk of bias due to missing results (arising from reporting biases) for each synthesis assessed.	P12
Certainty of evidence	22	Present assessments of certainty (or confidence) in the body of evidence for each outcome assessed.	Figure 1
**DISCUSSION**			
Discussion	23a	Provide a general interpretation of the results in the context of other evidence.	P12-19
	23b	Discuss any limitations of the evidence included in the review.	P19-20
	23c	Discuss any limitations of the review processes used.	P19-20

Section and Topic	Item #	Checklist item	Location where item is reported
	23d	Discuss implications of the results for practice, policy, and future research.	P19-20
**OTHER INFORMATION**			
Registration and protocol	24a	Provide registration information for the review, including register name and registration number, or state that the review was not registered.	P26
	24b	Indicate where the review protocol can be accessed, or state that a protocol was not prepared.	n/a
	24c	Describe and explain any amendments to information provided at registration or in the protocol.	n/a
Support	25	Describe sources of financial or non-financial support for the review, and the role of the funders or sponsors in the review.	P26
Competing interests	26	Declare any competing interests of review authors.	P27
Availability of data, code and other materials	27	Report which of the following are publicly available and where they can be found: template data collection forms; data extracted from included studies; data used for all analyses; analytic code; any other materials used in the review.	P26

Supplementary Table 4. Query strategy and results

Databases	Query	Results	Query time
PubMed	((Telemedicine[MeSH Terms]) OR (mHealth[Title/Abstract]) OR ("m-Health"[Title/Abstract]) OR ("mobile health "[Title/Abstract]) OR ("Telemedicine"[Title/Abstract]) OR ("Telehealth "[Title/Abstract]) OR ("Tele-health"[Title/Abstract]) OR ("ehealth"[Title/Abstract]) OR ("e-health"[Title/Abstract]) OR ("electronic health"[Title/Abstract]) OR (“uHealth” [Title/Abstract]) OR (“ubiquitous health” [Title/Abstract]) OR ("digital medical "[Title/Abstract]) OR ("digital medicine"[Title/Abstract]) OR ("digital health"[Title/Abstract]) OR (("application*"[Title/Abstract] OR "app"[Title/Abstract] OR "apps"[Title/Abstract]) AND (health*[Title/Abstract] OR health[MeSH Terms])))) AND ((("post-adoption"[Title/Abstract]) OR ("post-adoptive"[Title/Abstract]) OR ("continuance" [Title/Abstract]) OR ("continue to use" [Title/Abstract]) OR ("continue using" [Title/Abstract]) OR ("continued" [Title/Abstract]) OR ("continuous" [Title/Abstract]) OR ("sustained"[Title/Abstract]) OR ("stickiness"[Title/Abstract])) AND ("intention"[Title/Abstract]))	143	2021.10.08
WOS core collection	TS=((mHealth OR "m-Health" OR "mobile health" OR "Telemedicine" OR "Telehealth" OR " Tele-health" OR "ehealth " OR "e-health" OR "electronic health" OR “uHealth” OR “ubiquitous health” OR "digital medical" OR "digital medicine" OR "digital health") OR (("application*" OR "app" OR "apps") AND health*)) AND ((TS=("post-adoption" OR "post-adoptive " OR "continuance" OR "continue to use" OR "continue using" OR "continued" OR "continuous" OR "sustained" OR "stickiness")) AND TS=(intention))	263	2021.10.08
EI	("mHealth" WN KY OR "m-health" WN KY OR " mobile health" WN KY OR "Telemedicine" WN KY OR "Telehealth" WN KY OR "Tele-health" WN KY OR "ehealth" WN KY OR "e-health" WN KY OR "electronic health" WN KY OR “uHealth” WN KY OR “ubiquitous health” WN KY OR "digital medical" WN KY OR "digital medicine" WN KY OR "digital health" WN KY OR (("application*" WN KY OR "app" WN KY OR "apps" WN	76	2021.10.08

	KY) AND health* WN KY)) AND (("post-adoption" WN KY OR "post-adoptive " WN KY OR "continuance" WN KY OR "continue to use" WN KY OR "continue using" WN KY OR "continued" WN KY OR "continuous" WN KY OR "sustained" WN KY OR "stickiness" WN KY) AND "intention" WN KY)		
ACM	[[Abstract: "mHealth"] OR [Abstract: "m-health"] OR [Abstract: "mobile health"] OR [Abstract: "telemedicine"] OR [Abstract: "telehealth"] OR [Abstract: "ehealth"] OR [Abstract: "e-health"] OR [Abstract: "electronic health"] OR [Abstract: "uHealth"] OR [Abstract: "ubiquitous health"] OR [Abstract: " digital medical"] OR [Abstract: "digital medicine"] OR [Abstract: "digital health"] OR [[[Abstract: "application*"] OR [Abstract: "app"] OR [Abstract: "apps"]] AND [Abstract: health*]]] AND [[Abstract: "post-adoption"] OR [Abstract: "post-adoptive"] OR [Abstract: "continuance"] OR [Abstract: "continue to use"] OR [Abstract: "continue using"] OR [Abstract: "continued"] OR [Abstract: "continuous"] OR [Abstract: "sustained"] OR [Abstract: "stickiness"]] AND [Abstract: "intention"]	5	2021.10.08
Scopus	TITLE-ABS-KEY(mHealth) OR TITLE-ABS-KEY("m-health") OR TITLE-ABS-KEY("mobile health") OR TITLE-ABS-KEY(Telemedicine) OR TITLE-ABS-KEY(Telehealth) OR TITLE-ABS-KEY("Tele-health") OR TITLE-ABS-KEY(ehealth) OR TITLE-ABS-KEY("e-health") OR TITLE-ABS-KEY("electronic health") OR TITLE-ABS-KEY("uHealth") OR TITLE-ABS-KEY("ubiquitous health") OR TITLE-ABS-KEY("digital medical") OR TITLE-ABS-KEY("digital medicine") OR TITLE-ABS-KEY("digital health") OR ((TITLE-ABS-KEY("application*") OR TITLE-ABS-KEY("app") OR TITLE-ABS-KEY("apps")) AND TITLE-ABS-KEY(health*)) AND ((TITLE-ABS-KEY("post-adoption") OR TITLE-ABS-KEY("post-adoptive") OR TITLE-ABS-KEY("continuance") OR TITLE-ABS-KEY("continue to use") OR TITLE-ABS-KEY("continue using") OR TITLE-ABS-KEY("continuous") OR TITLE-ABS-KEY("continued") OR TITLE-ABS-KEY("sustained") OR TITLE-ABS-KEY("stickiness")) AND TITLE-ABS-KEY("intention"))	365	2021.10.08
embase	('m-health':ab OR 'mHealth':ab OR 'mobile health':ab OR 'Telemedicine':ab OR 'Telehealth':ab OR 'Tele-health':ab OR 'ehealth':ab OR 'electronic health':ab OR 'uHealth':ab OR 'ubiquitous health':ab OR 'digital medical':ab OR 'digital	108	2021.10.08

	medicine':ab OR 'digital health':ab OR (('application*':ab OR 'app':ab OR 'apps':ab) AND health*:ab)) AND (('post-adoption':ab OR 'post-adoptive':ab OR 'continuance':ab OR 'continue to use':ab OR 'continue using':ab OR 'continued':ab OR 'continuous':ab OR 'sustained':ab OR 'stickiness':ab) AND 'intention':ab)		
CINAHL	((AB "m-health" OR AB “mHealth” OR AB "mobile health" OR AB "Telemedicine" OR AB "Telehealth" OR AB "Tele-health" OR AB "ehealth" OR AB "electronic health" OR AB "uHealth" OR AB "ubiquitous health" OR AB "digital medical" OR AB "digital medicine" OR AB "digital health") OR ((AB "application*" OR AB "app" OR AB "apps") AND (AB health*))) AND ((AB "post-adoption" OR AB "post-adoptive " OR AB "continuance" OR AB "continue to use" OR AB "continue using" OR AB "continued" OR AB "continuous" OR AB "sustained" OR AB "stickiness") AND (AB "intention"))	38	2021.10.08
PsycInfo	((AB "m-health" OR AB “mHealth” OR AB "mobile health" OR AB "Telemedicine" OR AB "Telehealth" OR AB "Tele-health" OR AB "ehealth" OR AB "electronic health" OR AB "uHealth" OR AB "ubiquitous health" OR AB "digital medical" OR AB "digital medicine" OR AB "digital health") OR ((AB "application*" OR AB "app" OR AB "apps") AND (AB health*))) AND ((AB "post-adoption" OR AB "post-adoptive " OR AB "continuance" OR AB "continue to use" OR AB "continue using" OR AB "continued" OR AB "continuous" OR AB "sustained" OR AB "stickiness") AND (AB "intention"))	32	2021.10.08
