# Supplementary material for: Identifying major impact factors affecting the continuance intention of mHealth: a systematic review and multi-subgroup meta-analysis
Source: NPJ Digit Med. 2022 Sep 15;5:145. doi: 10.1038/s41746-022-00692-9 (PMC9476418; doi:10.1038/s41746-022-00692-9)
Supplement: Supplementary file 2 — Reporting Summary [file 41746_2022_692_MOESM2_ESM.pdf]

nature portfolio

Corresponding author(s): Jianbo Lei, Hongbin Han

Last updated by author(s): Aug 26, 2022

Reporting Summary

Nature Portfolio wishes to improve the reproducibility of the work that we publish. This form provides structure for consistency and transparency in reporting. For further information on Nature Portfolio policies, see our Editorial Policies and the Editorial Policy Checklist.

Statistics

For all statistical analyses, confirm that the following items are present in the figure legend, table legend, main text, or Methods section.

n/a	Confirmed	
☐	☒	The exact sample size (*n*) for each experimental group/condition, given as a discrete number and unit of measurement
☐	☒	A statement on whether measurements were taken from distinct samples or whether the same sample was measured repeatedly
☐	☒	The statistical test(s) used AND whether they are one- or two-sided<br>*Only common tests should be described solely by name; describe more complex techniques in the Methods section.*
☒	☐	A description of all covariates tested
☒	☐	A description of any assumptions or corrections, such as tests of normality and adjustment for multiple comparisons
☐	☒	A full description of the statistical parameters including central tendency (e.g. means) or other basic estimates (e.g. regression coefficient) AND variation (e.g. standard deviation) or associated estimates of uncertainty (e.g. confidence intervals)
☐	☒	For null hypothesis testing, the test statistic (e.g. *F*, *t*, *r*) with confidence intervals, effect sizes, degrees of freedom and *P* value noted<br>*Give P values as exact values whenever suitable.*
☒	☐	For Bayesian analysis, information on the choice of priors and Markov chain Monte Carlo settings
☒	☐	For hierarchical and complex designs, identification of the appropriate level for tests and full reporting of outcomes
☐	☒	Estimates of effect sizes (e.g. Cohen's *d*, Pearson's *r*), indicating how they were calculated

Our web collection on statistics for biologists contains articles on many of the points above.

Software and code

Policy information about availability of computer code

Data collection	This paper was a meta-analysis and systematic review. The retrieved papers was directly exported from the database. A standardized data collection form was used to extract data which was an excel table.
Data analysis	We used SPSS statistical software version 24 and meta-essentials 1.1 for all statistical analyses.

For manuscripts utilizing custom algorithms or software that are central to the research but not yet described in published literature, software must be made available to editors and reviewers. We strongly encourage code deposition in a community repository (e.g. GitHub). See the Nature Portfolio guidelines for submitting code & software for further information.

Data

Policy information about availability of data

All manuscripts must include a data availability statement. This statement should provide the following information, where applicable:

- Accession codes, unique identifiers, or web links for publicly available datasets
- A description of any restrictions on data availability
- For clinical datasets or third party data, please ensure that the statement adheres to our policy

The authors declare that all the data included in this study are available within the paper and its Supplementary Information files.

Human research participants

Policy information about studies involving human research participants and Sex and Gender in Research.

Reporting on sex and gender	This study is a meta-analysis and systematic review to explore the factors affecting the intention to continue using mobile health. The 58 studies included in the analysis did not provide enough data of users to analyze the impact of gender or sex on the results. Therefore, this paper did not involve gender or sex related data.
Population characteristics	See above.
Recruitment	This study was a meta-analysis and systematic review, and did not involve the recruitment process of participants.
Ethics oversight	No ethical approval or informed consent was required for the current systematic review and meta-analysis.

Note that full information on the approval of the study protocol must also be provided in the manuscript.

Field-specific reporting

Please select the one below that is the best fit for your research. If you are not sure, read the appropriate sections before making your selection.

☐ Life sciences ☒ Behavioural & social sciences ☐ Ecological, evolutionary & environmental sciences

For a reference copy of the document with all sections, see nature.com/documents/nr-reporting-summary-flat.pdf

Behavioural & social sciences study design

All studies must disclose on these points even when the disclosure is negative.

Study description	Meta analysis and systematic review of quantitative cross-sectional studies on the continuance intention of mobile health.
Research sample	A systematic literature search for cross-sectional studies up to October 8, 2021, was conducted in the databases, including PubMed, Embase, WOS core collection, CINAHL, Scopus, PsycInfo, EI and ACM. We identified 1030 articles in 8 databases. After duplicate articles were removed, 470 articles remained. After applying further exclusion criteria, a total of 58 cross-sectional studies were included in our analysis.
Sampling strategy	A systematic literature search for cross-sectional studies up to October 8, 2021, was conducted in the databases, including PubMed, Embase, WOS core collection, CINAHL, Scopus, PsycInfo, EI and ACM. The retrieval formula consisted of three parts as follows: mHealth, continuance and intention. The search had no language restriction. We also searched and reviewed the references cited within the retrieved relevant reports for any additional studies.
Data collection	This paper was a meta-analysis and systematic review. A standardized data collection form was used to extract data which was an excel table.From each included study, we extracted the following information: first author, publication year, paper title, mHealth type, user type and age characteristics, sample size, country or region where the study was conducted, statistical methods, and independent and dependent variables, regression coefficient, P value and other statistical indices. In some of the included studies, the hypothesis of influence relationship is based on the relevant theories of psychology or behavior, so we also collected the theories or models on which the hypothesis is based. Two independent investigators (Tong Wang and Mingfu Nuo) performed the data extraction process, and any disagreements were resolved by group discussion.
Timing	A systematic literature search for cross-sectional studies up to October 8, 2021, was conducted in the databases, including PubMed, Embase, WOS core collection, CINAHL, Scopus, PsycInfo, EI and ACM.
Data exclusions	We identified 1030 articles in 8 databases. After duplicate articles were removed, 470 articles remained. After applying further exclusion criteria, a total of 58 cross-sectional studies were included in our analysis. Studies were included in the current meta-analysis if they met the following criteria: (1) the full text can be obtained. (2) the main outcome of the study was the continuance intention.(3) the type of study had to be quantitative. (4) the research object was mHealth related products or services.We excluded studies if they met the following criteria: (1) the studies did not show regression coefficients between the variables (research methods were not based on correlation analysis or regression analysis). (2) Reviews, letters, comments, editorial. (3) The studies were not published under a peer-review process.
Non-participation	This study was a meta analysis and systematic review of quantitative cross-sectional studies, and did not involve the dropout of participants.
Randomization	This study was a meta-analysis and systematic review, and did not involve randomization.

Reporting for specific materials, systems and methods

We require information from authors about some types of materials, experimental systems and methods used in many studies. Here, indicate whether each material, system or method listed is relevant to your study. If you are not sure if a list item applies to your research, read the appropriate section before selecting a response.

Materials & experimental systems

n/a	Involved in the study
☒	☐ Antibodies
☒	☐ Eukaryotic cell lines
☒	☐ Palaeontology and archaeology
☒	☐ Animals and other organisms
☒	☐ Clinical data
☒	☐ Dual use research of concern

Methods

n/a	Involved in the study
☒	☐ ChIP-seq
☒	☐ Flow cytometry
☒	☐ MRI-based neuroimaging
